# Comparative Patterns of Sex Expression and Sex Ratios in Island and Continental Bryophyte Populations

**DOI:** 10.3390/plants14040573

**Published:** 2025-02-13

**Authors:** Anabela Martins, Jairo Patiño, Manuela Sim-Sim

**Affiliations:** 1 cE3c—Centre for Ecology, Evolution and Environmental Changes & CHANGE—Global Change and Sustainability Institute/MUHNAC—Museu Nacional de História Natural e da Ciência, Universidade de Lisboa, Rua da Escola Politécnica, 58, 1250-102 Lisboa, Portugal; mmsim-sim@ciencias.ulisboa.pt; 2 Island Ecology and Evolution Research Group, Instituto de Productos Naturales y Agrobiología, Consejo Superior de Investigaciones Científicas (IPNA-CSIC), 38206 La Laguna, Spain; jpatino@ipna.csic.es; 3 cE3c—Centre for Ecology, Evolution and Environmental Changes & CHANGE—Global Change and Sustainability Institute, Departamento de Biologia Vegetal, Faculdade de Ciências, Universidade de Lisboa, Campo Grande, 1749-016 Lisboa, Portugal

**Keywords:** unisexual species, sex expression, sex ratio, Macaronesia, pleurocarpous mosses, leafy liverworts

## Abstract

Reproductive biology patterns are crucial for understanding the dynamics and evolution of plants. This is particularly relevant in bryophytes, where sex expression and reproductive success can vary significantly with environmental conditions. Islands, with their isolated and diverse environments, provide natural laboratories to explore these dynamics. In this study, we investigate sex expression, the phenotypic sex ratio, and sporophyte production in one moss (*Exsertotheca intermedia*) and three liverwort species (*Frullania polysticta*, *Frullania teneriffae*, *Porella canariensis*) across their entire distribution range. Depending on the species, the geographic range includes the Canary Islands, Madeira, the Azores, the Iberian Peninsula, the British Isles, and the Faroe Islands. For the non-Macaronesian endemic species (*F. teneriffae*, *P. canariensis*) higher levels of sex expression and males were found in the Macaronesian archipelagos. In leafy liverworts, females appear to be correlated with lower temperatures and higher precipitation levels, while males seem to be associated with higher temperatures and relatively lower precipitation levels. In this study, we demonstrated that bryophyte populations from Macaronesia exhibited higher levels of sex expression compared to their continental counterparts, suggesting that the distinct environmental conditions of these islands play a crucial role in shaping their reproductive patterns.

## 1. Introduction

Island biotas, with their isolation and environmental diversity, have long fascinated biologists [1]. While the comparison of island and continental plant populations offer tremendous potential for studying ecological and evolutionary patterns [2,3,4], comparative research on these geological settings remains less developed in plants compared to the vast body of literature on animal groups such as birds [5,6], insects [7], lizards [8], and mammals [9]. Investigating the reproductive biology of plants is crucial for understanding their population dynamics and the evolutionary processes that shape their diversity [10].

Bryophytes provide an excellent model for studying ecological and evolutionary patterns across island and continental regions, as their sexual expression and reproductive success are highly sensitive to environmental conditions [11,12,13]. These reproductive variations lead to significant differences in life history traits (LHTs) between oceanic islands and mainland regions [14]. For example, the increased production of specialized asexual diaspores and reduced sporophyte production on islands suggest a reduction in long-distance dispersal (LDD) ability, highlighting how typical island syndromes, such as the mentioned loss of dispersal power, could shape bryophyte floras on oceanic islands [14].

It is, however, noteworthy that unisexual bryophyte species often face challenges in reproducing sexually [15], as this process requires the presence of both male and female gametophytes nearby for successful fertilization [16]. This is particularly interesting because the requirement for both sexes is crucial to understanding reproductive success in isolated island environments. Consequently, asexual reproduction strategies, such as the production of specialized asexual diaspores, propagation, and clonal fragmentation, become crucial for their survival [15,17]. These dispersal mechanisms promote local population expansion over relatively short distances [14,18], playing a vital role in population persistence and reinforcement in isolated or fragmented habitats with low migration rates [15,17]. There is also evidence that asexual reproduction and the production of vegetative propagules contribute significantly to LDD, helping preserve well-adapted phenotypes in environmentally stable habitats [19]. In contrast, sexual reproduction recombines genetic variability, increasing the population’s chances of survival in new colonizing areas or during periods of environmental change [20].

In contrast to flowering plants, most unisexual bryophytes exhibit a female bias among sex-expressing individuals [21,22,23] and a significant proportion of unisexual species are found on islands [14]. Several hypotheses have been proposed to explain skewed sex ratios towards the female sex. Among these, some studies have observed that the female bias increases in warmer and wetter environments, which could be due to higher resource demands for sporophyte production in females or higher male sensitivity to wetness [13,18]. Additionally, higher rates of female clonal growth in the juvenile stage [18,22] may contribute to these skewed ratios. Other studies indicate that the female bias stems from the lower costs of producing sexual organs for female individuals compared to the prezygotic reproductive effort experienced by male individuals due to the greater allocation of biomass to sexual organs [12,16,24,25], which could compromise their survival and growth rates, further contributing to this female bias [18].

In this study, we explored patterns of sex expression and sex ratio variation in four unisexual bryophyte species to understand their implications for effective sexual reproduction. Our investigation focused on two threatened species endemics to Macaronesia: a pleurocarpous moss (*Exsertotheca intermedia* (Brid.) S.Olsson, Enroth & D.Quandt) and a leafy liverwort (*Frullania polysticta* Lindenb.). Additionally, we examined two leafy liverworts primarily restricted to Macaronesia and the European Atlantic fringe (*Frullania teneriffae* (F.Weber) Nees, *Porella canariensis* (F.Weber) Underw.). By studying these species across their entire range, we aimed to address the following questions: (1) How do sex expression and sex ratio in unisexual bryophyte species differ between island and continental populations? Given the distinct environmental conditions on islands, such as differences in temperature, precipitation and elevation [26,27] that can affect sex expression and sex ratio (e.g., [28,29]), we expect different patterns of sex expression and the sex ratio between oceanic islands and continental areas (H1a). Furthermore, we hypothesize that environmental factors (e.g., temperature, precipitation, and elevation) play significant roles in determining sex expression and sex ratio patterns (H1b). (2) Is there a correlation between the availability of male gametophytes and sporophyte production in the studied species, assuming that the populations are female-biased? Since the relationship between male gametophytes and sporophyte production can vary among species and environments [11,12,13], we investigated whether the male gametophytes represent a limitation for sporophyte production in the studied species (H2).

## 2. Results

### 2.1. Sex Expression and Phenotypic Sex Ratio

**Figure 1:**
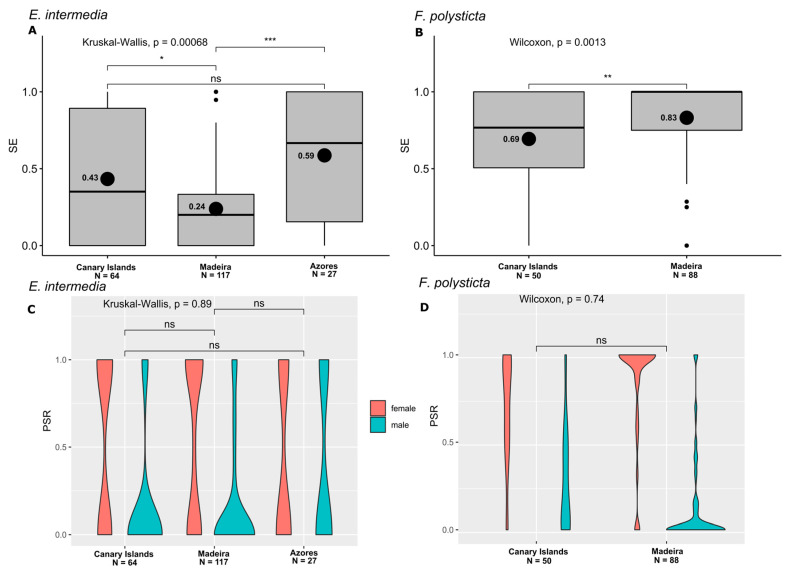
(**A**,**B**) Box plots with Kruskal–Wallis and Wilcoxon tests, displaying sex expression (SE) results for each species and region. Horizontal black lines denote median values, and the mean value is represented by a black dot the respective number displayed on the left. (**C**,**D**) Bean plots with Kruskal–Wallis and Wilcoxon tests, displaying phenotypic sex ratio (PSR) results for each species and region (*** *p* < 0.001, ** *p* < 0.01, * *p* < 0.05, ns indicates a non-significant difference). N, number of samples analyzed per locality. (**A**,**C**) *Exsertotheca intermedia*. (**B**,**D**) *Frullania polysticta*.

The highest values of SE were observed in *F. polysticta* (84% SE, with 382 nonexpressed shoots and 2052 expressed shoots), followed by *F. teneriffae* (66% SE, with 2607 nonexpressed shoots and 5126 expressed shoots), *P. canariensis* (59% SE, with 3348 nonexpressed shoots and 4788 expressed shoots), and *E. intermedia* (30% SE, with 1465 nonexpressed shoots and 628 expressed shoots). Regarding Macaronesia endemic species at the regional level, the pleurocarpous moss *E. intermedia* displayed higher average values of SE in the Azores (59%), followed by the Canary Islands (43%), and Madeira (24%) (Figure 1A).

The leafy liverwort *F. polysticta* had an average of 83% SE in Madeira and 69% in the Canary Islands (Figure 1B). The PSR of *E. intermedia* (Figure 1C, Table S1) exhibited a strong female bias in the Canary Islands (81%, χ^2^ = 73.4, *p* < 2.2 × 10^−16^) and Madeira (77%, χ^2^ = 111.1, *p* < 2.2 × 10^−16^). In the Azores, a balanced PSR was observed (42% PSR♀, χ^2^ = 1.6, *p* = 0.211). The overall PSR of *E. intermedia* exhibited a female bias (75%, χ^2^ = 131.6, *p* < 2.2 × 10^−16^). *Frullania polysticta* showed a female-biased PSR (Figure 1D, Table S5) in Madeira (85%, χ^2^ = 608.9, *p* < 2.2 × 10^−16^) and the Canary Islands (77%, χ^2^ = 247.5, *p* < 2.2 × 10^−16^).

**Figure 2:**
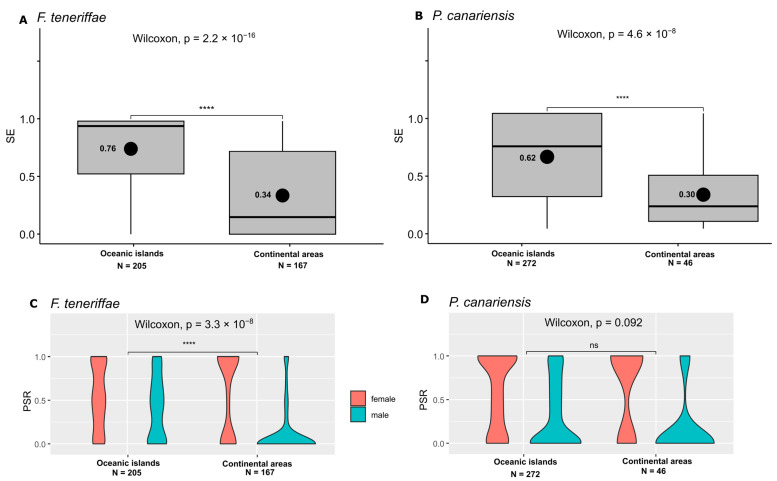
(**A**,**B**) Box plots with Wilcoxon tests, displaying sex expression (SE) results for each species and region (**** *p* < 0.0001, ns indicates a non-significant difference). Horizontal black lines denote median values, and the mean value is represented by a black dot the respective number displayed on the left. (**C**,**D**) Bean plots with Kruskal–Wallis and Wilcoxon tests, displaying phenotypic sex ratio (PSR) results for each species and region (**** *p* < 0.0001, ns indicates a non-significant difference). N, number of samples analyzed per locality. (**A**,**C**) *Frullania teneriffae*. (**B**,**D**) *Porella canariensis*.

The non-Macaronesian endemic species *F. teneriffae* exhibited an average of 76% SE on oceanic islands and 34% in continental areas (Figure 2A and Figure S1A). Similarly, *P. canariensis* showed an average SE of 62% on oceanic islands and 30% in continental areas (Figure 2B and Figure S1B).

*Frullania teneriffae* exhibited a balanced PSR on oceanic islands (50%, χ^2^ = 0.5, *p* = 0.479), (Figure 2C; Table S5) and a female-biased PSR (Figure 2C, Table S5) in the continental areas (80%, χ^2^ = 481.96; *p* < 2.2 × 10^−16^). Overall, the PSR of *F. teneriffae* was female-biased (57%, χ^2^ = 99.6, *p* < 2.2 × 10^−16^), (Figure S1C; Table S1). A significant difference in the PSR was observed between oceanic islands and continental areas (Figure 2C). *P. canariensis* demonstrated a female-biased PSR (Figure 2D, Table S5) on oceanic islands (57%, χ^2^ = 90.9, *p* < 2.2 × 10^−16^), and continental areas (61%, χ^2^ = 104.5, *p* < 2.2 × 10^−16^). Notably, a balanced PSR was observed in the Canary Islands (48% PSR♀, χ^2^ = 2.5, *p* = 0.116) (Figure S1D; Table S5).

**Figure 3:**
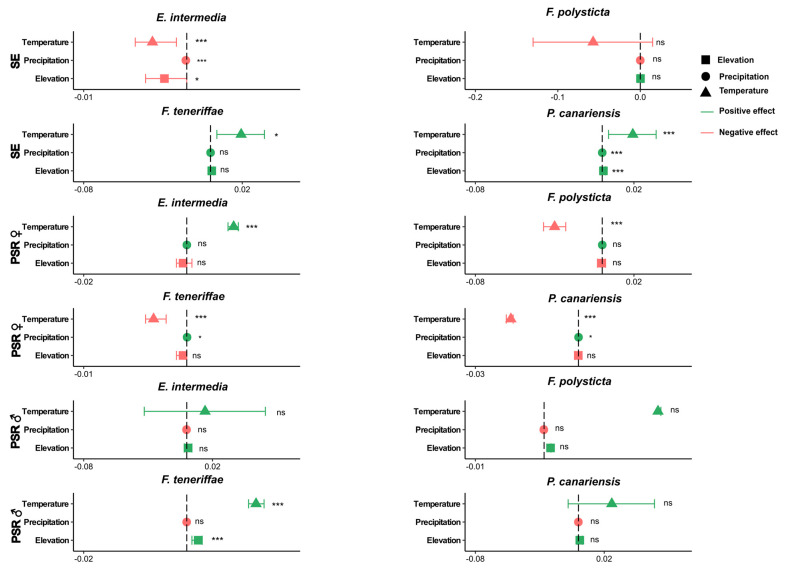
Forest plot depicting the effect of elevation, temperature and precipitation (coefficient, with 95% confidence interval) on sex expression (SE) and phenotypic female-to-male sex ratio (PSR♀ and PSR♂) for each species, derived from Generalized Linear Mixed Models (GLMMs) (*** *p* < 0.001, * *p* < 0.05, ns indicates a non-significant difference).

Concerning the relationship between SE and the PSR♂ (Figure 2 and Figure 3), there were significant positive correlations for *E. intermedia* (Spearman’s rho = 0.316, *p* = 3.431 × 10^−6^), *F. teneriffae* (Spearman’s rho = 0.515, *p* < 2.2 × 10^−16^ and *P. canarienis* (Spearman’s rho = 0.436, *p* = 3.362 × 10^−16^). SE and the PSR♂ were not significantly correlated for *F. polysticta* (Spearman’s rho = −0.036, *p* = 0.675).

### 2.2. Effects of Environment Factors on Sex Expression and Phenotypic Sex Ratio

Temperature and precipitation emerged as the most important factors, significantly affecting SE and PSR patterns (Figure 3, Table S2). Temperature had a positive effect on the SE of *F. teneriffae* and *P. canariensis*, as well as on the PSR♀ of *E. intermedia* and the PSR♂ of *F. teneriffae*. In contrast, it had a negative effect on the SE of *E. intermedia* and the PSR♀ of *F. polysticta*, *F. teneriffae*, and *P. canariensis* (Figure 3, Table S2). Precipitation had a positive effect on the SE of *P. canariensis* and PSR♀ of *F. teneriffae* and *P. canariensis*. In contrast, it exhibited a negative effect on the SE of *E. intermedia* (Figure 3, Table S2).

Elevation showed a positive effect on the SE of *P. canariensis*, as well as on the PSR♂ of *F. teneriffae*. It also had a negative effect on the SE of *E. intermedia* (Figure 3, Table S2).

### 2.3. Relationship Between Phenotypic Male Sex Ratio and Frequency of Sporophytes

**Figure 4:**
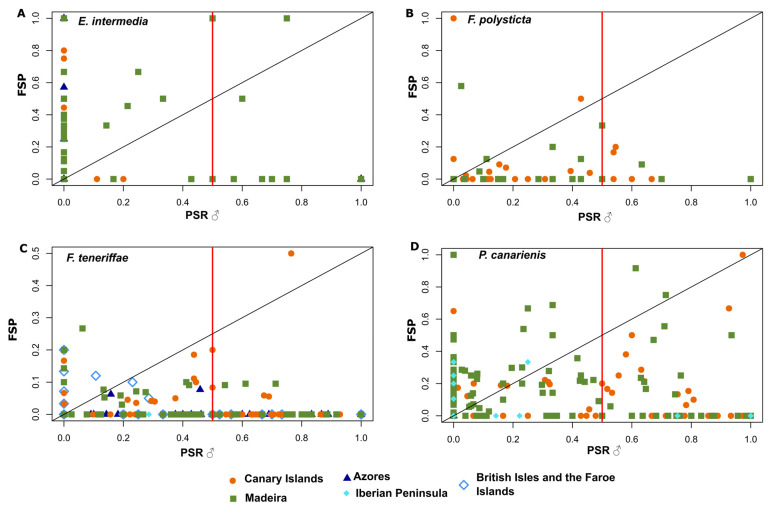
A binomial plot showing the phenotypic male sex ratio (PSR♂) of each studied species across regions and the frequency of sporophytes (FSP). The red line separates the samples with PSR♂ lower or higher than 0.5. (**A**) *Exsertotheca intermedia*. (**B**) *Frullania polysticta*. (**C**) *Frullania teneriffae*. (**D**) *Porella canariensis*.

For the moss *E. intermedia*, 48 (23%) samples were found with sporophytes (9 (15%) in the Canary Islands, 35 (30%) in Madeira, and 4 (15%) in the Azores). In the Canary Islands and Azores, shoots with sporophytes were only found in populations with a PSR♂ = 0. In Madeira, 77% of samples with sporophytes were found in populations with a PSR♂ = 0 and 94% with a PSR♂ ≤ 0.5 (Figure 4A). The PSR♂ and FSP were not significantly correlated for *E. intermedia* (Spearman’s rho = −0.013, *p* = 0.064), although there was a tendency for a negative correlation.

The liverwort *F. polysticta* showed 18 (13%) of samples with sporophytes (11 (22%) in the Canary Islands, 7 (8%) in Madeira). In the Canary Islands and Madeira, 82% and 57% of samples with sporophytes were found in populations with a PSR♂ ≤ 0.5, respectively (Figure 4B). However, the PSR♂ and FSP were significantly positively correlated (Spearman’s rho = 0.283, *p* < 0.001). *F. teneriffae* revealed 42 (11%) of samples with sporophytes (18 (22%) in the Canary Islands, 15 (20%) in Madeira, 2 (4%) in the Azores, 7 (5%) in the British Isles and the Faroe Islands). In the Canary Islands, Madeira, the Azores, the British Isles and the Faroe Islands, 83%, 80%, 100% and 100% of samples with sporophytes were observed in populations with a PSR♂ ≤ 0.5, respectively. No sporophyte shoots were observed in the Iberian Peninsula samples (Figure 4C). The PSR♂ and FSP were not significantly correlated for *F. teneriffae* (Spearman’s rho = 0.068, *p* = 0.191).

Finally, in the liverwort *P. canariensis*, 105 (33%) samples were found with sporophytes (30 (24%) in the Canary Islands, 70 (58%) in Madeira, and 5 (11%) in the Iberian Peninsula). In the Canary Islands, Madeira and the Iberian Peninsula, 57%, 84% and 100% of samples producing sporophytes were observed in populations with a PSR♂ ≤ 0.5, respectively (Figure 4D). No sporophyte shoots were found in the Azores samples (Figure 4D). The PSR♂ and FSP were not significantly correlated for *P. canariensis* (Spearman’s rho = 0.044, *p* = 0.430).

## 3. Materials and Methods

### 3.1. Study Area and Floristic Affinities

**Figure 5:**
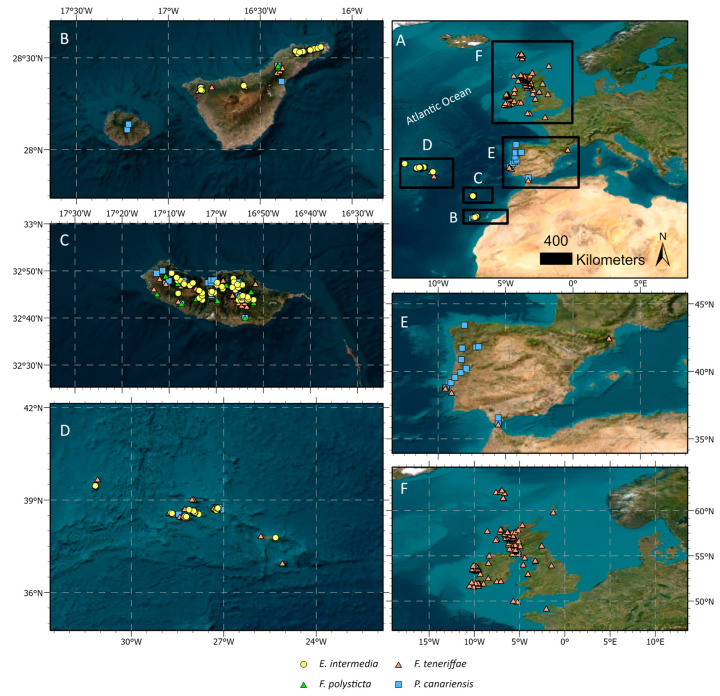
Maps showing the location of the samples of our studied species (yellow circle: *Exsertotheca intermedia*, green triangle: *Frullania polysticta*, pink triangle: *Frullania teneriffae*, blue square: *Porella canariensis*) across the five regions. (**A**) An overall view of the sampling area, including the Macaronesia (Oceanic islands), which contains (**B**) the Canary Islands; (**C**) Madeira Island; (**D**) and the Azores archipelago, as well as the European Atlantic fringe (continental areas): (**E**) the Iberian Peninsula; (**F**) the British Isles; and the Faroe Islands.

The present study was conducted in Macaronesia and the European Atlantic fringe (Figure 5).

Macaronesia, comprising the Canary Islands, Madeira, Azores, and Cape Verde archipelagos [30], is part of the 12 biodiversity hotspots identified by Conservation International in 2005 [31]. The flora of Macaronesia exhibits diverse affinities and levels of connectivity, primarily based on three distinct floristic components shared between the Macaronesian core (Madeira and the Canary Islands) and the outermost archipelagos (the Azores and Cape Verde) [30]. One significant component is the Palaeotropical Tethyan Geoflora, which was once widespread across Europe and North Africa but is now confined to the northern archipelagos (the Azores, Madeira, and the Canary Islands). Another important component is the African Rand Flora [30,32], which persists along the coastal margins of Africa and Arabia, influencing the southern archipelagos of Madeira, the Canary Islands, and Cape Verde. Additionally, the Macaronesian Neoendemic Flora [33] spans all archipelagos and arises from the allopatric diversification of Mediterranean ancestors, resulting in unique evolutionary pathways in response to varying island environments and geographic isolation [30].

The European Atlantic fringe encompasses coastal regions along the Atlantic Ocean, stretching from the northern coasts of Iberian Peninsula to the British Isles, Ireland, and the Faroe Islands [34,35]. Floristically, the European Atlantic fringe is characterized by a distinctive set of plants that form the Atlantic element, primarily associated with coastal ecosystems and heathlands [35].

### 3.2. Study Species

The pleurocarpous moss *Exsertotheca intermedia* is a Macaronesian endemic species (Figure 1), found across all the archipelagos. It is a threatened species classified as Vulnerable on the European Red List of Bryophytes [36] due to threats such as climate change, intensified fires, and invasive species. This species can grow as an epiphyte, on humid rocks, and along stream banks in the natural laurel forest mainly between 50 m and 1900 m (a.s.l.) [36,37]. Sexual reproduction is frequent [38].

The leafy liverwort *Frullania polysticta* is endemic to the archipelagos of Madeira and the Canary Islands (Figure 1). *Frullania polysticta* is a threatened species classified as Vulnerable on the European Red List of Bryophytes due to habitat destruction caused by urbanization and tourism, climate change, and fire [36,39]. This species mainly grows on tree bark and shaded rocks in well-preserved laurel forest areas. It is typically found between 500 m and 1200 m (a.s.l.), with the highest frequency above 800 m (a.s.l.). Asexual reproduction is unknown [39,40].

*Frullania teneriffae* is a hyperoceanic southern-temperate leafy liverwort, mainly restricted to Macaronesia and the European Atlantic fringe, recorded from western Britain, Ireland, the Faroe Islands, Portugal, France, Spain, Macaronesia, and Turkey [41,42,43]. It is classified as Least Concern on the European Red List of Bryophytes [36]. In this study, only samples from Turkey were not analyzed. This species inhabits dry siliceous rocks near the sea in western Scotland and Ireland (0–500 m a.s.l.), thrives in humid and shaded forests in the Iberian Peninsula, and is often found in the laurel forests of Macaronesia (900–1300 m a.s.l.) [40,43,44]. Asexual reproduction is unknown [40].

The leafy liverwort *Porella canariensis* is restricted to Macaronesia and the European Atlantic fringe, occurring in all Macaronesia archipelagos, the Iberian Peninsula, and France [41]. It is classified as Least Concern on the European Red List of Bryophytes [36]. In this study, only samples from France were not analyzed. In Macaronesia, *P. canariensis* commonly grows as epiphyte on rocks and slopes in sheltered habitats up to 1400 m (a.s.l). In the Iberian Peninsula, it occurs similarly in habitats within coastal mountainous regions at medium altitudes. Sexual reproduction is frequent, and vegetative propagation may occur [45,46].

### 3.3. Sampling Design and Data Collection

Fieldwork was conducted on the islands of Madeira and Tenerife, as well as on the Sintra mountain (mainland Portugal), between October and December 2022 (Table S3). A total of 37 sampling sites were randomly selected based on the distribution ranges of the four species (18 Madeira, 15 Tenerife, and 4 Sintra). The sampling sites were separated by a minimum distance of 100 m. At each site, we established a circular plot of 314 m^2^ (radius 10 m) and selected five trees (per species) where the targeted species were present. For each site, we recorded the geographical position to an accuracy of 5–8 m with a GPS (latitude and longitude) and elevation (m a.s.l.). From each of the five selected trees, a sample was collected within a 5 × 5 cm plot. In total, 125 samples of *E. intermedia* (85 from Madeira and 40 from Tenerife), 75 samples of *F. polysticta* (55 from Madeira and 20 from Tenerife), 70 samples of *F. teneriffae* (30 from Madeira, 30 from Tenerife, and 10 from Sintra), and 155 samples of *P. canariensis* (85 from Madeira, 60 from Tenerife, and 10 from Sintra) were collected. The samples were individually placed in glassine bags, dried, and stored at room temperature until further treatment in the laboratory. A voucher of each species collected was deposited in the LISU herbarium (Museu Nacional de História Natural e da Ciência, Universidade de Lisboa).

Additionally, to enhance our study and encompass the entire range of species (Figure 1), we also examined herbarium material (Lund Botanical Museum (LD), Museu Nacional de História Natural e da Ciência, Universidade de Lisboa (LISU), Royal Botanic Garden Edinburgh (E), Universidade dos Açores (AZU), Universidad de La Laguna (TFC), Universitat de València (VAL), and the University of Copenhagen (C)), applying the same 5 × 5 cm plot method used for the field samples. Thus, the study area encompasses five regions (see Figure 1), ranging latitudinally from the Canary Islands to the British Isles and the Faroe Islands, namely, the Canary Islands, Madeira Island, Azores archipelago, Iberian Peninsula (mainland Portugal and Spain, including France), British Isles and Faroe Islands (comprising Ireland, Great Britain, and the Faroe Islands). Duplicate specimens and multiple collections from the same locality were discarded. The specimens with complete label information were prioritized, including details such as location, geographic coordinates, and elevation. Specimens lacking geographical coordinates were georeferenced following the Guide to Best Practices for Georeferencing [47] and utilizing Google Earth Pro 7.3.3. For *E. intermedia,* 83 samples were additionally studied (27 the Azores, 24 the Canaries, and 32 Madeira Island), as well as 63 samples of *F. polysticta* (30 the Canaries and 33 Madeira Island). We also examined 302 herbarium samples of *F. teneriffae* (48 the Azores, 51 the Canaries, 46 Madeira Island, 17 mainland Portugal, 1 mainland Spain, 1 France, 45 Ireland, 80 Great Britain, and 13 Faroe Islands), as well as 163 herbarium samples of *P. canariensis* (27 the Azores, 61 the Canaries, 39 Madeira Island, 33 mainland Portugal, and 3 mainland Spain). Although *E. intermedia* and *P. canariensis* occur in Cape Verde, this region was not included in the study due to the insufficient availability of specimens.

### 3.4. Sex Determination

The samples obtained from both fieldwork and herbarium collections (1036) samples of *E. intermedia* (208: 64 the Canary Islands, 117 Madeira Island, and 27 the Azores), *F. polysticta* (138: 50 the Canary Islands and 88 Madeira Island), *F. teneriffae* (372: 81 the Canary Islands, 86 Madeira Island, 48 the Azores, 19 the Iberian Peninsula, and 138 the British Isles and the Faroe Islands), and *P. canariensis* (318: 121 the Canary Islands, 124 Madeira Island, 27 the Azores, and 46 the Iberian Peninsula, respectively) were observed in order to estimate the proportions of expressed male, female, and non-expressing shoots. All shoots within a 5 × 5 cm plot were carefully examined using a stereoscopic microscope (Olympus SZ40, Olympus Corporation; Lisbon, Portugal) and light microscopy (Olympus BX51, Olympus Corporation; Lisbon, Portugal). For the herbarium specimens, we examined the shoots found within the same 5 × 5 cm plot area. A total of 20,396 shoots were analyzed, distributed as follows: 2093 shoots of *E. intermedia* (an average of 11 shoots per sample), 2434 shoots of *F. polysticta* (an average of 18 shoots per sample), 8136 shoots of *F. teneriffae* (an average of 22 shoots per sample), and 7733 shoots of *P. canariensis* (an average of 24 shoots per sample). All shoots were categorized as sexually expressed males or females if they bore perigonia or perichaetia, respectively, while those lacking such features were classified as non-expressing shoots (see Figure S2).

The level of sex expression (SE) for all samples was determined by calculating the observed proportion of expressed shoots (the number of expressed shoots/the total number of shoots). The phenotypic female sex ratio (PSR♀) and the phenotypic male sex ratio (PSR♂) were calculated as the proportion of expressed shoots of either sex (the number of females or males/the sum of females and males). The frequency of sporophytes (FSP) was also analyzed, estimated as the number of shoots with well-developed sporophytes over the total number of detected female gametophyte shoots.

### 3.5. Data Analyses

Since the patterns of SE, the PSR♀ and the PSR♂ were consistent between herbarium samples and fieldwork samples, both datasets were combined (Table S4). Differences between the two sample types were tested using the Mann–Whitney–Wilcoxon test, and the significance levels were adjusted for multiple comparisons using Bonferroni corrections. Furthermore, we ensured that the sampling was evenly distributed across the seasons, with 43% of the samples collected in Spring and Summer and 57% in Autumn and Winter (Table S3). We used a Shapiro–Wilk normality test (Table S5) to assess whether the data followed a normal distribution or not, using the “stats” R package [48]. Additionally, for the fieldwork samples, we investigated whether the type of phorophyte (tree species) affected the patterns of SE, the PSR♀ and the PSR♂. The Kruskal–Wallis test from the “stats” R package [48] was used, but no such effect was observed (Table S6).

To determine significant differences in median SE, PSR♀ and PSR♂ values across the studied geographic regions, including the oceanic islands of Macaronesia (the Canary Islands, Madeira, and Azores), as well as the European Atlantic fringe (continental areas) (H1), Kruskal–Wallis tests and Wilcoxon tests were computed using the “rstatix” R package [49]. The Wilcoxon test was employed specifically for its ability to compare paired groups. Generalized Linear Models (GLMs) with binomial error distribution and logit link functions were computed (Table S1), using the “stats” R package [48], to test whether the number of samples per region (i.e., variation in sampling effort across regions) influences the SE, PSR♀ and PSR♂. The binomial model is well suited for proportion data, where the dependent variables represent the number of successes relative to the total number of independent observations [50]. Additionally, for each species and region, we tested whether sex ratios differed from an expected unbiased sex ratio (0.5) with Pearson’s χ^2^ tests. We also searched for a significant association between SE and the PSR♂, using the Spearman rank correlation coefficient, using the “stats” R package [48].

To evaluate whether elevation, precipitation, and temperature affected the SE, PSR♀, PSR♂, and FSP for each species (H2), we first extracted from CHELSA v. 2.1 [51] the mean annual precipitation and temperature at a 30-arc-second (~1 km) resolution. The values for each sample location were extracted using the ArcGIS 10.7 [52]. Then, we computed Pearson correlation coefficients among each pair of variables (elevation, precipitation, and temperature). The highest correlation coefficient was observed between precipitation and temperature (0.54), and there was no need to exclude any variables due to multicollinearity. Generalized Linear Mixed Models (GLMMs) with binomial error distribution and logit link functions were employed, using the “lme4” R package [53]. SE, the PSR♀, and the PSR♂ were treated as response variables, with elevation, precipitation, and temperature designated as fixed predictors. The geographic region was considered a random effect. Model selection, including the evaluation of the null model, was based on the Akaike Information Criterion (AIC). The direction of the effect was assessed based on the sign of standardized coefficients (Table S2).

To explore significant associations between the PSR♂ and FSP (H3), the Spearman rank correlation coefficient was used, using the “stats” R package [48]. All analyses were performed in R version 4.1.3 [48].

## 4. Discussion

Our results reveal that the island population of the species studied exhibits greater sexual expression compared to the continental populations, with a predominantly female-biased phenotypic sex ratio. Temperature and precipitation were key factors influencing this pattern, suggesting that sex-specific differences in environmental tolerance have shaped the geographic distribution of phenotypic sex ratios. Similar relationships between environmental factors and sex ratios have been observed in other bryophyte species [13,18] and in flowering plants [54], where environmental factors such as climate have been shown to drive shifts in sex ratios. Island species often exhibit altered reproductive strategies, such as biased sex ratios or increased sexual expression, as a response to island-specific selective pressures, including limited gene flow, small population sizes, and unique environmental constraints [55,56,57,58]. Our study adds to this growing body of literature by highlighting how insular bryophyte populations may be subjected to similar evolutionary pressures, leading to distinct reproductive adaptations.

### 4.1. Patterns of Sex Expression and Sex Ratio

In line with the initial expectations (H1a), different patterns of sex expression and sex ratio emerged between oceanic islands and continental areas. The non-Macaronesia endemic species (*F. teneriffae* and *P. canariensis*) exhibited heightened levels of SE in the northern Macaronesia archipelagos (the Canary Islands, Madeira, and Azores). Notably, a positive correlation between SE and the PSR♂ was observed in both species. The northern Macaronesia archipelagos are characterized by a predominantly subtropical Mediterranean to oceanic temperate climate, influenced by mid-Atlantic oceanic conditions that result in moderate precipitation and stable temperatures year round [59]. In contrast, the European Atlantic fringe, located at higher latitudes with distinct atmospheric circulation patterns, experiences greater climatic variability and higher precipitation, particularly during winter months [60]. The stable climatic conditions characteristic of the northern Macaronesia archipelagos may enable these species to allocate more energy to sexual reproduction, as reproductive costs in plants are known to increase under conditions of water scarcity [61] and elevated temperatures [62]. Furthermore, bryophytes rely on a continuous film of water for sperm to swim to eggs, making sexual reproduction highly dependent on adequate moisture levels [16,63].

The observed female-biased of *F. teneriffae* and *P. canariensis*, increased in the continental areas, a trend that can be attributed to the more fluctuating environmental conditions in these regions [35,60]. In contrast, male gametophytes are more dependent on stable environmental conditions [22,29,64,65,66,67,68], such as those found on the laurel forests. These forests are characterized by consistently high humidity levels year round, minimal temperature fluctuations, and increased microhabitat complexity [69,70], making them favorable environments for male gametophytes. In contrast, female gametophytes demonstrate greater flexibility and tolerance to environmental variability [29]. Interestingly, similar patterns can be seen in flowering plants, where male-biased sex ratios predominate in stable natural habitats [71]. This highlights the broader ecological trend where male reproductive lineages seem to be more dependent on favorable and stable conditions, while female reproductive structures exhibit greater resilience to environmental instability.

Regarding the Macaronesian endemic species, the leafy liverwort *F. polysticta* exhibited higher levels of SE on Madeira Island compared to the Canary Islands, while the pleurocarpous moss *E. intermedia* demonstrated higher levels of SE in the Azores. The higher levels of annual precipitation and reduced fluctuations in the Azores and Madeira, along with their lower mean annual temperatures compared to the Canary Islands [72], may provide more stable environments for these species, allowing them to allocate more energy towards sexual reproduction. *F. polysticta* maintained a female-biased sex ratio throughout its distribution, whereas *E. intermedia* exhibited the highest female bias in the Canary Islands, with a balanced sex ratio recorded in the Azores. However, no statistically significant differences in the PSR were detected among the Macaronesian archipelagos for these two endemic species. These patterns align with previous findings in several bryophyte species, which indicate that the female bias in the population sex ratio is significantly lower in areas recolonized after the Last Glacial Maximum compared to glacial refugia, suggesting that more recent populations tend to decrease skewed sex ratios [13,18,73,74].

### 4.2. Environmental Factors of Sex Expression and Sex Ratio

The SE reported in many bryophyte taxa and vascular plants in general may be associated with environmental restrictions, such as temperature, water availability [61,75,76,77] and light availability [78,79,80].

Consistent with our hypothesis H1b, temperature and precipitation emerged as influential factors affecting SE and PSR patterns. Temperature had a positive effect on the SE of *F. teneriffae* and *P. canariensis* but a negative effect on the SE of *E. intermedia*. Precipitation had a positive effect on the SE of *P. canariensis* and a negative effect on the SE of *E. intermedia*. The cooler temperatures in the Azores favor the SE of *E. intermedia*, while the higher temperatures in the Canary Islands influence the SE of *F. teneriffae*. Additionally, *P. canariensis* exhibits higher levels of SE in Madeira, which has higher precipitation compared to the Canary Islands [81,82]. The dynamics of temperature and precipitation effects on SE are crucial for predicting how these species might respond to climate change, as such variations can significantly impact their sex expression and, consequently, their reproductive success.

Temperature also had a positive effect on the PSR♀ of *E. intermedia*, as well as on the PSR♂ of *F. teneriffae*, and a negative effect on the PSR♀ of *F. polysticta*, *F. teneriffae* and *P. canariensis*. Precipitation showed a positive effect on the PSR♀ of both *F. teneriffae* and *P. canariensis*. The PSR♀ in the leafy liverworts appears to be correlated with lower temperatures and higher precipitation levels, while the PSR♂ seems to be associated with higher temperatures and lower precipitation levels.

Elevation showed a positive effect on the SE of *P. canariensis*, as well as on the PSR♂ of *F. teneriffae*, contrary to *Racomitrium lanuginosum* (Hedw.) Brid. where the number of male shoots decreased with elevation [29]. This pattern is opposite to that observed in *Juniperus* spp. (Cupressaceae) in Southeastern Spain, where the number of males increased with elevation [83]. Additionally, elevation also had a negative effect on the SE of *E. intermedia*. This suggests species-specific responses to altitude-related environmental gradients, which is particularly relevant for island populations.

Our findings highlight the complex interactions between environmental factors, sexual expression, and phenotypic sex ratios in bryophyte species. The observed variations emphasize the importance of considering regional differences and environmental contexts when studying reproductive strategies in bryophytes [13,74,84]. Understanding the mechanisms driving SE and PSR dynamics in unisexual bryophytes, especially threatened and endemic species, can provide valuable insights into their genetic variability, ecological adaptations, and responses to changing environmental conditions, with potential implications for biodiversity conservation and ecosystem management.

### 4.3. Influence of Expressed Sexual Ratios on Sexual Reproduction

Remarkably, in accordance with our hypothesis (H2), the relationship between male gametophytes and sporophyte production varies among species and environments [11,12,13]. In general, the probability of sexual reproduction was higher in populations with a PSR♂ ≤ 0.5. Although no significant correlation was found between the PSR♂ and FSP in *F. teneriffae* and *P. canariensis*, *E. intermedia* showed no significant correlation, with a tendency toward a negative correlation between the PSR♂ and FSP. Specifically, for *E. intermedia* in the Canary Islands and the Azores, sporophyte production was exclusively associated with populations with a PSR♂ = 0. It is, however, probable that male shoots were present and ensured the fertilization of female shoots. Alternatively, this could also result from the differing distances of fertilization at which the antheridia were located relative to the archegonia [85] in each of the sampled locations. This finding is consistent with previous studies [12,18], which have reported a negative relationship between the male sex ratio and sporophyte formation in the mosses *Ceratodon purpureus* (Hedw.) Brid. [12] and *Pseudoscleropodium purum* (Hedw.) M. Fleisch. [18]. The authors suggested that females might delay sporophyte production until they have outcompeted males in terms of clonal growth and reached a size threshold for sporophyte production. This indicates that stronger female-biased sex ratios could lead to greater female vegetative growth and a higher probability of successful sexual reproduction [18].

In contrast, the Macaronesian endemic leafy liverwort *F. polysticta* exhibited a positive correlation between the PSR♂ and FSP, suggesting that as the proportion of sexually reproductive males increased, the frequency of sporophytes also increased. The reduced sporophyte production in strongly female-biased populations was also reported in other species (e.g., *Drepanocladus lycopodioides* (Brid.) Warnst. [13] and *Frullania tamarisci* (L.) Dumort [11]. Sporophyte production imposes a significant reproductive cost for the female plant [86], which has been demonstrated to lead to reduced female performance and potentially elevated female mortality [87,88]. For instance, a modeling approach applied to the pleurocarpous moss *Hylocomium splendens* (Hedw.) Schimp. [89] suggests that a female-skewed sex ratio could persist if sporophyte production occurs infrequently.

Our results indicate that for the non-Macaronesian endemic species (*F. teneriffae* and *P. canariensis*) and the Macaronesian endemic species (*E. intermedia*), the probability of sporophyte production is higher in female-dominated populations. In contrast, the less widespread endemic species *F. polysticta* exhibits fewer sporophytes in strongly female-biased populations. These findings suggest that the relationship between male gametophytes and sporophyte production varies among species and environments, influenced by differing ecological and evolutionary factors [11,13,18,25].

## 5. Conclusions

Our findings on the four studied bryophyte species reveal distinct patterns of interaction between environmental factors, sexual expression, and phenotypic sex ratios in Macaronesian island populations compared to their continental counterparts. We observed that in the Macaronesian islands, there was an increase in sexual expression, suggesting that the distinct environmental conditions of these islands play a crucial role in shaping their reproductive patterns. Recognizing patterns in sex expression and phenotypic sex ratios in bryophytes is crucial for developing effective management strategies and conservation measures in line with the International Union for Conservation of Nature (IUCN) guidelines, which emphasize habitat preservation and genetic diversity. Additionally, future research anticipating the impacts of climate change on sex distribution in these plants is essential. Such studies will provide critical insights for developing proactive conservation efforts to protect vulnerable species, such as *E. intermedia* and *F. polysticta* from potential reproductive shifts and demographic changes driven by climate change.

## Data Availability

The original data presented in this study are included in the article and its Supplementary Materials. Further inquiries can be directed to the corresponding authors.

## Supplementary Materials

The following supporting information can be downloaded at: https://www.mdpi.com/article/10.3390/plants14040573/s1, Table S1: Number of studied samples, per species and region, the total SE and PSR. Table S2: Summary of the best Generalized Linear Mixed Models (GLMM). Table S3: Information for each sample, including location (longitude/latitude), source (herbarium or fieldwork), collection date, elevation, host tree, SE, PSR, and FSP. Table S4: The mean SE and PSR, per species and region for both herbarium and fieldwork samples. Table S5: Results of the Shapiro–Wilk normality test to determine whether the data follow a normal distribution or not. Table S6: Results of the Kruskal–Wallis test used to investigate whether the type of phorophyte (tree species) affected SE and PSR in the fieldwork samples. Figure S1: Box plots and bean plots showing SE and PSR for each species and region. (A,C) *Frullania teneriffae*; (B,D) *Porella canariensis*. Figure S2: Male and female shoots of the study species, illustrating their distinct morphological characteristics.
