# Supplementary material for: Comparative Patterns of Sex Expression and Sex Ratios in Island and Continental Bryophyte Populations
Source: Plants (Basel). 2025 Feb 13;14(4):573. doi: 10.3390/plants14040573 (PMC11859246; doi:10.3390/plants14040573)
Supplement: Supplementary file 1 [file plants-14-00573-s001.zip › plants-3456425-supplementary.pdf]

Comparative patterns of sex expression and sex ratios in island and continental bryophyte populations

Table S1. Number of studied samples, per species and region, the total sex expression (SE) and phenotypic sex ratio (PSR ♀: females; and ♂: males). Results of the Generalized Linear Models (GLM) assessing whether the number of samples per region has any influence on SE, PSR♀, and PSR♂ for each species are also shown. (ns) indicates a non-significant difference, meaning that the number of samples per region did not significantly influence the variable.

	**Region**	**Number of samples**	**SE**	**PSR♀**	**PSR♂**
E. intermedia	Canary Islands	64	0.37	0.81	0.19
	Madeira	117	0.26	0.77	0.23
	Azores	27	0.50	0.42	0.58
	All	208	0.30	0.75	0.25
		**p-value**	0.732 (ns)	0.639 (ns)	0.639 (ns)
F. polysticta	Canary Islands	50	0.78	0.77	0.23
	Madeira	88	0.89	0.85	0.15
	All	138	0.84	0.82	0.18
		**p-value**	0.837 (ns)	0.886 (ns)	0.886 (ns)
F. teneriffae	Canary Islands	81	0.92	0.46	0.54
	Madeira	76	0.75	0.56	0.44
	Azores	48	0.63	0.54	0.46
	Iberian Peninsula	29	0.19	0.64	0.36
	British Isles and the Faroe Islands	138	0.33	0.81	0.19
	All	372	0.59	0.57	0.43
		**p-value**	0.942 (ns)	0.766 (ns)	0.766 (ns)
	Oceanic islands	205	0.80	0.50	0.50
	Continental areas	167	0.31	0.80	0.20
		**p-value**	0.508 (ns)	0.665 (ns)	0.665 (ns)
	**Region**	**Number of samples**	**SE**	**PSR♀**	**PSR♂**
P. canariensis	Canary Islands	124	0.69	0.48	0.52
	Madeira	121	0.82	0.64	0.36
	Azores	27	0.18	1.00	0.00

	Iberian Peninsula	46	0.30	0.61	0.39
	All	318	0.66	0.57	0.43
		**p-value**	0.328 (ns)	0.545 (ns)	0.550 (ns)
	Oceanic islands	272	0.72	0.57	0.43
	Continental areas	46	0.30	0.61	0.39
		**p-value**	0.566 (ns)	0.943 (ns)	0.943 (ns)

Table S2. Summary of the best Generalized Linear Mixed Models (GLMM) adjusted using the sex expression (SE) and the phenotypic female-to-male sex ratio (PSR♀ and PSR♂) of each species as response variables. The best models were determined based on the Akaike Information Criterion (AICc). The direction of selected variables was assessed from the sign of standardized coefficients.

			**Coefficient**	**p-value**	**AIC**
**SE**	*E. intermedia*	Elevation	-0.00217	0.022	207.0
		Precipitation	-0.00009	<0.001	
		Temperature	-0.00333	0.001	
		**Null model**	-0.76660	0.081	228.1
	F. polysticta	Elevation	0.00026	0.867	124.6
		Precipitation	-0.00001	0.772	
		Temperature	-0.05717	0.125	
		**Null model**	1.55810	0.000	131.5
	F. teneriffae	Elevation	0.00075	0.257	288.4
		Precipitation	0.00001	0.744	
		Temperature	0.01930	0.013	
		**Null model**	-0.24410	0.644	430.0
	P. canariensis	Elevation	0.00341	<0.001	355.4
		Precipitation	0.00010	<0.001	
		Temperature	0.03795	<0.001	
		**Null model**	-0.27520	0.590	392.6
**PSR♀**	*E. intermedia*	Elevation	-0.00072	0.320	278.4
		Precipitation	0.00001	0.210	
		Temperature	0.00910	<0.001	
		**Null model**	0.05816	0.675	292.2
	F. polysticta	Elevation	-0.00029	0.816	141.8
		Precipitation	0.00002	0.366	
		Temperature	-0.03003	<0.001	
		**Null model**	1.33100	<0.001	142.6
	F. teneriffae	Elevation	-0.00036	0.237	362.9
		Precipitation	0.00003	0.023	

		Temperature	-0.00322	<0.001	
		**Null model**	0.02999	0.888	509.8
	P. canariensis	Elevation	-0.00005	0.924	390.0
		Precipitation	0.00003	0.016	
		Temperature	-0.01967	<0.001	
		**Null model**	0.39280	0.070	427.5
**PSR♂**	*E. intermedia*	Elevation	0.00105	0.436	188.6
		Precipitation	-0.00019	0.111	
		Temperature	0.01420	0.558	
		**Null model**	-1.41220	<0.001	201.4
	F. polysticta	Elevation	0.00094	0.596	107.6
		Precipitation	-0.00004	0.376	
		Temperature	0.01658	0.691	
		**Null model**	-1.96300	<0.001	107.0
	F. teneriffae	Elevation	0.00222	<0.001	269.8
		Precipitation	-0.00002	0.132	
		Temperature	0.01344	<0.001	
		**Null model**	-1.45280	<0.001	394.4
	P. canariensis	Elevation	0.00104	0.210	340.6
		Precipitation	-0.00005	0.403	
		Temperature	0.02589	0.133	
		**Null model**	-1.73057	<0.001	366.8

Table S3. Location of the studie species (Lon.: longitude; Lat.: latitude) for each sample (ID). Source (perviously collected herbarium specimen or fieldwork for this study), the sampling date (Date), elevation m (a.s.l.) (Elev.), host tree (NA = not collected from a tree or missing information on the herbarium label; Species unknown = collected from a tree, but the tree could not be identified), sex expression (SE), phenotypic sex ratio (PSR ♀: females; and ♂: males), and the frequency of sporophytes (FSP) are also provided.

Source	ID	Geographic region	Date	Lon.	Lat.	Species	Elev.	Host Tree	SE	PSR♀	PSR♂	FSP
AZU Herbarium	5667	Azores	28/07/2000	-25.31833	37.78120	*E. intermedia*	420	*NA*	1.000	1.000	0.000	0.000
AZU Herbarium	RG-2136	Azores	02/07/1992	-28.29727	38.44962	*E. intermedia*	560	*Laurus azorica*	1.000	0.000	1.000	0.000
AZU Herbarium	RG2136a	Azores	02/07/1992	-28.29727	38.44962	*E. intermedia*	560	*Laurus azorica*	0.667	1.000	0.000	1.000
AZU Herbarium	RG-2254a	Azores	02/07/1992	-28.2721	38.45042	*E. intermedia*	575	*Laurus azorica*	1.000	1.000	0.000	0.000
AZU Herbarium	RG280a	Azores	06/07/1992	-27.2403	38.67113	*E. intermedia*	800	*Laurus azorica*	1.000	0.000	1.000	0.000
AZU Herbarium	RG280a	Azores	06/07/1992	-27.2403	38.67113	*E. intermedia*	800	*Laurus azorica*	1.000	0.000	1.000	0.000
AZU Herbarium	RG280a	Azores	06/07/1992	-27.2403	38.67113	*E. intermedia*	800	*Laurus azorica*	1.000	0.000	1.000	0.000
AZU Herbarium	RG280a	Azores	06/07/1992	-27.2403	38.67113	*E. intermedia*	800	*Laurus azorica*	1.000	0.000	1.000	0.000
AZU Herbarium	RG-1915	Azores	12/06/1992	-27.2152	38.72756	*E. intermedia*	540	*NA*	1.000	0.000	1.000	0.000
AZU Herbarium	RG_20090625_Q13_06	Azores	25/06/2009	-27.2152	38.72756	*E. intermedia*	585	*NA*	1.000	0.000	1.000	0.000
AZU Herbarium	RG_20090625_Q03_03	Azores	25/06/2009	-27.2152	38.72756	*E. intermedia*	585	*NA*	1.000	1.000	0.000	0.000
AZU Herbarium	RG-2613	Azores	18/07/1992	-27.2152	38.72756	*E. intermedia*	570	*Laurus azorica*	0.167	0.000	1.000	0.000
AZU Herbarium	Barcelos, P.	Azores	13/08/1997	-27.193	38.73716	*E. intermedia*	620	*NA*	1.000	1.000	0.000	0.000
LISU Herbarium	162736	Azores	11/07/1992	-28.2541	38.4815	*E. intermedia*	500	*Ilex perado*	0.000	0.000	0.000	0.000
LISU Herbarium	237779	Azores	10/09/2007	-28.2133	38.4616	*E. intermedia*	300	*Species unknown*	0.333	0.000	1.000	0.000
LISU Herbarium	256613	Azores	04/05/1937	-28.7148	38.585	*E. intermedia*		*NA*	0.000	0.000	0.000	0.000
LISU Herbarium	264672	Azores		-27.9603	38.5989	*E. intermedia*	200	*Species unknown*	0.143	1.000	0.000	1.000
LISU Herbarium	256647	Azores	13/05/1937	-28.0296	38.6579	*E. intermedia*		*NA*	0.333	1.000	0.000	0.000
LISU Herbarium	270497	Azores	01/05/1937	-31.1703	39.4455	*E. intermedia*	500	*NA*	0.875	1.000	0.000	0.571
LISU Herbarium	225295	Azores	01/08/1997	-27.8109	38.5395	*E. intermedia*		*Species unknown*	0.000	0.000	0.000	0.000

LISU Herbarium	264673	Azores		-28.6758	38.5665	*E. intermedia*	420	*Platanus orientalis*	0.500	1.000	0.000	0.000
LISU Herbarium	225292	Azores	01/07/1997	-27.9283	38.5835	*E. intermedia*		*NA*	0.000	0.000	0.000	0.000
LISU Herbarium	225294	Azores	01/08/1997	-27.9532	38.6016	*E. intermedia*		*Species unknown*	0.000	0.000	0.000	0.000
LISU Herbarium	225296	Azores	21/06/1999	-27.9429	38.6044	*E. intermedia*	600	*NA*	0.600	0.000	1.000	0.000
LISU Herbarium	225293	Azores	01/08/1997	-27.9804	38.6432	*E. intermedia*		*Species unknown*	0.000	0.000	0.000	0.000
LISU Herbarium	256644	Azores	19/08/1938	-28.1268	38.687	*E. intermedia*		*NA*	1.000	1.000	0.000	0.250
LISU Herbarium	195990	Azores	03/08/2003	-31.1662	39.4612	*E. intermedia*	250	*Species unknown*	0.222	1.000	0.000	0.000
TFC Herbarium	4517	Canary Islands	25/03/1985	-16.8236	28.338	*E. intermedia*	760	*NA*	0.500	1.000	0.000	0.000
TFC Herbarium	4518	Canary Islands	09/02/1999	-16.8236	28.338	*E. intermedia*	760	*NA*	0.667	1.000	0.000	0.000
TFC Herbarium	4519	Canary Islands	25/03/1985	-16.8236	28.338	*E. intermedia*	760	*NA*	0.571	1.000	0.000	0.000
TFC Herbarium	4979	Canary Islands	27/04/1987	-16.8236	28.338	*E. intermedia*	760	*NA*	0.000	0.000	0.000	0.000
TFC Herbarium	1648	Canary Islands	30/04/1986	-16.8236	28.338	*E. intermedia*	760	*NA*	1.000	1.000	0.000	0.750
TFC Herbarium	1624	Canary Islands	30/04/1986	-16.8236	28.338	*E. intermedia*	760	*Viburnum tinus*	1.000	0.800	0.200	0.000
TFC Herbarium	1649	Canary Islands	17/03/1986	-16.8236	28.338	*E. intermedia*	760	*NA*	1.000	1.000	0.000	0.333
TFC Herbarium	1647	Canary Islands	30/04/1986	-16.8236	28.338	*E. intermedia*	760	*Viburnum tinus*	1.000	1.000	0.000	0.800
TFC Herbarium	1646	Canary Islands	17/03/1986	-16.8236	28.338	*E. intermedia*	760	*Laurus azorica*	1.000	1.000	0.000	0.500
TFC Herbarium	1657	Canary Islands	15/12/1986	-16.8236	28.338	*E. intermedia*	760	*NA*	0.000	0.000	0.000	0.000
TFC Herbarium	1657	Canary Islands	15/12/1986	-16.8236	28.338	*E. intermedia*	760	*Piconia excelsa*	0.000	0.000	0.000	0.000
TFC Herbarium	1648	Canary Islands	17/03/1986	-16.8236	28.338	*E. intermedia*	760	*Species unknown*	1.000	1.000	0.000	0.444
TFC Herbarium	1644	Canary Islands	17/03/1986	-16.8236	28.338	*E. intermedia*	760	*Species unknown*	1.000	1.000	0.000	0.167
TFC Herbarium	252	Canary Islands	25/05/1982	-16.2957	28.5231	*E. intermedia*	720	*NA*	1.000	0.000	1.000	0.000
TFC Herbarium	251	Canary Islands	25/05/1982	-16.2957	28.5231	*E. intermedia*	720	*NA*	0.400	1.000	0.000	0.000
TFC Herbarium	17530	Canary Islands	22/03/2009	-16.2796	28.5316	*E. intermedia*	950	*NA*	0.500	1.000	0.000	0.000
TFC Herbarium	9578	Canary Islands	15/02/1999	-16.2642	28.5334	*E. intermedia*	987	*Species unknown*	0.333	0.000	1.000	0.000
TFC Herbarium	9577	Canary Islands	24/04/1993	-16.2642	28.5334	*E. intermedia*	987	*Species unknown*	0.000	0.000	0.000	0.000
TFC Herbarium	9579	Canary Islands	11/05/1992	-16.2642	28.5334	*E. intermedia*	987	*Species unknown*	0.750	1.000	0.000	0.000

TFC Herbarium	10940	Canary Islands	02/02/1999	-16.2642	28.5334	*E. intermedia*	920	*NA*	0.250	1.000	0.000	0.000
TFC Herbarium	17533	Canary Islands	29/04/2009	-16.2291	28.543	*E. intermedia*	814	*Species unknown*	1.000	1.000	0.000	1.000
TFC Herbarium	250	Canary Islands	25/05/1982	-16.2068	28.549	*E. intermedia*	670	*NA*	1.000	1.000	0.000	0.000
TFC Herbarium	937	Canary Islands	23/01/1982	-16.1859	28.5527	*E. intermedia*	700	*NA*	0.500	0.000	1.000	0.000
TFC Herbarium	936	Canary Islands	23/06/1982			*E. intermedia*		*NA*	1.000	0.000	1.000	0.000
Fieldwork	1.1	Canary Islands	19/10/2022	-16.2678	28.5344	*E. intermedia*	980	*Laurus novocanariensis*	0.800	0.250	0.750	0.000
Fieldwork	1.4	Canary Islands	19/10/2022	-16.2678	28.5344	*E. intermedia*	980	*Laurus novocanariensis*	1.000	0.889	0.111	0.000
Fieldwork	1.5	Canary Islands	19/10/2022	-16.2678	28.5344	*E. intermedia*	980	*Laurus novocanariensis*	0.667	0.000	1.000	0.000
Fieldwork	1.6	Canary Islands	19/10/2022	-16.2678	28.5344	*E. intermedia*	980	*Laurus novocanariensis*	0.000	0.000	0.000	0.000
Fieldwork	1.8	Canary Islands	19/10/2022	-16.2678	28.5344	*E. intermedia*	980	*Laurus novocanariensis*	0.333	1.000	0.000	0.000
Fieldwork	10.1	Canary Islands	21/10/2022	-16.8161	28.3225	*E. intermedia*	998	*Persea indica*	0.000	0.000	0.000	0.000
Fieldwork	10.3	Canary Islands	21/10/2022	-16.8161	28.3225	*E. intermedia*	998	*Laurus novocanariensis*	0.000	0.000	0.000	0.000
Fieldwork	10.5	Canary Islands	21/10/2022	-16.8161	28.3225	*E. intermedia*	998	*Laurus novocanariensis*	0.000	0.000	0.000	0.000
Fieldwork	10.6	Canary Islands	21/10/2022	-16.8161	28.3225	*E. intermedia*	998	*Laurus novocanariensis*	0.000	0.000	0.000	0.000
Fieldwork	10.8	Canary Islands	21/10/2022	-16.8161	28.3225	*E. intermedia*	998	*Laurus novocanariensis*	0.167	1.000	0.000	0.000
Fieldwork	11.10	Canary Islands	21/10/2022	-16.8222	28.3253	*E. intermedia*	964	*Laurus novocanariensis*	0.000	0.000	0.000	0.000
Fieldwork	11.11	Canary Islands	21/10/2022	-16.8222	28.3253	*E. intermedia*	964	*Laurus novocanariensis*	0.000	0.000	0.000	0.000
Fieldwork	11.12	Canary Islands	21/10/2022	-16.8222	28.3253	*E. intermedia*	964	*Laurus novocanariensis*	0.762	1.000	0.000	0.000
Fieldwork	11.13	Canary Islands	21/10/2022	-16.8222	28.3253	*E. intermedia*	964	*Laurus novocanariensis*	0.000	0.000	0.000	0.000
Fieldwork	11.9	Canary Islands	21/10/2022	-16.8222	28.3253	*E. intermedia*	964	*Laurus novocanariensis*	0.000	0.000	0.000	0.000
Fieldwork	13.1	Canary Islands	18/10/2022	-16.3064	28.5369	*E. intermedia*	809	*Ilex canariensis*	0.000	0.000	0.000	0.000
Fieldwork	13.10	Canary Islands	18/10/2022	-16.3064	28.5369	*E. intermedia*	809	*Laurus novocanariensis*	0.500	1.000	0.000	0.000

Fieldwork	13.11	Canary Islands	18/10/2022	-16.3064	28.5369	*E. intermedia*	809	*Laurus novocanariensis*	0.375	0.000	1.000	0.000
Fieldwork	13.12	Canary Islands	18/10/2022	-16.3064	28.5369	*E. intermedia*	809	*Laurus novocanariensis*	1.000	1.000	0.000	0.250
Fieldwork	13.6	Canary Islands	18/10/2022	-16.3064	28.5369	*E. intermedia*	809	*Prunus lusitanica*	0.714	1.000	0.000	0.000
Fieldwork	2.1	Canary Islands	17/10/2022	-16.1697	28.5594	*E. intermedia*	783	*Ilex canariensis*	0.200	1.000	0.000	0.000
Fieldwork	2.2	Canary Islands	17/10/2022	-16.1697	28.5594	*E. intermedia*	783	*Ilex canariensis*	0.250	1.000	0.000	0.000
Fieldwork	2.3	Canary Islands	17/10/2022	-16.1697	28.5594	*E. intermedia*	783	*Laurus novocanariensis*	0.111	1.000	0.000	0.000
Fieldwork	2.4	Canary Islands	17/10/2022	-16.1697	28.5594	*E. intermedia*	783	*Laurus novocanariensis*	1.000	0.833	0.167	0.000
Fieldwork	2.5	Canary Islands	17/10/2022	-16.1697	28.5594	*E. intermedia*	783	*Persea indica*	0.857	0.833	0.167	0.000
Fieldwork	6.1	Canary Islands	19/10/2022	-16.2278	28.5425	*E. intermedia*	860	*Ilex canariensis*	0.000	0.000	0.000	0.000
Fieldwork	6.10	Canary Islands	19/10/2022	-16.2278	28.5425	*E. intermedia*	860	*Laurus novocanariensis*	0.200	0.000	1.000	0.000
Fieldwork	6.11	Canary Islands	19/10/2022	-16.2278	28.5425	*E. intermedia*	860	*Viburnum rugosum*	0.125	1.000	0.000	0.000
Fieldwork	6.12	Canary Islands	19/10/2022	-16.2278	28.5425	*E. intermedia*	860	*Laurus novocanariensis*	1.000	1.000	0.000	0.000
Fieldwork	6.13	Canary Islands	19/10/2022	-16.2278	28.5425	*E. intermedia*	860	*Ilex canariensis*	0.059	1.000	0.000	0.000
Fieldwork	7.1	Canary Islands	17/10/2022	-16.2856	28.5308	*E. intermedia*	932	*Laurus novocanariensis*	0.000	0.000	0.000	0.000
Fieldwork	7.2	Canary Islands	17/10/2022	-16.2856	28.5308	*E. intermedia*	932	*Laurus novocanariensis*	0.000	0.000	0.000	0.000
Fieldwork	7.3	Canary Islands	17/10/2022	-16.2856	28.5308	*E. intermedia*	932	*Laurus novocanariensis*	0.545	1.000	0.000	0.167
Fieldwork	7.4	Canary Islands	17/10/2022	-16.2856	28.5308	*E. intermedia*	932	*Laurus novocanariensis*	1.000	1.000	0.000	0.000
Fieldwork	7.5	Canary Islands	17/10/2022	-16.2856	28.5308	*E. intermedia*	932	*Laurus novocanariensis*	0.167	1.000	0.000	0.000
Fieldwork	8.11	Canary Islands	19/10/2022	-16.5894	28.3486	*E. intermedia*	1211	*Viburnum rugosum*	0.368	0.000	1.000	0.000
Fieldwork	8.13	Canary Islands	19/10/2022	-16.5894	28.3486	*E. intermedia*	1211	*Laurus novocanariensis*	0.000	0.000	0.000	0.000
Fieldwork	8.14	Canary Islands	19/10/2022	-16.5894	28.3486	*E. intermedia*	1211	*Ilex canariensis*	0.050	0.000	1.000	0.000
Fieldwork	8.15	Canary Islands	19/10/2022	-16.5894	28.3486	*E. intermedia*	1211	*Laurus novocanariensis*	0.000	0.000	0.000	0.000

Fieldwork	8.8	Canary Islands	19/10/2022	-16.5894	28.3486	*E. intermedia*	1211	*Laurus novocanariensis*	0.000	0.000	0.000	0.000
LISU Herbarium	162250	Madeira	28/06/1988	-17.1361	32.8078	*E. intermedia*	700	*Species unknown*	0.308	1.000	0.000	0.250
LISU Herbarium	260883	Madeira	17/11/2005	-16.9209	32.7301	*E. intermedia*	960	*Laurus novocanariensis*	0.222	1.000	0.000	0.000
LISU Herbarium	260884	Madeira	17/11/2005	-16.9209	32.7301	*E. intermedia*	960	*Heberdenia excelsa*	0.700	0.571	0.429	0.000
LISU Herbarium	149157	Madeira	12/08/1938	-17.049	32.7302	*E. intermedia*		*NA*	0.500	1.000	0.000	1.000
LISU Herbarium	261023	Madeira	21/07/2005	-16.9102	32.7303	*E. intermedia*	900	*Laurus novocanariensis*	0.200	1.000	0.000	0.000
LISU Herbarium	162256	Madeira	09/05/1982	-16.8889	32.7306	*E. intermedia*	800	*Species unknown*	0.222	1.000	0.000	0.000
LISU Herbarium	162254	Madeira	10/05/1990	-16.8676	32.7309	*E. intermedia*	750	*Species unknown*	0.357	1.000	0.000	0.400
LISU Herbarium	162251	Madeira	10/05/1990	-16.8676	32.7309	*E. intermedia*	750	*NA*	0.071	0.000	1.000	0.000
LISU Herbarium	162257	Madeira	05/04/1988	-17.0599	32.7372	*E. intermedia*		*Species unknown*	0.125	0.000	1.000	0.000
LISU Herbarium	261202	Madeira	15/11/2005	-16.9104	32.7393	*E. intermedia*	660	*Ilex canariensis*	0.000	0.000	0.000	0.000
LISU Herbarium	261212	Madeira	16/11/2005	-16.9104	32.7393	*E. intermedia*	850	*Laurus novocanariensis*	0.800	1.000	0.000	0.000
LISU Herbarium	251167	Madeira	09/08/2001	-17.0493	32.7461	*E. intermedia*	1000	*NA*	0.143	1.000	0.000	0.000
LISU Herbarium	254370	Madeira	29/07/2003	-17.1348	32.7537	*E. intermedia*	1100	*NA*	0.000	0.000	0.000	0.000
LISU Herbarium	251176	Madeira	09/08/2001	-17.0495	32.7551	*E. intermedia*	1000	*NA*	0.000	0.000	0.000	0.000
LISU Herbarium	162253	Madeira	11/05/1982	-17.0175	32.7557	*E. intermedia*	600	*NA*	0.167	1.000	0.000	0.000
LISU Herbarium	162248	Madeira	11/05/1982	-17.0175	32.7557	*E. intermedia*	600	*NA*	0.500	1.000	0.000	0.000
LISU Herbarium	261197	Madeira	27/07/2005	-17.0068	32.7558	*E. intermedia*	600	*Ocotea foetens*	0.000	0.000	0.000	0.000
LISU Herbarium	149155	Madeira	24/04/1984	-17.0201	32.7595	*E. intermedia*		*NA*	0.143	1.000	0.000	0.000
LISU Herbarium	162255	Madeira	11/10/1990	-17.0604	32.764	*E. intermedia*	1050	*NA*	0.400	1.000	0.000	0.500
LISU Herbarium	162260	Madeira	30/06/1982	-17.0604	32.764	*E. intermedia*	1000	*Species unknown*	0.500	1.000	0.000	0.500
LISU Herbarium	149154	Madeira	30/06/1982	-17.0604	32.764	*E. intermedia*	1000	*Species unknown*	0.294	1.000	0.000	0.400
LISU Herbarium	261005	Madeira	20/07/2005	-16.9323	32.766	*E. intermedia*	1300	*Clethra arborea*	0.000	0.000	0.000	0.000
LISU Herbarium	149156	Madeira	02/08/1939	-16.9432	32.7749	*E. intermedia*		*NA*	0.500	1.000	0.000	0.000
LISU Herbarium	149159	Madeira	02/08/1929	-16.9432	32.7749	*E. intermedia*		*NA*	0.200	1.000	0.000	1.000

LISU Herbarium	261077	Madeira	03/08/2004	-16.9219	32.7752	*E. intermedia*	890	*Laurus novocanariensis*	0.333	1.000	0.000	0.000
LISU Herbarium	261090	Madeira	05/08/2004	-17.0928	32.7815	*E. intermedia*	1000	*Laurus novocanariensis*	0.077	1.000	0.000	0.000
LISU Herbarium	261159	Madeira	03/08/2006	-16.9968	32.783	*E. intermedia*	950	*Myrica Faya*	0.000	0.000	0.000	0.000
LISU Herbarium	261184	Madeira	22/07/2005	-17.0823	32.7907	*E. intermedia*	850	*Laurus novocanariensis*	0.500	0.000	1.000	0.000
LISU Herbarium	260919	Madeira	02/08/2007	-16.997	32.7921	*E. intermedia*	800	*Rhamnus glandulosa*	0.000	0.000	0.000	0.000
LISU Herbarium	261066	Madeira	28/07/2004	-17.1359	32.7988	*E. intermedia*	1200	*Laurus novocanariensis*	0.467	1.000	0.000	0.286
LISU Herbarium	260908	Madeira	24/11/2005	-17.1252	32.799	*E. intermedia*	1135	*Laurus novocanariensis*	0.167	1.000	0.000	1.000
LISU Herbarium	162247	Madeira	28/06/1988	-17.1252	32.799	*E. intermedia*	1250	*Species unknown*	0.000	0.000	0.000	0.000
Fieldwork	MD1.1	Madeira	29/11/2022	-17.1405	32.8101	*E. intermedia*	1398	*Laurus novocanariensis*	0.333	1.000	0.000	0.000
Fieldwork	MD1.2	Madeira	29/11/2022	-17.1405	32.8101	*E. intermedia*	1398	*Ocotea foetens*	0.000	0.000	0.000	0.000
Fieldwork	MD1.3	Madeira	29/11/2022	-17.1405	32.8101	*E. intermedia*	1398	*Ocotea foetens*	0.000	0.000	0.000	0.000
Fieldwork	MD1.4	Madeira	29/11/2022	-17.1405	32.8101	*E. intermedia*	1398	*Ocotea foetens*	0.333	1.000	0.000	0.333
Fieldwork	MD1.5	Madeira	29/11/2022	-17.1405	32.8101	*E. intermedia*	1398	*Laurus novocanariensis*	0.000	0.000	0.000	0.000
Fieldwork	MD10.1	Madeira	30/11/2022	-16.9392	32.8064	*E. intermedia*	635	*Persea indica*	0.444	0.250	0.750	0.000
Fieldwork	MD10.2	Madeira	30/11/2022	-16.9392	32.8064	*E. intermedia*	635	*Persea indica*	0.400	0.000	1.000	0.000
Fieldwork	MD10.3	Madeira	30/11/2022	-16.9392	32.8064	*E. intermedia*	635	*Persea indica*	0.000	0.000	0.000	0.000
Fieldwork	MD10.4	Madeira	30/11/2022	-16.9392	32.8064	*E. intermedia*	635	*Laurus novocanariensis*	0.364	1.000	0.000	0.000
Fieldwork	MD10.5	Madeira	30/11/2022	-16.9392	32.8064	*E. intermedia*	635	*Laurus novocanariensis*	0.000	0.000	0.000	0.000
Fieldwork	MD11.1	Madeira	30/11/2022	-16.9372	32.7939	*E. intermedia*	640	*Ocotea foetens*	0.333	1.000	0.000	0.333
Fieldwork	MD11.2	Madeira	30/11/2022	-16.9372	32.7939	*E. intermedia*	640	*Ocotea foetens*	0.000	0.000	0.000	0.000
Fieldwork	MD11.3	Madeira	30/11/2022	-16.9372	32.7939	*E. intermedia*	640	*Ocotea foetens*	0.250	0.500	0.500	1.000
Fieldwork	MD11.4	Madeira	30/11/2022	-16.9372	32.7939	*E. intermedia*	640	*Ocotea foetens*	1.000	0.786	0.214	0.455
Fieldwork	MD11.5	Madeira	30/11/2022	-16.9372	32.7939	*E. intermedia*	640	*Laurus novocanariensis*	0.100	1.000	0.000	0.000
Fieldwork	MD12.1	Madeira	29/11/2022	-17.1142	32.7911	*E. intermedia*	584	*Laurus novocanariensis*	0.368	0.857	0.143	0.333

Fieldwork	MD12.3	Madeira	29/11/2022	-17.1142	32.7911	*E. intermedia*	584	*Ocotea foetens*	0.167	1.000	0.000	0.000
Fieldwork	MD12.4	Madeira	29/11/2022	-17.1142	32.7911	*E. intermedia*	584	*Persea indica*	0.000	0.000	0.000	0.000
Fieldwork	MD12.6	Madeira	29/11/2022	-17.1142	32.7911	*E. intermedia*	584	*Ocotea foetens*	0.000	0.000	0.000	0.000
Fieldwork	MD12.7	Madeira	29/11/2022	-17.1142	32.7911	*E. intermedia*	584	*Laurus novocanariensis*	0.111	0.000	1.000	0.000
Fieldwork	MD13.1	Madeira	29/11/2022	-17.1136	32.7867	*E. intermedia*	721	*Ocotea foetens*	0.200	1.000	0.000	0.500
Fieldwork	MD13.3	Madeira	29/11/2022	-17.1136	32.7867	*E. intermedia*	721	*Ocotea foetens*	0.000	0.000	0.000	0.000
Fieldwork	MD13.4	Madeira	29/11/2022	-17.1136	32.7867	*E. intermedia*	721	*Ocotea foetens*	0.000	0.000	0.000	0.000
Fieldwork	MD13.5	Madeira	29/11/2022	-17.1136	32.7867	*E. intermedia*	721	*Ocotea foetens*	0.276	1.000	0.000	0.375
Fieldwork	MD13.8	Madeira	29/11/2022	-17.1136	32.7867	*E. intermedia*	721	*Ocotea foetens*	0.000	0.000	0.000	0.000
Fieldwork	MD14.1	Madeira	28/11/2022	-16.9075	32.7836	*E. intermedia*	899	*Laurus novocanariensis*	1.000	1.000	0.000	0.313
Fieldwork	MD14.2	Madeira	28/11/2022	-16.9075	32.7836	*E. intermedia*	899	*Laurus novocanariensis*	0.333	1.000	0.000	0.000
Fieldwork	MD14.3	Madeira	28/11/2022	-16.9075	32.7836	*E. intermedia*	899	*Laurus novocanariensis*	0.000	0.000	0.000	0.000
Fieldwork	MD14.5	Madeira	28/11/2022	-16.9075	32.7836	*E. intermedia*	899	*Persea indica*	0.000	0.000	0.000	0.000
Fieldwork	MD14.7	Madeira	28/11/2022	-16.9075	32.7836	*E. intermedia*	899	*Laurus novocanariensis*	0.200	1.000	0.000	0.500
Fieldwork	MD16.1	Madeira	28/11/2022	-16.925	32.7847	*E. intermedia*	933	*Laurus novocanariensis*	0.250	1.000	0.000	0.250
Fieldwork	MD16.2	Madeira	28/11/2022	-16.925	32.7847	*E. intermedia*	933	*Laurus novocanariensis*	0.353	0.250	0.750	0.000
Fieldwork	MD16.3	Madeira	28/11/2022	-16.925	32.7847	*E. intermedia*	933	*Laurus novocanariensis*	0.286	1.000	0.000	0.000
Fieldwork	MD16.5	Madeira	28/11/2022	-16.925	32.7847	*E. intermedia*	933	*Laurus novocanariensis*	0.188	0.333	0.667	0.000
Fieldwork	MD16.6	Madeira	28/11/2022	-16.925	32.7847	*E. intermedia*	933	*Laurus novocanariensis*	0.300	0.833	0.167	0.000
Fieldwork	MD17.1	Madeira	30/11/2022	-16.9769	32.77	*E. intermedia*	1074	*Ocotea foetens*	1.000	1.000	0.000	0.050
Fieldwork	MD17.10	Madeira	30/11/2022	-16.9769	32.77	*E. intermedia*	1074	*Ocotea foetens*	0.600	0.000	1.000	0.000
Fieldwork	MD17.7	Madeira	30/11/2022	-16.9769	32.77	*E. intermedia*	1074	*Ocotea foetens*	0.000	0.000	0.000	0.000
Fieldwork	MD17.8	Madeira	30/11/2022	-16.9769	32.77	*E. intermedia*	1074	*Ocotea foetens*	0.000	0.000	0.000	0.000
Fieldwork	MD17.9	Madeira	30/11/2022	-16.9769	32.77	*E. intermedia*	1074	*Ocotea foetens*	0.714	1.000	0.000	0.000

Fieldwork	MD18.1	Madeira	30/11/2022	-16.9797	32.7775	*E. intermedia*	670	*Laurus novocanariensis*	0.417	0.400	0.600	0.500
Fieldwork	MD18.2	Madeira	30/11/2022	-16.9797	32.7775	*E. intermedia*	670	*Laurus novocanariensis*	0.250	1.000	0.000	0.000
Fieldwork	MD18.3	Madeira	30/11/2022	-16.9797	32.7775	*E. intermedia*	670	*Laurus novocanariensis*	0.091	0.500	0.500	0.000
Fieldwork	MD18.4	Madeira	30/11/2022	-16.9797	32.7775	*E. intermedia*	670	*Laurus novocanariensis*	0.188	0.667	0.333	0.500
Fieldwork	MD18.6	Madeira	30/11/2022	-16.9797	32.7775	*E. intermedia*	670	*Laurus novocanariensis*	0.455	0.300	0.700	0.000
Fieldwork	MD2.2	Madeira	29/11/2022	-17.1578	32.8253	*E. intermedia*	831	*Laurus novocanariensis*	0.250	1.000	0.000	0.500
Fieldwork	MD2.3	Madeira	29/11/2022	-17.1578	32.8253	*E. intermedia*	831	*Laurus novocanariensis*	0.000	0.000	0.000	0.000
Fieldwork	MD2.6	Madeira	29/11/2022	-17.1578	32.8253	*E. intermedia*	831	*Laurus novocanariensis*	0.111	0.000	1.000	0.000
Fieldwork	MD2.7	Madeira	29/11/2022	-17.1578	32.8253	*E. intermedia*	831	*Laurus novocanariensis*	0.048	1.000	0.000	0.000
Fieldwork	MD2.9	Madeira	29/11/2022	-17.1578	32.8253	*E. intermedia*	831	*Laurus novocanariensis*	0.231	1.000	0.000	0.000
Fieldwork	MD3.2	Madeira	28/11/2022	-16.8853	32.7347	*E. intermedia*	879	*Laurus novocanariensis*	0.300	1.000	0.000	0.333
Fieldwork	MD3.3	Madeira	28/11/2022	-16.8853	32.7347	*E. intermedia*	879	*Laurus novocanariensis*	0.000	0.000	0.000	0.000
Fieldwork	MD3.4	Madeira	28/11/2022	-16.8853	32.7347	*E. intermedia*	879	*Laurus novocanariensis*	0.222	1.000	0.000	0.000
Fieldwork	MD3.6	Madeira	28/11/2022	-16.8853	32.7347	*E. intermedia*	879	*Laurus novocanariensis*	0.200	0.000	1.000	0.000
Fieldwork	MD3.7	Madeira	28/11/2022	-16.8853	32.7347	*E. intermedia*	879	*Laurus novocanariensis*	0.333	0.000	1.000	0.000
Fieldwork	MD4.1	Madeira	28/11/2022	-16.8892	32.7408	*E. intermedia*	812	*Laurus novocanariensis*	0.118	1.000	0.000	0.000
Fieldwork	MD4.3	Madeira	28/11/2022	-16.8892	32.7408	*E. intermedia*	812	*Laurus novocanariensis*	0.000	0.000	0.000	0.000
Fieldwork	MD4.4	Madeira	28/11/2022	-16.8892	32.7408	*E. intermedia*	812	*Laurus novocanariensis*	0.000	0.000	0.000	0.000
Fieldwork	MD4.5	Madeira	28/11/2022	-16.8892	32.7408	*E. intermedia*	812	*Laurus novocanariensis*	0.000	0.000	0.000	0.000
Fieldwork	MD4.6	Madeira	28/11/2022	-16.8892	32.7408	*E. intermedia*	812	*Persea indica*	0.206	0.429	0.571	0.000
Fieldwork	MD5.1	Madeira	02/12/2022	-16.9151	32.7421	*E. intermedia*	919	*Laurus novocanariensis*	0.000	0.000	0.000	0.000

Fieldwork	MD5.2	Madeira	02/12/2022	-16.9151	32.7421	*E. intermedia*	919	*Laurus novocanariensis*	0.286	1.000	0.000	0.125
Fieldwork	MD5.3	Madeira	02/12/2022	-16.9151	32.7421	*E. intermedia*	919	*Laurus novocanariensis*	0.167	1.000	0.000	0.000
Fieldwork	MD5.4	Madeira	02/12/2022	-16.9151	32.7421	*E. intermedia*	919	*Laurus novocanariensis*	0.211	1.000	0.000	0.000
Fieldwork	MD5.5	Madeira	02/12/2022	-16.9151	32.7421	*E. intermedia*	919	*Laurus novocanariensis*	0.107	0.667	0.333	0.500
Fieldwork	MD6.1	Madeira	02/12/2022	-16.9102	32.7436	*E. intermedia*	730	*Laurus novocanariensis*	0.222	0.000	1.000	0.000
Fieldwork	MD6.2	Madeira	02/12/2022	-16.9102	32.7436	*E. intermedia*	730	*Laurus novocanariensis*	0.947	1.000	0.000	0.111
Fieldwork	MD6.3	Madeira	02/12/2022	-16.9102	32.7436	*E. intermedia*	730	*Laurus novocanariensis*	0.375	1.000	0.000	0.000
Fieldwork	MD6.7	Madeira	02/12/2022	-16.9102	32.7436	*E. intermedia*	730	*Laurus novocanariensis*	0.000	0.000	0.000	0.000
Fieldwork	MD6.9	Madeira	02/12/2022	-16.9102	32.7436	*E. intermedia*	730	*Laurus novocanariensis*	0.000	0.000	0.000	0.000
Fieldwork	MD7.1	Madeira	02/12/2022	-16.9061	32.7453	*E. intermedia*	790	*Laurus novocanariensis*	0.250	1.000	0.000	1.000
Fieldwork	MD7.2	Madeira	02/12/2022	-16.9061	32.7453	*E. intermedia*	790	*Laurus novocanariensis*	0.000	0.000	0.000	0.000
Fieldwork	MD7.3	Madeira	02/12/2022	-16.9061	32.7453	*E. intermedia*	790	*Laurus novocanariensis*	0.333	1.000	0.000	0.333
Fieldwork	MD7.4	Madeira	02/12/2022	-16.9061	32.7453	*E. intermedia*	790	*Ocotea foetens*	0.160	0.750	0.250	0.667
Fieldwork	MD7.5	Madeira	02/12/2022	-16.9061	32.7453	*E. intermedia*	790	*Laurus novocanariensis*	0.615	1.000	0.000	0.000
Fieldwork	MD8.1	Madeira	01/12/2022	-17.0186	32.7536	*E. intermedia*	1007	*Laurus novocanariensis*	0.176	1.000	0.000	0.667
Fieldwork	MD8.12	Madeira	01/12/2022	-17.0186	32.7536	*E. intermedia*	1007	*Quercus robur*	0.267	0.250	0.750	1.000
Fieldwork	MD8.6	Madeira	01/12/2022	-17.0186	32.7536	*E. intermedia*	1007	*Laurus novocanariensis*	0.250	1.000	0.000	1.000
Fieldwork	MD8.7	Madeira	01/12/2022	-17.0186	32.7536	*E. intermedia*	1007	*Hesperocyparis macrocarpa*	1.000	1.000	0.000	0.273
Fieldwork	MD8.8	Madeira	01/12/2022	-17.0186	32.7536	*E. intermedia*	1007	*Hesperocyparis macrocarpa*	0.545	1.000	0.000	0.167
Fieldwork	MD9.1	Madeira	01/12/2022	-17.0153	32.7603	*E. intermedia*	810	*Laurus novocanariensis*	0.200	1.000	0.000	0.000
Fieldwork	MD9.10	Madeira	01/12/2022	-17.0153	32.7603	*E. intermedia*	810	*Laurus novocanariensis*	0.600	0.000	1.000	0.000

Fieldwork	MD9.4	Madeira	01/12/2022	-17.0153	32.7603	*E. intermedia*	810	*Laurus novocanariensis*	0.000	0.000	0.000	0.000
Fieldwork	MD9.7	Madeira	01/12/2022	-17.0153	32.7603	*E. intermedia*	810	*Laurus novocanariensis*	0.000	0.000	0.000	0.000
Fieldwork	MD9.9	Madeira	01/12/2022	-17.0153	32.7603	*E. intermedia*	810	*Laurus novocanariensis*	0.000	0.000	0.000	0.000
TFC Herbarium	1486	Canary Islands	30/10/1985	-16.8212	28.3386	*F. polysticta*	670	*Laurus azorica*	0.429	0.667	0.333	0.000
TFC Herbarium	1489	Canary Islands	30/10/1985	-16.8212	28.3386	*F. polysticta*	670	*Laurus azorica*	0.545	0.667	0.333	0.000
TFC Herbarium	1574	Canary Islands	30/04/1986	-16.8212	28.3386	*F. polysticta*	670	*Persea indica*	0.800	1.000	0.000	0.125
TFC Herbarium	1654	Canary Islands	15/12/1986	-16.8212	28.3386	*F. polysticta*	670	*Laurus azorica*	0.857	0.750	0.250	0.000
TFC Herbarium	1556	Canary Islands	30/04/1986	-16.8212	28.3386	*F. polysticta*	670	*Viburnum tinus* subsp. *zigidum*	0.765	0.846	0.154	0.091
TFC Herbarium	1618	Canary Islands	08/07/1983	-16.8212	28.3386	*F. polysticta*	670	*Persea indica*	0.524	0.455	0.545	0.200
TFC Herbarium	1606	Canary Islands	27/10/1986	-16.8212	28.3386	*F. polysticta*	670	*Laurus azorica*	0.895	0.824	0.176	0.071
TFC Herbarium	5237	Canary Islands	19/01/1987	-16.8212	28.3386	*F. polysticta*	670	*NA*	0.091	1.000	0.000	0.000
TFC Herbarium	5238	Canary Islands	15/02/1987	-16.8212	28.3386	*F. polysticta*	670	*NA*	0.000	0.000	0.000	0.000
TFC Herbarium	5247	Canary Islands		-16.8212	28.3386	*F. polysticta*	670	*NA*	0.333	0.000	1.000	0.000
TFC Herbarium	5248	Canary Islands	27/04/1987	-16.8212	28.3386	*F. polysticta*	670	*NA*	1.000	1.000	0.000	0.000
TFC Herbarium	2505	Canary Islands	01/06/1984	-16.4037	28.449	*F. polysticta*	1000	*Erica arborea*	1.000	0.462	0.538	0.167
TFC Herbarium	2962	Canary Islands	17/06/1985	-16.4037	28.449	*F. polysticta*	1000	*Ilex canariensis*	0.667	1.000	0.000	0.000
TFC Herbarium	2047	Canary Islands	05/11/1981	-16.4037	28.449	*F. polysticta*	1000	*Ilex canariensis*	0.053	1.000	0.000	0.000
TFC Herbarium	2348	Canary Islands	05/12/1983	-16.4051	28.4554	*F. polysticta*	920	*Laurus azorica*	0.867	0.462	0.538	0.000
TFC Herbarium	2693	Canary Islands	01/10/1984	-16.4051	28.4554	*F. polysticta*	920	*Persea indica*	0.769	1.000	0.000	0.000
TFC Herbarium	2571	Canary Islands	02/07/1984	-16.4021	28.4572	*F. polysticta*	860	*NA*	0.769	0.000	1.000	0.000
TFC Herbarium	5099	Canary Islands	15/05/1986	-16.4021	28.4572	*F. polysticta*	860	*Persea indica*	1.000	0.571	0.429	0.500
TFC Herbarium	2401	Canary Islands	07/05/1984	-16.4039	28.4584	*F. polysticta*	830	*Species unknown*	0.867	0.000	1.000	0.000
TFC Herbarium	3725	Canary Islands	09/04/1986	-16.4039	28.461	*F. polysticta*	800	*Myrica faya*	0.167	1.000	0.000	1.000
TFC Herbarium	17739	Canary Islands	22/04/2001	-16.2712	28.5305	*F. polysticta*	705	*Laurus novocanariensis*	0.429	0.667	0.333	0.000
TFC Herbarium	17737	Canary Islands	22/04/2001	-16.2712	28.5305	*F. polysticta*	705	*Laurus novocanariensis*	0.615	0.875	0.125	0.000
TFC Herbarium	17735	Canary Islands	22/04/2001	-16.2712	28.5305	*F. polysticta*	705	*Laurus novocanariensis*	0.667	0.500	0.500	0.000

TFC Herbarium	17879	Canary Islands	22/04/2001	-16.2712	28.5305	*F. polysticta*	705	*Laurus novocanariensis*	0.167	1.000	0.000	0.000
TFC Herbarium	17860	Canary Islands	22/04/2001	-16.2712	28.5305	*F. polysticta*	705	*Laurus novocanariensis*	0.167	0.500	0.500	0.000
TFC Herbarium	17882	Canary Islands	22/04/2001	-16.2712	28.5305	*F. polysticta*	705	*Laurus novocanariensis*	0.500	0.600	0.400	0.000
TFC Herbarium	17764	Canary Islands	22/04/2001	-16.2712	28.5305	*F. polysticta*	705	*Laurus novocanariensis*	0.625	0.400	0.600	0.000
TFC Herbarium	17761	Canary Islands	22/04/2001	-16.2712	28.5305	*F. polysticta*	705	*Laurus novocanariensis*	0.500	0.500	0.500	0.000
TFC Herbarium	17863	Canary Islands	22/04/2001	-16.2712	28.5305	*F. polysticta*	705	*Laurus novocanariensis*	0.429	0.333	0.667	0.000
TFC Herbarium	2163	Canary Islands	08/07/1983			*F. polysticta*		*Persea indica*	0.875	1.000	0.000	0.000
Fieldwork	10.3	Canary Islands	21/10/2022	-16.8161	28.3225	*F. polysticta*	998	*Laurus novocanariensis*	0.955	0.000	1.000	0.000
Fieldwork	10.4	Canary Islands	21/10/2022	-16.8161	28.3225	*F. polysticta*	998	*Laurus novocanariensis*	0.706	1.000	0.000	0.000
Fieldwork	10.5	Canary Islands	21/10/2022	-16.8161	28.3225	*F. polysticta*	998	*Laurus novocanariensis*	1.000	0.842	0.158	0.000
Fieldwork	10.6	Canary Islands	21/10/2022	-16.8161	28.3225	*F. polysticta*	998	*Laurus novocanariensis*	0.696	0.542	0.458	0.038
Fieldwork	10.8	Canary Islands	21/10/2022	-16.8161	28.3225	*F. polysticta*	998	*Laurus novocanariensis*	0.840	1.000	0.000	0.000
Fieldwork	11.1	Canary Islands	21/10/2022	-16.8222	28.3253	*F. polysticta*	964	*Laurus novocanariensis*	0.625	0.880	0.120	0.045
Fieldwork	11.14	Canary Islands	21/10/2022	-16.8222	28.3253	*F. polysticta*	964	*Ilex canariensis*	0.533	0.500	0.500	0.000
Fieldwork	11.3	Canary Islands	21/10/2022	-16.8222	28.3253	*F. polysticta*	964	*Laurus novocanariensis*	1.000	1.000	0.000	0.000
Fieldwork	11.5	Canary Islands	21/10/2022	-16.8222	28.3253	*F. polysticta*	964	*Laurus novocanariensis*	1.000	0.968	0.032	0.000
Fieldwork	11.7	Canary Islands	21/10/2022	-16.8222	28.3253	*F. polysticta*	964	*Laurus novocanariensis*	1.000	0.958	0.042	0.022
Fieldwork	12.1	Canary Islands	19/10/2022	-16.405	28.4542	*F. polysticta*	941	*Laurus novocanariensis*	0.971	0.606	0.394	0.050
Fieldwork	12.2	Canary Islands	19/10/2022	-16.405	28.4542	*F. polysticta*	941	*Laurus novocanariensis*	1.000	0.333	0.667	0.000
Fieldwork	12.3	Canary Islands	19/10/2022	-16.405	28.4542	*F. polysticta*	941	*Laurus novocanariensis*	1.000	0.936	0.064	0.000
Fieldwork	12.4	Canary Islands	19/10/2022	-16.405	28.4542	*F. polysticta*	941	*Laurus novocanariensis*	1.000	1.000	0.000	0.000

Fieldwork	12.6	Canary Islands	19/10/2022	-16.405	28.4542	*F. polysticta*	941	*Laurus novocanariensis*	1.000	1.000	0.000	0.000
Fieldwork	9.1	Canary Islands	19/10/2022	-16.4025	28.4581	*F. polysticta*	867	*Persea indica*	1.000	0.885	0.115	0.000
Fieldwork	9.10	Canary Islands	19/10/2022	-16.4025	28.4581	*F. polysticta*	867	*Viburnum rugosum*	1.000	0.957	0.043	0.000
Fieldwork	9.6	Canary Islands	19/10/2022	-16.4025	28.4581	*F. polysticta*	867	*Laurus novocanariensis*	0.444	1.000	0.000	0.000
Fieldwork	9.8	Canary Islands	19/10/2022	-16.4025	28.4581	*F. polysticta*	867	*Persea indica*	0.542	0.692	0.308	0.000
Fieldwork	9.9	Canary Islands	19/10/2022	-16.4025	28.4581	*F. polysticta*	867	*Viburnum rugosum*	1.000	0.793	0.207	0.000
LISU Herbarium	254710	Madeira	23/10/2004	-16.8982	32.6673	*F. polysticta*	300	*NA*	0.857	1.000	0.000	0.000
LISU Herbarium	261165	Madeira	31/07/2006	-17.1233	32.7178	*F. polysticta*	950	*Persea indica*	0.667	0.900	0.100	0.000
LISU Herbarium	256530	Madeira	08/11/2011	-16.8674	32.7219	*F. polysticta*	1096	*NA*	1.000	1.000	0.000	0.000
LISU Herbarium	261135	Madeira	31/07/2006	-16.9956	32.7289	*F. polysticta*	1200	*Persea indica*	0.250	1.000	0.000	0.000
LISU Herbarium	256550	Madeira	17/11/2005	-16.9209	32.7301	*F. polysticta*	960	*NA*	1.000	1.000	0.000	0.000
LISU Herbarium	261019	Madeira	21/07/2005	-16.9102	32.7303	*F. polysticta*	900	*Laurus novocanariensis*	1.000	1.000	0.000	0.000
LISU Herbarium	256546	Madeira	24/07/2004	-16.9102	32.7303	*F. polysticta*	850	*NA*	0.933	0.571	0.429	0.000
LISU Herbarium	251746	Madeira	22/07/2003	-16.8889	32.7306	*F. polysticta*	850	*NA*	1.000	1.000	0.000	0.000
LISU Herbarium	156120	Madeira	10/05/1990	-16.8782	32.7308	*F. polysticta*	750	*NA*	0.750	1.000	0.000	0.000
LISU Herbarium	156124	Madeira	19/10/1990	-16.8782	32.7308	*F. polysticta*	890	*Species unknown*	0.000	0.000	0.000	0.000
LISU Herbarium	256539	Madeira	06/08/2012	-16.8676	32.7309	*F. polysticta*	777	*NA*	1.000	1.000	0.000	0.000
LISU Herbarium	165813	Madeira	10/05/1990	-16.8676	32.7309	*F. polysticta*	750	*Species unknown*	0.429	1.000	0.000	0.000
LISU Herbarium	261257	Madeira	26/07/2006	-17.0704	32.7368	*F. polysticta*	1000	*Heberdenia excelsa*	1.000	1.000	0.000	0.000
LISU Herbarium	256532	Madeira	24/05/2012	-16.9211	32.7391	*F. polysticta*	913	*NA*	0.571	1.000	0.000	0.000
LISU Herbarium	261211	Madeira	03/03/2015	-16.9104	32.7393	*F. polysticta*	850	*Ocotea foetens*	1.000	1.000	0.000	0.000
LISU Herbarium	254636	Madeira	24/07/2004	-16.9104	32.7393	*F. polysticta*	870	*NA*	0.929	0.000	1.000	0.000
LISU Herbarium	256551	Madeira	16/11/2005	-16.9104	32.7393	*F. polysticta*	850	*NA*	1.000	1.000	0.000	0.000
LISU Herbarium	260935	Madeira	07/08/2007	-16.9213	32.7481	*F. polysticta*	950	*Persea indica*	0.000	0.000	0.000	0.000
LISU Herbarium	249917	Madeira	07/08/2001	-16.9213	32.7481	*F. polysticta*	1070	*NA*	1.000	1.000	0.000	0.000
LISU Herbarium	165814	Madeira	09/05/1990	-17.2095	32.7524	*F. polysticta*	650	*Species unknown*	0.400	1.000	0.000	0.000

LISU Herbarium	254184	Madeira	29/07/2003	-17.1348	32.7537	*F. polysticta*	1100	*NA*	1.000	0.300	0.700	0.000
LISU Herbarium	256540	Madeira	19/09/2012	-17.1348	32.7537	*F. polysticta*	1018	*NA*	1.000	0.964	0.036	0.000
LISU Herbarium	260934	Madeira	07/08/2007	-16.877	32.7608	*F. polysticta*	950	*Laurus novocanariensis*	0.750	1.000	0.000	0.000
LISU Herbarium	256536	Madeira	04/06/2012	-16.9219	32.7752	*F. polysticta*	884	*NA*	1.000	1.000	0.000	0.000
LISU Herbarium	261164	Madeira	03/08/2006	-16.9968	32.783	*F. polysticta*	950	*Laurus novocanariensis*	0.909	0.850	0.150	0.000
LISU Herbarium	256554	Madeira	27/06/2012	-16.9754	32.7834	*F. polysticta*	425	*Species unknown*	0.667	0.833	0.167	0.000
LISU Herbarium	256537	Madeira	26/07/2012	-16.9647	32.7835	*F. polysticta*	615	*NA*	1.000	1.000	0.000	0.000
LISU Herbarium	156122	Madeira	11/10/1990	-17.1357	32.7898	*F. polysticta*	1200	*Species unknown*	1.000	1.000	0.000	0.000
LISU Herbarium	261180	Madeira	22/07/2005	-17.0823	32.7907	*F. polysticta*	850	*Laurus novocanariensis*	1.000	1.000	0.000	0.000
LISU Herbarium	256543	Madeira	20/03/2013	-17.0823	32.7907	*F. polysticta*	1180	*NA*	0.538	1.000	0.000	0.000
LISU Herbarium	166303	Madeira	12/04/1988	-16.9329	32.7931	*F. polysticta*	500	*Species unknown*	0.917	1.000	0.000	0.000
LISU Herbarium	166304	Madeira	12/04/1988	-16.9116	32.7934	*F. polysticta*	600	*NA*	1.000	0.000	1.000	0.000
LISU Herbarium	256541	Madeira	25/09/2012	-17.1791	32.8161	*F. polysticta*	441	*NA*	0.833	0.600	0.400	0.000
Fieldwork	MD1.1	Madeira	29/11/2022	-17.1405	32.8101	*F. polysticta*	1398	*Laurus novocanariensis*	0.857	1.000	0.000	0.000
Fieldwork	MD1.2	Madeira	29/11/2022	-17.1405	32.8101	*F. polysticta*	1398	*Ocotea foetens*	0.667	0.900	0.100	0.000
Fieldwork	MD1.3	Madeira	29/11/2022	-17.1405	32.8101	*F. polysticta*	1398	*Ocotea foetens*	1.000	1.000	0.000	0.000
Fieldwork	MD1.4	Madeira	29/11/2022	-17.1405	32.8101	*F. polysticta*	1398	*Ocotea foetens*	0.250	1.000	0.000	0.000
Fieldwork	MD1.5	Madeira	29/11/2022	-17.1405	32.8101	*F. polysticta*	1398	*Laurus novocanariensis*	1.000	1.000	0.000	0.000
Fieldwork	MD12.1	Madeira	29/11/2022	-17.1142	32.7911	*F. polysticta*	584	*Laurus novocanariensis*	1.000	1.000	0.000	0.000
Fieldwork	MD12.2	Madeira	29/11/2022	-17.1142	32.7911	*F. polysticta*	584	*Persea indica*	0.933	0.571	0.429	0.125
Fieldwork	MD12.3	Madeira	29/11/2022	-17.1142	32.7911	*F. polysticta*	584	*Ocotea foetens*	1.000	1.000	0.000	0.000
Fieldwork	MD12.5	Madeira	29/11/2022	-17.1142	32.7911	*F. polysticta*	584	*Persea indica*	0.750	1.000	0.000	0.000
Fieldwork	MD12.7	Madeira	29/11/2022	-17.1142	32.7911	*F. polysticta*	584	*Laurus novocanariensis*	0.000	0.000	0.000	0.000
Fieldwork	MD13.1	Madeira	29/11/2022	-17.1136	32.7867	*F. polysticta*	721	*Ocotea foetens*	1.000	1.000	0.000	0.000
Fieldwork	MD13.10	Madeira	29/11/2022	-17.1136	32.7867	*F. polysticta*	721	*Clethra arborea*	0.429	1.000	0.000	0.000

Fieldwork	MD13.11	Madeira	29/11/2022	-17.1136	32.7867	*F. polysticta*	721	*Ocotea foetens*	1.000	1.000	0.000	0.000
Fieldwork	MD13.3	Madeira	29/11/2022	-17.1136	32.7867	*F. polysticta*	721	*Ocotea foetens*	0.571	1.000	0.000	0.000
Fieldwork	MD13.4	Madeira	29/11/2022	-17.1136	32.7867	*F. polysticta*	721	*Ocotea foetens*	1.000	1.000	0.000	0.000
Fieldwork	MD16.1	Madeira	28/11/2022	-16.925	32.7847	*F. polysticta*	933	*Laurus novocanariensis*	0.929	0.000	1.000	0.000
Fieldwork	MD16.2	Madeira	28/11/2022	-16.925	32.7847	*F. polysticta*	933	*Laurus novocanariensis*	1.000	1.000	0.000	0.000
Fieldwork	MD16.4	Madeira	28/11/2022	-16.925	32.7847	*F. polysticta*	933	*Clethra arborea*	0.000	0.000	0.000	0.000
Fieldwork	MD16.6	Madeira	28/11/2022	-16.925	32.7847	*F. polysticta*	933	*Laurus novocanariensis*	1.000	1.000	0.000	0.000
Fieldwork	MD16.7	Madeira	28/11/2022	-16.925	32.7847	*F. polysticta*	933	*Laurus novocanariensis*	0.400	1.000	0.000	0.000
Fieldwork	MD17.1	Madeira	30/11/2022	-16.9769	32.77	*F. polysticta*	1074	*Ocotea foetens*	1.000	0.300	0.700	0.000
Fieldwork	MD17.10	Madeira	30/11/2022	-16.9769	32.77	*F. polysticta*	1074	*Ocotea foetens*	1.000	0.964	0.036	0.000
Fieldwork	MD17.7	Madeira	30/11/2022	-16.9769	32.77	*F. polysticta*	1074	*Ocotea foetens*	0.750	1.000	0.000	0.000
Fieldwork	MD17.8	Madeira	30/11/2022	-16.9769	32.77	*F. polysticta*	1074	*Ocotea foetens*	1.000	1.000	0.000	0.000
Fieldwork	MD17.9	Madeira	30/11/2022	-16.9769	32.77	*F. polysticta*	1074	*Ocotea foetens*	0.909	0.850	0.150	0.000
Fieldwork	MD18.1	Madeira	30/11/2022	-16.9797	32.7775	*F. polysticta*	670	*Laurus novocanariensis*	0.667	0.833	0.167	0.000
Fieldwork	MD18.2	Madeira	30/11/2022	-16.9797	32.7775	*F. polysticta*	670	*Laurus novocanariensis*	1.000	1.000	0.000	0.000
Fieldwork	MD18.3	Madeira	30/11/2022	-16.9797	32.7775	*F. polysticta*	670	*Laurus novocanariensis*	1.000	1.000	0.000	0.000
Fieldwork	MD18.4	Madeira	30/11/2022	-16.9797	32.7775	*F. polysticta*	670	*Laurus novocanariensis*	1.000	1.000	0.000	0.000
Fieldwork	MD18.6	Madeira	30/11/2022	-16.9797	32.7775	*F. polysticta*	670	*Laurus novocanariensis*	0.538	1.000	0.000	0.000
Fieldwork	MD2.1	Madeira	29/11/2022	-17.1578	32.8253	*F. polysticta*	831	*Laurus novocanariensis*	0.917	1.000	0.000	0.000
Fieldwork	MD2.10	Madeira	29/11/2022	-17.1578	32.8253	*F. polysticta*	831	*Vaccinium padifolium*	1.000	0.000	1.000	0.000
Fieldwork	MD2.11	Madeira	29/11/2022	-17.1578	32.8253	*F. polysticta*	831	*Erica platycodon*	0.833	0.600	0.400	0.000
Fieldwork	MD2.8	Madeira	29/11/2022	-17.1578	32.8253	*F. polysticta*	831	*Erica scoparia*	0.778	0.714	0.286	0.000
Fieldwork	MD2.9	Madeira	29/11/2022	-17.1578	32.8253	*F. polysticta*	831	*Laurus novocanariensis*	0.286	1.000	0.000	0.000
Fieldwork	MD3.1	Madeira	28/11/2022	-16.8853	32.7347	*F. polysticta*	879	*Clethra arborea*	1.000	0.367	0.633	0.091

Fieldwork	MD3.5	Madeira	28/11/2022	-16.8853	32.7347	*F. polysticta*	879	*Picconia excelsa*	1.000	0.667	0.333	0.000
Fieldwork	MD3.6	Madeira	28/11/2022	-16.8853	32.7347	*F. polysticta*	879	*Laurus novocanariensis*	1.000	0.500	0.500	0.000
Fieldwork	MD3.7	Madeira	28/11/2022	-16.8853	32.7347	*F. polysticta*	879	*Laurus novocanariensis*	1.000	0.500	0.500	0.333
Fieldwork	MD3.8	Madeira	28/11/2022	-16.8853	32.7347	*F. polysticta*	879	*Laurus novocanariensis*	1.000	1.000	0.000	0.000
Fieldwork	MD4.1	Madeira	28/11/2022	-16.8892	32.7408	*F. polysticta*	812	*Laurus novocanariensis*	0.600	1.000	0.000	0.000
Fieldwork	MD4.2	Madeira	28/11/2022	-16.8892	32.7408	*F. polysticta*	812	*Picconia excelsa*	1.000	1.000	0.000	0.000
Fieldwork	MD4.3	Madeira	28/11/2022	-16.8892	32.7408	*F. polysticta*	812	*Laurus novocanariensis*	0.939	0.913	0.087	0.048
Fieldwork	MD4.4	Madeira	28/11/2022	-16.8892	32.7408	*F. polysticta*	812	*Laurus novocanariensis*	1.000	1.000	0.000	0.000
Fieldwork	MD4.6	Madeira	28/11/2022	-16.8892	32.7408	*F. polysticta*	812	*Persea indica*	1.000	1.000	0.000	0.000
Fieldwork	MD7.1	Madeira	02/12/2022	-16.9061	32.7453	*F. polysticta*	790	*Laurus novocanariensis*	1.000	1.000	0.000	0.000
Fieldwork	MD7.2	Madeira	02/12/2022	-16.9061	32.7453	*F. polysticta*	790	*Laurus novocanariensis*	0.750	1.000	0.000	0.000
Fieldwork	MD7.4	Madeira	02/12/2022	-16.9061	32.7453	*F. polysticta*	790	*Ocotea foetens*	1.000	0.889	0.111	0.125
Fieldwork	MD7.5	Madeira	02/12/2022	-16.9061	32.7453	*F. polysticta*	790	*Laurus novocanariensis*	1.000	0.667	0.333	0.200
Fieldwork	MD7.6	Madeira	02/12/2022	-16.9061	32.7453	*F. polysticta*	790	*Laurus novocanariensis*	1.000	0.846	0.154	0.000
Fieldwork	MD8.10	Madeira	01/12/2022	-17.0186	32.7536	*F. polysticta*	1007	*Quercus robur*	1.000	1.000	0.000	0.000
Fieldwork	MD8.11	Madeira	01/12/2022	-17.0186	32.7536	*F. polysticta*	1007	*Quercus robur*	1.000	1.000	0.000	0.000
Fieldwork	MD8.12	Madeira	01/12/2022	-17.0186	32.7536	*F. polysticta*	1007	*Quercus robur*	1.000	1.000	0.000	0.000
Fieldwork	MD8.7	Madeira	01/12/2022	-17.0186	32.7536	*F. polysticta*	1007	*Hesperocyparis macrocarpa*	1.000	1.000	0.000	0.000
Fieldwork	MD8.8	Madeira	01/12/2022	-17.0186	32.7536	*F. polysticta*	1007	*Hesperocyparis macrocarpa*	1.000	0.974	0.026	0.579
AZU Herbarium	RG-2141	Azores	02/07/1992	-28.2973	38.4496	*P. canariensis*	560	*Laurus azorica*	0.667	1.000	0.000	0.000
AZU Herbarium	RG-2197	Azores	02/07/1992	-28.2721	38.4504	*P. canariensis*	575	*Laurus azorica*	0.267	1.000	0.000	0.000
AZU Herbarium	FP092	Azores	01/07/2000	-28.4593	38.5387	*P. canariensis*	230	*NA*	0.000	0.000	0.000	0.000
AZU Herbarium	RG_20090622_03	Azores	22/06/2009	-27.0682	38.6819	*P. canariensis*	59	*NA*	0.143	1.000	0.000	0.000
AZU Herbarium	PS1-ro03	Azores	30/10/2008	-27.2446	38.6836	*P. canariensis*	242	*NA*	0.000	0.000	0.000	0.000

AZU Herbarium	RG-3218	Azores	02/06/1978	-27.2645	38.7043	*P. canariensis*	475	*NA*	1.000	1.000	0.000	0.000
AZU Herbarium	RG-3196	Azores	02/06/1978	-27.2645	38.7043	*P. canariensis*	470	*NA*	0.000	0.000	0.000	0.000
AZU Herbarium	MT4_r012	Azores	21/10/2008	-27.2574	38.7289	*P. canariensis*	532	*NA*	0.667	1.000	0.000	0.000
AZU Herbarium	MT42-ro06	Azores	21/10/2008	-27.2574	38.7289	*P. canariensis*	532	*NA*	0.400	1.000	0.000	0.000
AZU Herbarium	MT1-ro02	Azores	21/10/2008	-27.2574	38.7289	*P. canariensis*	532	*NA*	1.000	1.000	0.000	0.000
AZU Herbarium	RG-20080621-02-d	Azores	21/06/2008	-27.164	38.7495	*P. canariensis*	250	*NA*	0.000	0.000	0.000	0.000
AZU Herbarium	RG-5236	Azores	06/06/2003	-27.302	38.753	*P. canariensis*	514	*NA*	0.267	1.000	0.000	0.000
LISU Herbarium	231722	Azores	21/06/1999	-27.9844	38.645	*P. canariensis*	400	*NA*	0.200	1.000	0.000	0.000
LISU Herbarium	256261	Azores	11/09/2008	-31.1689	39.4662	*P. canariensis*	300	*NA*	0.133	1.000	0.000	0.000
LISU Herbarium	231719	Azores	22/06/1999	-27.8609	38.5681	*P. canariensis*	650	*NA*	0.167	1.000	0.000	0.000
LISU Herbarium	225792	Azores	01/07/1997	-27.9283	38.5835	*P. canariensis*		*NA*	0.000	0.000	0.000	0.000
LISU Herbarium	231723	Azores	21/06/1999	-27.9804	38.6432	*P. canariensis*	300	*Species unknown*	0.000	0.000	0.000	0.000
LISU Herbarium	231720	Azores	21/06/1999	-27.9804	38.6432	*P. canariensis*	269	*NA*	0.200	1.000	0.000	0.000
LISU Herbarium	225793	Azores	01/08/1997	-27.9804	38.6432	*P. canariensis*		*NA*	0.000	0.000	0.000	0.000
LISU Herbarium	231716	Azores	19/06/1999	-27.9871	38.6464	*P. canariensis*	400	*NA*	0.200	1.000	0.000	0.000
LISU Herbarium	231718	Azores	23/06/1999	-28.0768	38.6528	*P. canariensis*	950	*NA*	0.091	1.000	0.000	0.000
LISU Herbarium	225794	Azores	22/06/1999	-28.0493	38.654	*P. canariensis*	690	*NA*	0.000	0.000	0.000	0.000
LISU Herbarium	231721	Azores	22/06/1999	-28.0493	38.654	*P. canariensis*	690	*Species unknown*	0.375	1.000	0.000	0.000
LISU Herbarium	231717	Azores	22/06/1999	-28.0493	38.654	*P. canariensis*	690	*Species unknown*	0.167	1.000	0.000	0.000
LISU Herbarium	256262	Azores	13/09/2008	-28.0345	38.664	*P. canariensis*	108	*NA*	0.000	0.000	0.000	0.000
LISU Herbarium	257463	Azores	28/04/1983	-27.2693	38.7379	*P. canariensis*	540	*NA*	0.000	0.000	0.000	0.000
LISU Herbarium	257469	Azores	08/08/1987	-27.2693	38.7379	*P. canariensis*	500	*NA*	0.000	0.000	0.000	0.000
Fieldwork	1.1	Canary Islands	19/10/2022	-16.2678	28.5344	*P. canariensis*	980	*Laurus novocanariensis*	0.969	0.210	0.790	0.154
Fieldwork	1.2	Canary Islands	19/10/2022	-16.2678	28.5344	*P. canariensis*	980	*Myrica faya*	0.758	0.420	0.580	0.381
Fieldwork	1.3	Canary Islands	19/10/2022	-16.2678	28.5344	*P. canariensis*	980	*Laurus novocanariensis*	1.000	0.073	0.927	0.667
Fieldwork	1.4	Canary Islands	19/10/2022	-16.2678	28.5344	*P. canariensis*	980	*Laurus novocanariensis*	0.810	0.676	0.324	0.196

Fieldwork	1.5	Canary Islands	19/10/2022	-16.2678	28.5344	*P. canariensis*	980	*Laurus novocanariensis*	0.897	0.986	0.014	0.174
Fieldwork	10.1	Canary Islands	21/10/2022	-16.8161	28.3225	*P. canariensis*	998	*Persea indica*	0.053	1.000	0.000	0.000
Fieldwork	10.4	Canary Islands	21/10/2022	-16.8161	28.3225	*P. canariensis*	998	*Laurus novocanariensis*	0.000	0.000	0.000	0.000
Fieldwork	10.5	Canary Islands	21/10/2022	-16.8161	28.3225	*P. canariensis*	998	*Laurus novocanariensis*	0.400	1.000	0.000	0.000
Fieldwork	10.7	Canary Islands	21/10/2022	-16.8161	28.3225	*P. canariensis*	998	*Laurus novocanariensis*	0.500	0.750	0.250	0.000
Fieldwork	10.9	Canary Islands	21/10/2022	-16.8161	28.3225	*P. canariensis*	998	*Laurus novocanariensis*	1.000	0.000	1.000	0.000
Fieldwork	12.1	Canary Islands	19/10/2022	-16.405	28.4542	*P. canariensis*	941	*Laurus novocanariensis*	0.000	0.000	0.000	0.000
Fieldwork	12.2	Canary Islands	19/10/2022	-16.405	28.4542	*P. canariensis*	941	*Laurus novocanariensis*	0.731	0.000	1.000	0.000
Fieldwork	12.4	Canary Islands	19/10/2022	-16.405	28.4542	*P. canariensis*	941	*Laurus novocanariensis*	0.844	1.000	0.000	0.079
Fieldwork	12.5	Canary Islands	19/10/2022	-16.405	28.4542	*P. canariensis*	941	*Laurus novocanariensis*	1.000	0.000	1.000	0.000
Fieldwork	12.6	Canary Islands	19/10/2022	-16.405	28.4542	*P. canariensis*	941	*Laurus novocanariensis*	0.000	0.000	0.000	0.000
Fieldwork	13.1	Canary Islands	18/10/2022	-16.3064	28.5369	*P. canariensis*	809	*Ilex canariensis*	0.884	0.246	0.754	0.133
Fieldwork	13.3	Canary Islands	18/10/2022	-16.3064	28.5369	*P. canariensis*	809	*Ilex perado*	0.913	0.667	0.333	0.143
Fieldwork	13.4	Canary Islands	18/10/2022	-16.3064	28.5369	*P. canariensis*	809	*Laurus novocanariensis*	0.489	0.522	0.478	0.000
Fieldwork	13.5	Canary Islands	18/10/2022	-16.3064	28.5369	*P. canariensis*	809	*Laurus novocanariensis*	1.000	0.359	0.641	0.214
Fieldwork	13.6	Canary Islands	18/10/2022	-16.3064	28.5369	*P. canariensis*	809	*Prunus lusitanica*	1.000	0.000	1.000	0.000
Fieldwork	14.1	Canary Islands	18/10/2022	-16.2956	28.5295	*P. canariensis*	850	*Laurus novocanariensis*	1.000	0.000	1.000	0.000
Fieldwork	14.2	Canary Islands	18/10/2022	-16.2956	28.5295	*P. canariensis*	850	*Laurus novocanariensis*	0.000	0.000	0.000	0.000
Fieldwork	14.3	Canary Islands	18/10/2022	-16.2956	28.5295	*P. canariensis*	850	*Laurus novocanariensis*	0.067	1.000	0.000	0.000
Fieldwork	14.6	Canary Islands	18/10/2022	-16.2956	28.5295	*P. canariensis*	850	*Laurus novocanariensis*	0.000	0.000	0.000	0.000
Fieldwork	14.8	Canary Islands	18/10/2022	-16.2956	28.5295	*P. canariensis*	850	*Laurus novocanariensis*	0.000	0.000	0.000	0.000

Fieldwork	15.1	Canary Islands	18/10/2022	-16.2906	28.5325	*P. canariensis*	921	*Laurus novocanariensis*	0.905	0.000	1.000	0.000
Fieldwork	15.2	Canary Islands	18/10/2022	-16.2906	28.5325	*P. canariensis*	921	*Laurus novocanariensis*	0.050	1.000	0.000	0.000
Fieldwork	15.3	Canary Islands	18/10/2022	-16.2906	28.5325	*P. canariensis*	921	*Laurus novocanariensis*	0.611	0.000	1.000	0.000
Fieldwork	15.4	Canary Islands	18/10/2022	-16.2906	28.5325	*P. canariensis*	921	*Laurus novocanariensis*	0.000	0.000	0.000	0.000
Fieldwork	15.5	Canary Islands	18/10/2022	-16.2906	28.5325	*P. canariensis*	921	*Prunus lusitanica*	0.714	0.000	1.000	0.000
Fieldwork	2.1	Canary Islands	17/10/2022	-16.1697	28.5594	*P. canariensis*	783	*Ilex canariensis*	0.750	1.000	0.000	0.000
Fieldwork	2.2	Canary Islands	17/10/2022	-16.1697	28.5594	*P. canariensis*	783	*Ilex canariensis*	0.711	0.932	0.068	0.200
Fieldwork	2.3	Canary Islands	17/10/2022	-16.1697	28.5594	*P. canariensis*	783	*Laurus novocanariensis*	1.000	0.027	0.973	1.000
Fieldwork	2.4	Canary Islands	17/10/2022	-16.1697	28.5594	*P. canariensis*	783	*Laurus novocanariensis*	0.750	0.143	0.857	0.000
Fieldwork	2.5	Canary Islands	17/10/2022	-16.1697	28.5594	*P. canariensis*	783	*Persea indica*	0.811	0.953	0.047	0.122
Fieldwork	3.1	Canary Islands	17/10/2022	-16.1778	28.5575	*P. canariensis*	833	*Laurus novocanariensis*	0.901	0.681	0.319	0.210
Fieldwork	3.2	Canary Islands	17/10/2022	-16.1778	28.5575	*P. canariensis*	833	*Ilex canariensis*	0.878	0.819	0.181	0.186
Fieldwork	3.3	Canary Islands	17/10/2022	-16.1778	28.5575	*P. canariensis*	833	*Laurus novocanariensis*	0.691	0.553	0.447	0.000
Fieldwork	3.4	Canary Islands	17/10/2022	-16.1778	28.5575	*P. canariensis*	833	*Laurus novocanariensis*	0.375	1.000	0.000	0.000
Fieldwork	3.8	Canary Islands	17/10/2022	-16.1778	28.5575	*P. canariensis*	833	*Laurus novocanariensis*	0.989	0.841	0.159	0.189
Fieldwork	5.1	Canary Islands	17/10/2022	-16.2169	28.5469	*P. canariensis*	650	*Laurus novocanariensis*	0.945	0.217	0.783	0.067
Fieldwork	5.2	Canary Islands	17/10/2022	-16.2169	28.5469	*P. canariensis*	650	*Laurus novocanariensis*	0.917	0.114	0.886	0.000
Fieldwork	5.3	Canary Islands	17/10/2022	-16.2169	28.5469	*P. canariensis*	650	*Laurus novocanariensis*	1.000	0.000	1.000	0.000
Fieldwork	5.4	Canary Islands	17/10/2022	-16.2169	28.5469	*P. canariensis*	650	*Ilex canariensis*	0.870	0.000	1.000	0.000
Fieldwork	5.5	Canary Islands	17/10/2022	-16.2169	28.5469	*P. canariensis*	650	*Laurus novocanariensis*	1.000	1.000	0.000	0.074
Fieldwork	6.1	Canary Islands	19/10/2022	-16.2278	28.5425	*P. canariensis*	860	*Ilex canariensis*	0.877	0.500	0.500	0.200
Fieldwork	6.2	Canary Islands	19/10/2022	-16.2278	28.5425	*P. canariensis*	860	*Ilex canariensis*	1.000	0.483	0.517	0.167

Fieldwork	6.3	Canary Islands	19/10/2022	-16.2278	28.5425	*P. canariensis*	860	*Laurus novocanariensis*	0.807	0.543	0.457	0.040
Fieldwork	6.4	Canary Islands	19/10/2022	-16.2278	28.5425	*P. canariensis*	860	*Laurus novocanariensis*	0.500	1.000	0.000	0.188
Fieldwork	6.5	Canary Islands	19/10/2022	-16.2278	28.5425	*P. canariensis*	860	*Laurus novocanariensis*	0.655	0.368	0.632	0.286
Fieldwork	7.1	Canary Islands	17/10/2022	-16.2856	28.5308	*P. canariensis*	932	*Laurus novocanariensis*	0.938	0.067	0.933	0.000
Fieldwork	7.2	Canary Islands	17/10/2022	-16.2856	28.5308	*P. canariensis*	932	*Laurus novocanariensis*	0.556	1.000	0.000	0.000
Fieldwork	7.3	Canary Islands	17/10/2022	-16.2856	28.5308	*P. canariensis*	932	*Laurus novocanariensis*	0.300	1.000	0.000	0.000
Fieldwork	7.4	Canary Islands	17/10/2022	-16.2856	28.5308	*P. canariensis*	932	*Laurus novocanariensis*	0.667	0.933	0.067	0.000
Fieldwork	7.5	Canary Islands	17/10/2022	-16.2856	28.5308	*P. canariensis*	932	*Laurus novocanariensis*	0.000	0.000	0.000	0.000
Fieldwork	9.1	Canary Islands	19/10/2022	-16.4025	28.4581	*P. canariensis*	867	*Persea indica*	1.000	0.027	0.973	0.000
Fieldwork	9.2	Canary Islands	19/10/2022	-16.4025	28.4581	*P. canariensis*	867	*Persea indica*	0.742	0.652	0.348	0.000
Fieldwork	9.3	Canary Islands	19/10/2022	-16.4025	28.4581	*P. canariensis*	867	*Laurus novocanariensis*	1.000	0.192	0.808	0.100
Fieldwork	9.4	Canary Islands	19/10/2022	-16.4025	28.4581	*P. canariensis*	867	*Laurus novocanariensis*	1.000	0.000	1.000	0.000
Fieldwork	9.5	Canary Islands	19/10/2022	-16.4025	28.4581	*P. canariensis*	867	*Laurus novocanariensis*	0.545	0.750	0.250	0.000
E Herbarium	Herbarium C.C. Townsed 78/233	Canary Islands	28/03/1978	-16.8236	28.338	*P. canariensis*	1000	*NA*	0.867	1.000	0.000	0.000
E Herbarium	Herbarium C.C. Townsed 78/218	Canary Islands	28/03/1978	-16.8236	28.338	*P. canariensis*	1000	*Species unknown*	0.714	1.000	0.000	0.000
E Herbarium	5892	Canary Islands	06/04/1977	-16.3841	28.3709	*P. canariensis*	1100	*NA*	0.200	1.000	0.000	0.000
E Herbarium	5910	Canary Islands	06/04/1977	-16.3841	28.3709	*P. canariensis*	1100	*NA*	1.000	0.000	1.000	0.000
E Herbarium	5763	Canary Islands	02/04/1977	-16.2796	28.5316	*P. canariensis*	950	*Species unknown*	1.000	1.000	0.000	0.154
E Herbarium	Herbarium C.C. Townsed 78/160	Canary Islands	26/03/1978	-16.2796	28.5316	*P. canariensis*	970	*Species unknown*	1.000	1.000	0.000	0.167
E Herbarium	Herbarium C.C. Townsed 78/139	Canary Islands	26/03/1978	-16.2796	28.5316	*P. canariensis*	970	*Species unknown*	0.677	1.000	0.000	0.000
E Herbarium	Herbarium C.C. Townsed 78/164	Canary Islands	26/03/1978	-16.2796	28.5316	*P. canariensis*	970	*Species unknown*	0.842	1.000	0.000	0.000
E Herbarium	A.C. Crundwell Herbarium 163	Canary Islands	25/03/1978	-16.2642	28.5334	*P. canariensis*		*Species unknown*	0.652	0.467	0.533	0.143

E Herbarium	5924	Canary Islands	07/04/1977	-16.2068	28.549	*P. canariensis*	900	*Species unknown*	1.000	1.000	0.000	0.650
TFC Herbarium	1515	Canary Islands	30/04/1986	-16.8212	28.3386	*P. canariensis*	670	*Persea indica*	0.273	0.000	1.000	0.000
TFC Herbarium	1540	Canary Islands	26/02/1986	-16.8212	28.3386	*P. canariensis*	670	*Laurus azorica*	1.000	0.286	0.714	0.000
TFC Herbarium	1553	Canary Islands	26/02/1986	-16.8212	28.3386	*P. canariensis*	670	*Persea indica*	0.571	0.750	0.250	0.000
TFC Herbarium	1607	Canary Islands	27/10/1986	-16.8212	28.3386	*P. canariensis*	670	*Ilex canariensis*	0.316	0.833	0.167	0.000
TFC Herbarium	1611	Canary Islands	27/10/1986	-16.8212	28.3386	*P. canariensis*	670	*Ilex canariensis*	0.167	1.000	0.000	0.000
TFC Herbarium	1563	Canary Islands	27/10/1986	-16.8212	28.3386	*P. canariensis*	670	*Laurus novocanariensis*	0.200	1.000	0.000	0.000
TFC Herbarium	3871	Canary Islands	05/05/1986	-16.3934	28.4432	*P. canariensis*	1130	*NA*	1.000	0.000	1.000	0.000
TFC Herbarium	3924	Canary Islands	05/05/1986	-16.3985	28.4442	*P. canariensis*	1060	*NA*	0.833	0.000	1.000	0.000
TFC Herbarium	2400	Canary Islands	05/11/1981	-16.4037	28.449	*P. canariensis*	1000	*Laurus azorica*	1.000	0.692	0.308	0.222
TFC Herbarium	2619	Canary Islands	10/08/1984	-16.4037	28.449	*P. canariensis*	1000	*NA*	0.500	1.000	0.000	0.000
TFC Herbarium	2963	Canary Islands	17/06/1985	-16.4037	28.449	*P. canariensis*	1000	*Ilex canariensis*	0.545	0.167	0.833	0.000
TFC Herbarium	2694	Canary Islands	01/10/1984	-16.4051	28.4554	*P. canariensis*	920	*Persea indica*	0.750	0.167	0.833	0.000
TFC Herbarium	2699	Canary Islands	01/10/1984	-16.4051	28.4554	*P. canariensis*	920	*Persea indica*	0.125	1.000	0.000	0.000
TFC Herbarium	2328	Canary Islands	05/12/1983	-16.4051	28.4554	*P. canariensis*	920	*Laurus novocanariensis*	0.063	1.000	0.000	0.000
TFC Herbarium	2298	Canary Islands	13/02/1984	-16.4021	28.4572	*P. canariensis*	860	*NA*	0.273	0.000	1.000	0.000
TFC Herbarium	2070	Canary Islands	20/10/1981	-16.4021	28.4572	*P. canariensis*	860	*Persea indica*	0.375	0.333	0.667	0.000
TFC Herbarium	2200	Canary Islands	07/11/1983	-16.4039	28.4584	*P. canariensis*	830	*NA*	0.091	1.000	0.000	0.000
TFC Herbarium	2284	Canary Islands	13/02/1984	-16.4039	28.4584	*P. canariensis*	830	*Persea indica*	0.250	1.000	0.000	0.000
TFC Herbarium	2607	Canary Islands	10/08/1984	-16.4039	28.4584	*P. canariensis*	830	*Persea indica*	0.083	1.000	0.000	0.000
TFC Herbarium	2733	Canary Islands	17/10/1984	-16.4039	28.4584	*P. canariensis*	830	*NA*	0.250	1.000	0.000	0.000
TFC Herbarium	3182	Canary Islands	04/11/1985	-16.4039	28.4584	*P. canariensis*	830	*Persea indica*	0.400	0.500	0.500	0.000
TFC Herbarium	3187	Canary Islands	04/11/1985	-16.4039	28.4584	*P. canariensis*	830	*Persea indica*	0.375	1.000	0.000	0.000
TFC Herbarium	3189	Canary Islands	04/11/1985	-16.4039	28.4584	*P. canariensis*	830	*Persea indica*	0.429	1.000	0.000	0.000
TFC Herbarium	2186	Canary Islands	07/11/1983	-16.4039	28.4584	*P. canariensis*	830	*humid soil*	0.000	0.000	0.000	0.000
TFC Herbarium	3585	Canary Islands	13/02/1986	-16.4039	28.4584	*P. canariensis*	830	*NA*	0.000	0.000	0.000	0.000
TFC Herbarium	3337	Canary Islands	20/11/1985	-16.4039	28.4584	*P. canariensis*	830	*NA*	0.364	0.000	1.000	0.000
TFC Herbarium	17743	Canary Islands	22/04/2001	-16.2712	28.5305	*P. canariensis*	705	*Laurus novocanariensis*	0.600	0.167	0.833	0.000

TFC Herbarium	17772	Canary Islands	22/04/2001	-16.2712	28.5305	*P. canariensis*	705	*Laurus novocanariensis*	0.143	0.000	1.000	0.000
TFC Herbarium	17645	Canary Islands	22/04/2001	-16.2712	28.5305	*P. canariensis*	705	*Laurus novocanariensis*	0.077	1.000	0.000	0.000
TFC Herbarium	16066	Canary Islands	15/03/2000	-16.1859	28.5527	*P. canariensis*	700	*Ilex canariensis*	1.000	0.000	1.000	0.000
TFC Herbarium	16075	Canary Islands	15/03/2000	-16.1859	28.5527	*P. canariensis*	700	*Laurus novocanariensis*	0.273	0.333	0.667	0.000
TFC Herbarium	16076	Canary Islands	15/03/2020	-16.1859	28.5527	*P. canariensis*	700	*Laurus novocanariensis*	0.000	0.000	0.000	0.000
TFC Herbarium	16093	Canary Islands	15/03/2020	-16.1859	28.5527	*P. canariensis*	700	*Laurus novocanariensis*	0.556	0.400	0.600	0.000
TFC Herbarium	16116	Canary Islands	13/03/2000	-16.1859	28.5527	*P. canariensis*	700	*Ilex canariensis*	0.182	1.000	0.000	0.000
TFC Herbarium	16118	Canary Islands	13/03/2000	-16.1859	28.5527	*P. canariensis*	700	*Ilex canariensis*	0.200	0.000	1.000	0.000
TFC Herbarium	16061	Canary Islands	15/03/2000	-16.1859	28.5527	*P. canariensis*	700	*Laurus novocanariensis*	0.818	0.444	0.556	0.250
TFC Herbarium	16067	Canary Islands	15/03/2000	-16.1859	28.5527	*P. canariensis*	700	*Laurus novocanariensis*	0.538	0.571	0.429	0.000
TFC Herbarium	16078	Canary Islands	15/03/2000	-16.1859	28.5527	*P. canariensis*	700	*Laurus novocanariensis*	0.545	0.833	0.167	0.000
TFC Herbarium	17949	Canary Islands	25/04/2001	-16.1832	28.5544	*P. canariensis*	780	*Laurus novocanariensis*	0.000	0.000	0.000	0.000
TFC Herbarium	17969	Canary Islands	25/04/2001	-16.1832	28.5544	*P. canariensis*	780	*Laurus novocanariensis*	0.455	0.400	0.600	0.000
TFC Herbarium	18029	Canary Islands	25/04/2001	-16.1832	28.5544	*P. canariensis*	780	*Laurus novocanariensis*	0.357	0.400	0.600	0.500
TFC Herbarium	18086	Canary Islands	25/04/2001	-16.1832	28.5544	*P. canariensis*	780	*Laurus novocanariensis*	0.071	1.000	0.000	0.000
TFC Herbarium	18158	Canary Islands	25/04/2001	-16.1832	28.5544	*P. canariensis*	780	*Laurus novocanariensis*	0.267	0.250	0.750	0.000
TFC Herbarium	18239	Canary Islands	25/04/2001	-16.1832	28.5544	*P. canariensis*	780	*Laurus novocanariensis*	0.500	1.000	0.000	0.000
TFC Herbarium	18356	Canary Islands	25/04/2001	-16.1832	28.5544	*P. canariensis*	780	*Laurus novocanariensis*	0.500	0.500	0.500	0.000
TFC Herbarium	18362	Canary Islands	25/04/2001	-16.1832	28.5544	*P. canariensis*	780	*Laurus novocanariensis*	0.500	0.500	0.500	0.000
TFC Herbarium	11864	Canary Islands	25/10/2000	-16.1747	28.5589	*P. canariensis*	900	*Erica platycodon*	0.818	0.111	0.889	0.000
VAL Herbarium	7514	Canary Islands	04/02/2005	-17.224	28.1093	*P. canariensis*	1180	*Species unknown*	1.000	0.222	0.778	0.000
VAL Herbarium	7402	Canary Islands	05/02/2005	-17.2159	28.138	*P. canariensis*	1200	*Species unknown*	1.000	0.000	1.000	0.000

VAL Herbarium	7482	Canary Islands	06/02/2005	-16.8259	28.329	*P. canariensis*	800	*NA*	0.000	0.000	0.000	0.000
VAL Herbarium	1385	Canary Islands	17/03/1986	-16.8212	28.3386	*P. canariensis*		*NA*	0.583	1.000	0.000	0.000
Fieldwork	MD1.1	Madeira	29/11/2022	-17.1405	32.8101	*P. canariensis*	1398	*Laurus novocanariensis*	1.000	0.920	0.080	0.261
Fieldwork	MD1.2	Madeira	29/11/2022	-17.1405	32.8101	*P. canariensis*	1398	*Ocotea foetens*	1.000	0.559	0.441	0.211
Fieldwork	MD1.3	Madeira	29/11/2022	-17.1405	32.8101	*P. canariensis*	1398	*Ocotea foetens*	0.730	0.935	0.065	0.140
Fieldwork	MD1.4	Madeira	29/11/2022	-17.1405	32.8101	*P. canariensis*	1398	*Ocotea foetens*	0.625	0.933	0.067	0.071
Fieldwork	MD1.5	Madeira	29/11/2022	-17.1405	32.8101	*P. canariensis*	1398	*Laurus novocanariensis*	1.000	0.804	0.196	0.297
Fieldwork	MD10.1	Madeira	30/11/2022	-16.9392	32.8064	*P. canariensis*	635	*Persea indica*	1.000	1.000	0.000	0.091
Fieldwork	MD10.2	Madeira	30/11/2022	-16.9392	32.8064	*P. canariensis*	635	*Persea indica*	0.828	0.667	0.333	0.688
Fieldwork	MD10.3	Madeira	30/11/2022	-16.9392	32.8064	*P. canariensis*	635	*Persea indica*	1.000	0.941	0.059	0.250
Fieldwork	MD10.4	Madeira	30/11/2022	-16.9392	32.8064	*P. canariensis*	635	*Laurus novocanariensis*	1.000	0.913	0.087	0.048
Fieldwork	MD10.5	Madeira	30/11/2022	-16.9392	32.8064	*P. canariensis*	635	*Laurus novocanariensis*	0.857	0.472	0.528	0.059
Fieldwork	MD11.1	Madeira	30/11/2022	-16.9372	32.7939	*P. canariensis*	640	*Ocotea foetens*	1.000	1.000	0.000	0.333
Fieldwork	MD11.2	Madeira	30/11/2022	-16.9372	32.7939	*P. canariensis*	640	*Ocotea foetens*	1.000	0.667	0.333	0.000
Fieldwork	MD11.3	Madeira	30/11/2022	-16.9372	32.7939	*P. canariensis*	640	*Ocotea foetens*	0.333	0.000	1.000	0.000
Fieldwork	MD11.4	Madeira	30/11/2022	-16.9372	32.7939	*P. canariensis*	640	*Ocotea foetens*	1.000	1.000	0.000	0.500
Fieldwork	MD11.5	Madeira	30/11/2022	-16.9372	32.7939	*P. canariensis*	640	*Laurus novocanariensis*	0.286	0.500	0.500	0.000
Fieldwork	MD12.1	Madeira	29/11/2022	-17.1142	32.7911	*P. canariensis*	584	*Laurus novocanariensis*	1.000	0.769	0.231	0.300
Fieldwork	MD12.2	Madeira	29/11/2022	-17.1142	32.7911	*P. canariensis*	584	*Persea indica*	1.000	1.000	0.000	0.182
Fieldwork	MD12.3	Madeira	29/11/2022	-17.1142	32.7911	*P. canariensis*	584	*Ocotea foetens*	1.000	1.000	0.000	0.118
Fieldwork	MD12.4	Madeira	29/11/2022	-17.1142	32.7911	*P. canariensis*	584	*Persea indica*	0.333	0.250	0.750	0.000
Fieldwork	MD12.5	Madeira	29/11/2022	-17.1142	32.7911	*P. canariensis*	584	*Persea indica*	0.581	0.278	0.722	0.000
Fieldwork	MD13.1	Madeira	29/11/2022	-17.1136	32.7867	*P. canariensis*	721	*Ocotea foetens*	1.000	0.652	0.348	0.000
Fieldwork	MD13.2	Madeira	29/11/2022	-17.1136	32.7867	*P. canariensis*	721	*Ocotea foetens*	0.368	1.000	0.000	0.143
Fieldwork	MD13.3	Madeira	29/11/2022	-17.1136	32.7867	*P. canariensis*	721	*Ocotea foetens*	0.486	0.941	0.059	0.125
Fieldwork	MD13.4	Madeira	29/11/2022	-17.1136	32.7867	*P. canariensis*	721	*Ocotea foetens*	1.000	0.000	1.000	0.000
Fieldwork	MD13.5	Madeira	29/11/2022	-17.1136	32.7867	*P. canariensis*	721	*Ocotea foetens*	0.720	0.889	0.111	0.000

Fieldwork	MD14.1	Madeira	28/11/2022	-16.9075	32.7836	*P. canariensis*	899	*Laurus novocanariensis*	1.000	0.353	0.647	0.167
Fieldwork	MD14.2	Madeira	28/11/2022	-16.9075	32.7836	*P. canariensis*	899	*Laurus novocanariensis*	1.000	1.000	0.000	0.364
Fieldwork	MD14.3	Madeira	28/11/2022	-16.9075	32.7836	*P. canariensis*	899	*Laurus novocanariensis*	1.000	0.065	0.935	0.500
Fieldwork	MD14.6	Madeira	28/11/2022	-16.9075	32.7836	*P. canariensis*	899	*Persea indica*	0.625	0.600	0.400	0.000
Fieldwork	MD14.7	Madeira	28/11/2022	-16.9075	32.7836	*P. canariensis*	899	*Laurus novocanariensis*	1.000	0.235	0.765	0.250
Fieldwork	MD16.1	Madeira	28/11/2022	-16.925	32.7847	*P. canariensis*	933	*Laurus novocanariensis*	0.800	0.833	0.167	0.100
Fieldwork	MD16.2	Madeira	28/11/2022	-16.925	32.7847	*P. canariensis*	933	*Laurus novocanariensis*	1.000	0.765	0.235	0.538
Fieldwork	MD16.3	Madeira	28/11/2022	-16.925	32.7847	*P. canariensis*	933	*Laurus novocanariensis*	0.895	0.529	0.471	0.222
Fieldwork	MD16.4	Madeira	28/11/2022	-16.925	32.7847	*P. canariensis*	933	*Clethra arborea*	1.000	0.387	0.613	0.917
Fieldwork	MD16.5	Madeira	28/11/2022	-16.925	32.7847	*P. canariensis*	933	*Laurus novocanariensis*	1.000	0.972	0.028	0.286
Fieldwork	MD17.1	Madeira	30/11/2022	-16.9769	32.77	*P. canariensis*	1074	*Ocotea foetens*	0.727	1.000	0.000	0.125
Fieldwork	MD17.10	Madeira	30/11/2022	-16.9769	32.77	*P. canariensis*	1074	*Ocotea foetens*	0.224	0.364	0.636	0.000
Fieldwork	MD17.7	Madeira	30/11/2022	-16.9769	32.77	*P. canariensis*	1074	*Ocotea foetens*	1.000	0.130	0.870	0.000
Fieldwork	MD17.8	Madeira	30/11/2022	-16.9769	32.77	*P. canariensis*	1074	*Ocotea foetens*	0.885	1.000	0.000	0.087
Fieldwork	MD17.9	Madeira	30/11/2022	-16.9769	32.77	*P. canariensis*	1074	*Ocotea foetens*	1.000	1.000	0.000	0.000
Fieldwork	MD18.1	Madeira	30/11/2022	-16.9797	32.7775	*P. canariensis*	670	*Laurus novocanariensis*	0.760	0.921	0.079	0.229
Fieldwork	MD18.2	Madeira	30/11/2022	-16.9797	32.7775	*P. canariensis*	670	*Laurus novocanariensis*	0.700	0.667	0.333	0.143
Fieldwork	MD18.3	Madeira	30/11/2022	-16.9797	32.7775	*P. canariensis*	670	*Laurus novocanariensis*	0.950	1.000	0.000	0.474
Fieldwork	MD18.4	Madeira	30/11/2022	-16.9797	32.7775	*P. canariensis*	670	*Laurus novocanariensis*	0.733	1.000	0.000	0.000
Fieldwork	MD18.6	Madeira	30/11/2022	-16.9797	32.7775	*P. canariensis*	670	*Laurus novocanariensis*	0.766	1.000	0.000	0.020
Fieldwork	MD2.1	Madeira	29/11/2022	-17.1578	32.8253	*P. canariensis*	831	*Laurus novocanariensis*	0.912	0.290	0.710	0.556
Fieldwork	MD2.2	Madeira	29/11/2022	-17.1578	32.8253	*P. canariensis*	831	*Laurus novocanariensis*	1.000	0.962	0.038	0.280

Fieldwork	MD2.3	Madeira	29/11/2022	-17.1578	32.8253	*P. canariensis*	831	*Laurus novocanariensis*	0.280	1.000	0.000	0.000
Fieldwork	MD2.4	Madeira	29/11/2022	-17.1578	32.8253	*P. canariensis*	831	*Laurus novocanariensis*	0.788	0.327	0.673	0.471
Fieldwork	MD2.5	Madeira	29/11/2022	-17.1578	32.8253	*P. canariensis*	831	*Laurus novocanariensis*	1.000	1.000	0.000	0.069
Fieldwork	MD3.2	Madeira	28/11/2022	-16.8853	32.7347	*P. canariensis*	879	*Laurus novocanariensis*	1.000	0.370	0.630	0.235
Fieldwork	MD3.3	Madeira	28/11/2022	-16.8853	32.7347	*P. canariensis*	879	*Laurus novocanariensis*	0.955	0.238	0.762	0.000
Fieldwork	MD3.4	Madeira	28/11/2022	-16.8853	32.7347	*P. canariensis*	879	*Laurus novocanariensis*	1.000	0.286	0.714	0.750
Fieldwork	MD3.7	Madeira	28/11/2022	-16.8853	32.7347	*P. canariensis*	879	*Laurus novocanariensis*	1.000	0.512	0.488	0.091
Fieldwork	MD3.8	Madeira	28/11/2022	-16.8853	32.7347	*P. canariensis*	879	*Laurus novocanariensis*	1.000	0.250	0.750	0.000
Fieldwork	MD4.1	Madeira	28/11/2022	-16.8892	32.7408	*P. canariensis*	812	*Laurus novocanariensis*	0.429	0.833	0.167	0.200
Fieldwork	MD4.2	Madeira	28/11/2022	-16.8892	32.7408	*P. canariensis*	812	*Picconia excelsa*	1.000	1.000	0.000	0.200
Fieldwork	MD4.3	Madeira	28/11/2022	-16.8892	32.7408	*P. canariensis*	812	*Laurus novocanariensis*	0.643	0.167	0.833	0.000
Fieldwork	MD4.4	Madeira	28/11/2022	-16.8892	32.7408	*P. canariensis*	812	*Laurus novocanariensis*	1.000	1.000	0.000	0.000
Fieldwork	MD4.5	Madeira	28/11/2022	-16.8892	32.7408	*P. canariensis*	812	*Laurus novocanariensis*	0.595	0.960	0.040	0.000
Fieldwork	MD5.1	Madeira	02/12/2022	-16.9151	32.7421	*P. canariensis*	919	*Laurus novocanariensis*	1.000	1.000	0.000	0.333
Fieldwork	MD5.2	Madeira	02/12/2022	-16.9151	32.7421	*P. canariensis*	919	*Laurus novocanariensis*	1.000	0.941	0.059	0.063
Fieldwork	MD5.3	Madeira	02/12/2022	-16.9151	32.7421	*P. canariensis*	919	*Laurus novocanariensis*	0.194	1.000	0.000	0.000
Fieldwork	MD5.4	Madeira	02/12/2022	-16.9151	32.7421	*P. canariensis*	919	*Laurus novocanariensis*	1.000	0.575	0.425	0.217
Fieldwork	MD5.5	Madeira	02/12/2022	-16.9151	32.7421	*P. canariensis*	919	*Laurus novocanariensis*	1.000	1.000	0.000	0.286
Fieldwork	MD6.1	Madeira	02/12/2022	-16.9102	32.7436	*P. canariensis*	730	*Laurus novocanariensis*	0.571	0.750	0.250	0.667
Fieldwork	MD6.2	Madeira	02/12/2022	-16.9102	32.7436	*P. canariensis*	730	*Laurus novocanariensis*	1.000	0.015	0.985	0.000
Fieldwork	MD6.3	Madeira	02/12/2022	-16.9102	32.7436	*P. canariensis*	730	*Laurus novocanariensis*	1.000	0.679	0.321	0.278

Fieldwork	MD6.7	Madeira	02/12/2022	-16.9102	32.7436	*P. canariensis*	730	*Laurus novocanariensis*	1.000	1.000	0.000	1.000
Fieldwork	MD6.9	Madeira	02/12/2022	-16.9102	32.7436	*P. canariensis*	730	*Laurus novocanariensis*	1.000	0.947	0.053	0.056
Fieldwork	MD7.1	Madeira	02/12/2022	-16.9061	32.7453	*P. canariensis*	790	*Laurus novocanariensis*	1.000	0.881	0.119	0.027
Fieldwork	MD7.2	Madeira	02/12/2022	-16.9061	32.7453	*P. canariensis*	790	*Laurus novocanariensis*	1.000	0.254	0.746	0.133
Fieldwork	MD7.3	Madeira	02/12/2022	-16.9061	32.7453	*P. canariensis*	790	*Laurus novocanariensis*	1.000	0.043	0.957	0.000
Fieldwork	MD7.4	Madeira	02/12/2022	-16.9061	32.7453	*P. canariensis*	790	*Ocotea foetens*	1.000	0.358	0.642	0.211
Fieldwork	MD7.5	Madeira	02/12/2022	-16.9061	32.7453	*P. canariensis*	790	*Laurus novocanariensis*	1.000	0.583	0.417	0.357
Fieldwork	MD8.1	Madeira	01/12/2022	-17.0186	32.7536	*P. canariensis*	1007	*Laurus novocanariensis*	1.000	0.000	1.000	0.000
Fieldwork	MD8.6	Madeira	01/12/2022	-17.0186	32.7536	*P. canariensis*	1007	*Laurus novocanariensis*	1.000	1.000	0.000	0.235
Fieldwork	MD8.7	Madeira	01/12/2022	-17.0186	32.7536	*P. canariensis*	1007	*Hesperocyparis macrocarpa*	1.000	0.703	0.297	0.192
Fieldwork	MD8.8	Madeira	01/12/2022	-17.0186	32.7536	*P. canariensis*	1007	*Hesperocyparis macrocarpa*	1.000	1.000	0.000	0.167
Fieldwork	MD8.9	Madeira	01/12/2022	-17.0186	32.7536	*P. canariensis*	1007	*Laurus novocanariensis*	1.000	0.500	0.500	0.000
Fieldwork	MD9.1	Madeira	01/12/2022	-17.0153	32.7603	*P. canariensis*	810	*Laurus novocanariensis*	1.000	0.769	0.231	0.200
Fieldwork	MD9.2	Madeira	01/12/2022	-17.0153	32.7603	*P. canariensis*	810	*Laurus novocanariensis*	1.000	1.000	0.000	0.000
Fieldwork	MD9.3	Madeira	01/12/2022	-17.0153	32.7603	*P. canariensis*	810	*Laurus novocanariensis*	0.500	1.000	0.000	0.250
Fieldwork	MD9.5	Madeira	01/12/2022	-17.0153	32.7603	*P. canariensis*	810	*Laurus novocanariensis*	0.588	0.700	0.300	0.143
Fieldwork	MD9.6	Madeira	01/12/2022	-17.0153	32.7603	*P. canariensis*	810	*Ocotea foetens*	1.000	0.000	1.000	0.000
LISU Herbarium	254713	Madeira	23/10/2004	-16.8982	32.6673	*P. canariensis*	300	*NA*	0.231	0.667	0.333	0.500
LISU Herbarium	254402	Madeira	31/07/2003	-17.1233	32.7178	*P. canariensis*	700	*NA*	0.615	1.000	0.000	0.125
LISU Herbarium	260885	Madeira	17/11/2005	-16.9209	32.7301	*P. canariensis*	960	*Hebernia excelsea*	1.000	1.000	0.000	0.000
LISU Herbarium	261046	Madeira	24/07/2004	-16.9102	32.7303	*P. canariensis*	850	*Laurus novocanariensis*	0.100	0.000	1.000	0.000
LISU Herbarium	261217	Madeira	16/11/2005	-16.9104	32.7393	*P. canariensis*	850	*Ocotea foetens*	0.600	1.000	0.000	0.000

LISU Herbarium	254109	Madeira	24/07/2003	-16.9104	32.7393	*P. canariensis*	850	*NA*	0.565	1.000	0.000	0.154
LISU Herbarium	254656	Madeira	24/07/2004	-16.9104	32.7393	*P. canariensis*	750	*NA*	0.333	1.000	0.000	0.000
LISU Herbarium	254804	Madeira	24/07/2004	-16.9104	32.7393	*P. canariensis*	870	*NA*	0.211	0.500	0.500	0.000
LISU Herbarium	254633	Madeira	24/07/2004	-16.9104	32.7393	*P. canariensis*	870	*NA*	0.250	1.000	0.000	0.000
LISU Herbarium	254662	Madeira	24/07/2004	-16.9104	32.7393	*P. canariensis*	700	*NA*	0.733	0.909	0.091	0.000
LISU Herbarium	254137	Madeira	28/07/2003	-17.0173	32.7466	*P. canariensis*	900	*NA*	0.222	0.000	1.000	0.000
LISU Herbarium	250031	Madeira	09/08/2001	-17.0173	32.7466	*P. canariensis*	980	*NA*	0.308	1.000	0.000	0.000
LISU Herbarium	260941	Madeira	07/08/2007	-16.9213	32.7481	*P. canariensis*	950	*Persea indica*	0.500	1.000	0.000	0.000
LISU Herbarium	254187	Madeira	29/07/2003	-17.1348	32.7537	*P. canariensis*	1100	*NA*	0.500	1.000	0.000	0.000
LISU Herbarium	261194	Madeira	14/02/2015	-17.0068	32.7558	*P. canariensis*	600	*Ocotea foetens*	0.833	1.000	0.000	0.100
LISU Herbarium	261006	Madeira	20/07/2005	-16.9323	32.766	*P. canariensis*	1300	*Sambucus lanceolata*	1.000	0.923	0.077	0.000
LISU Herbarium	261234	Madeira	24/07/2006	-16.9752	32.7744	*P. canariensis*	620	*Laurus novocanariensis*	0.167	1.000	0.000	0.000
LISU Herbarium	254586	Madeira	28/06/2004	-16.9325	32.775	*P. canariensis*	650	*NA*	1.000	1.000	0.000	0.500
LISU Herbarium	254601	Madeira	28/06/2004	-16.9325	32.775	*P. canariensis*	600	*NA*	1.000	1.000	0.000	0.000
LISU Herbarium	261074	Madeira	03/08/2004	-16.9219	32.7752	*P. canariensis*	890	*Laurus novocanariensis*	1.000	0.318	0.682	0.000
LISU Herbarium	250037	Madeira	02/08/2002	-16.9219	32.7752	*P. canariensis*	900	*NA*	0.375	1.000	0.000	0.000
LISU Herbarium	261095	Madeira	05/08/2004	-17.0928	32.7815	*P. canariensis*	1000	*Laurus novocanariensis*	0.100	1.000	0.000	0.000
LISU Herbarium	260902	Madeira	23/11/2005	-16.9327	32.784	*P. canariensis*	770	*Laurus novocanariensis*	1.000	0.125	0.875	0.000
LISU Herbarium	254611	Madeira	28/06/2004	-16.9327	32.784	*P. canariensis*	700	*NA*	1.000	1.000	0.000	0.167
LISU Herbarium	260973	Madeira	19/07/2005	-17.1784	32.789	*P. canariensis*	1250	*Clethra arborea*	0.368	1.000	0.000	0.143
LISU Herbarium	261171	Madeira	22/07/2005	-17.0823	32.7907	*P. canariensis*	850	*Laurus novocanariensis*	0.667	0.400	0.600	0.000
LISU Herbarium	254673	Madeira	28/10/2004	-17.029	32.7915	*P. canariensis*	500	*NA*	0.000	0.000	0.000	0.000
LISU Herbarium	254692	Madeira	28/10/2004	-17.0183	32.7917	*P. canariensis*	1000	*NA*	0.286	1.000	0.000	0.250
LISU Herbarium	260925	Madeira	02/08/2007	-16.997	32.7921	*P. canariensis*	800	*Ocotea foetens*	1.000	0.200	0.800	0.000
LISU Herbarium	161856	Madeira	12/04/1988	-16.9329	32.7931	*P. canariensis*	500	*NA*	0.167	1.000	0.000	0.000
LISU Herbarium	161857	Madeira	12/04/1988	-16.9222	32.7932	*P. canariensis*	300	*NA*	0.417	1.000	0.000	0.000
LISU Herbarium	260991	Madeira	19/07/2005	-17.1679	32.7982	*P. canariensis*	600	*Laurus novocanariensis*	1.000	0.571	0.429	0.250

LISU Herbarium	261064	Madeira	28/07/2004	-17.1359	32.7988	*P. canariensis*	1200	*Laurus novocanariensis*	0.762	0.688	0.313	0.000
LISU Herbarium	261226	Madeira	24/11/2005	-17.1252	32.799	*P. canariensis*	1185	*Laurus novocanariensis*	1.000	0.200	0.800	0.000
LISU Herbarium	261057	Madeira	27/07/2004	-17.1146	32.7991	*P. canariensis*	510	*Persea indica*	1.000	1.000	0.000	0.111
LISU Herbarium	254698	Madeira	28/10/2004	-17.0185	32.8007	*P. canariensis*	1060	*NA*	0.286	1.000	0.000	0.000
LISU Herbarium	254707	Madeira	28/10/2004	-17.0078	32.8009	*P. canariensis*	750	*NA*	0.200	0.000	1.000	0.000
LISU Herbarium	261101	Madeira	15/07/2005	-17.2113	32.8245	*P. canariensis*	839	*Laurus novocanariensis*	1.000	1.000	0.000	0.118
LISU Herbarium	261109	Madeira	15/07/2005	-17.1902	32.8339	*P. canariensis*	836	*Laurus novocanariensis*	0.200	0.000	1.000	0.000
Fieldwork	S1.3	Iberian Peninsula	09/11/2022	-9.38857	38.7855	*P. canariensis*	482	*Pinus pinea*	0.000	0.000	0.000	0.000
Fieldwork	S1.6	Iberian Peninsula	09/11/2022	-9.38857	38.7855	*P. canariensis*	482	*Pinus pinea*	0.500	1.000	0.000	0.000
Fieldwork	S1.7	Iberian Peninsula	09/11/2022	-9.38857	38.7855	*P. canariensis*	482	*Pinus pinea*	0.044	1.000	0.000	0.000
Fieldwork	S1.8	Iberian Peninsula	09/11/2022	-9.38857	38.7855	*P. canariensis*	482	*Pinus pinea*	0.161	1.000	0.000	0.000
Fieldwork	S1.9	Iberian Peninsula	09/11/2022	-9.38857	38.7855	*P. canariensis*	482	*Pinus pinea*	0.873	0.246	0.754	0.000
Fieldwork	S4.10	Iberian Peninsula	09/11/2022	-9.46	38.7703	*P. canariensis*	428	*Cupressus lusitanica*	0.200	1.000	0.000	0.000
Fieldwork	S4.14	Iberian Peninsula	09/11/2022	-9.46	38.7703	*P. canariensis*	428	*Cupressus lusitanica*	0.188	1.000	0.000	0.000
Fieldwork	S4.6	Iberian Peninsula	09/11/2022	-9.46	38.7703	*P. canariensis*	428	*Cupressus lusitanica*	0.095	1.000	0.000	0.000
Fieldwork	S4.8	Iberian Peninsula	09/11/2022	-9.46	38.7703	*P. canariensis*	428	*Cupressus lusitanica*	0.324	1.000	0.000	0.000
Fieldwork	S4.9	Iberian Peninsula	09/11/2022	-9.46	38.7703	*P. canariensis*	428	*Cupressus lusitanica*	0.063	1.000	0.000	0.000
LISU Herbarium	264268	Iberian Peninsula	07/05/2014	-9.46214	38.7685	*P. canariensis*	450	*NA*	0.000	0.000	0.000	0.000
LISU Herbarium	160402	Iberian Peninsula		-9.39139	38.782	*P. canariensis*		*NA*	0.143	1.000	0.000	0.000
LISU Herbarium	148023	Iberian Peninsula	10/05/1972	-9.39139	38.782	*P. canariensis*		*NA*	0.000	0.000	0.000	0.000
LISU Herbarium	153619	Iberian Peninsula	08/05/1987	-9.39139	38.782	*P. canariensis*	400	*NA*	0.000	0.000	0.000	0.000
LISU Herbarium	256333	Iberian Peninsula	13/11/2013	-9.39139	38.782	*P. canariensis*		*NA*	0.219	1.000	0.000	0.000

LISU Herbarium	256366	Iberian Peninsula	17/12/2013	-9.43755	38.7844	*P. canariensis*		*Species unknown*	0.308	0.000	1.000	0.000
LISU Herbarium	256367	Iberian Peninsula	19/03/2014	-9.43755	38.7844	*P. canariensis*		*NA*	0.500	0.000	1.000	0.000
LISU Herbarium	256369	Iberian Peninsula	11/03/2014	-9.43755	38.7844	*P. canariensis*		*NA*	0.000	0.000	0.000	0.000
LISU Herbarium	255841	Iberian Peninsula	03/03/2011	-9.43755	38.7844	*P. canariensis*		*NA*	0.909	1.000	0.000	0.200
LISU Herbarium	255842	Iberian Peninsula	03/03/2011	-9.43755	38.7844	*P. canariensis*		*NA*	0.250	1.000	0.000	0.000
LISU Herbarium	256365	Iberian Peninsula	19/06/2013	-9.43755	38.7844	*P. canariensis*		*Species unknown*	0.500	1.000	0.000	0.000
LISU Herbarium	264310	Iberian Peninsula	04/06/2014	-9.43843	38.7845	*P. canariensis*	338	*NA*	0.308	1.000	0.000	0.000
LISU Herbarium	264281	Iberian Peninsula	16/05/2014	-9.39848	38.7847	*P. canariensis*	425	*NA*	0.167	1.000	0.000	0.000
LISU Herbarium	206857	Iberian Peninsula	30/09/2002	-9.40298	38.785	*P. canariensis*	114	*Species unknown*	0.316	1.000	0.000	0.333
LISU Herbarium	255843	Iberian Peninsula	25/03/2011	-9.39146	38.7851	*P. canariensis*		*NA*	0.294	1.000	0.000	0.000
LISU Herbarium	248239	Iberian Peninsula	25/03/2011	-9.38995	38.7852	*P. canariensis*		*NA*	0.167	0.750	0.250	0.333
LISU Herbarium	264079	Iberian Peninsula	25/03/2011	-9.38995	38.7852	*P. canariensis*		*NA*	0.750	0.000	1.000	0.000
LISU Herbarium	160400	Iberian Peninsula	23/10/1973	-9.39287	38.7888	*P. canariensis*		*NA*	1.000	0.000	1.000	0.000
LISU Herbarium	264361	Iberian Peninsula	19/06/2014	-9.38943	38.7905	*P. canariensis*	383	*NA*	0.333	1.000	0.000	0.000
LISU Herbarium	208236	Iberian Peninsula	12/02/2005	-9.38904	38.792	*P. canariensis*	400	*Species unknown*	0.000	0.000	0.000	0.000
LISU Herbarium	148035	Iberian Peninsula	27/04/1948	-9.38904	38.792	*P. canariensis*		*NA*	0.091	1.000	0.000	0.000
LISU Herbarium	148053	Iberian Peninsula	03/05/1941	-9.38904	38.792	*P. canariensis*		*NA*	0.130	1.000	0.000	0.000
LISU Herbarium	148031	Iberian Peninsula	16/03/1973	-9.38904	38.792	*P. canariensis*		*NA*	0.286	1.000	0.000	0.250
LISU Herbarium	153620	Iberian Peninsula	05/05/1987	-9.38904	38.792	*P. canariensis*		*NA*	0.100	1.000	0.000	0.000
LISU Herbarium	232690	Iberian Peninsula	30/10/2004	-9.39973	38.7943	*P. canariensis*		*NA*	0.895	0.000	1.000	0.000
LISU Herbarium	148027	Iberian Peninsula		-9.05173	39.1668	*P. canariensis*		*Species unknown*	0.000	0.000	0.000	0.000

LISU Herbarium	206026	Iberian Peninsula	22/02/2004	-8.75603	39.5578	*P. canariensis*	400	*Species unknown*	1.000	1.000	0.000	0.000
LISU Herbarium	247485	Iberian Peninsula	28/02/2011	-8.32141	39.9101	*P. canariensis*	260	*NA*	0.353	0.000	1.000	0.000
LISU Herbarium	205119	Iberian Peninsula	06/05/2003	-7.9187	40.2163	*P. canariensis*	492	*Species unknown*	0.000	0.000	0.000	0.000
LISU Herbarium	220090	Iberian Peninsula	26/06/2003	-8.28335	40.8637	*P. canariensis*	466	*Species unknown*	0.083	1.000	0.000	0.000
LISU Herbarium	249783	Iberian Peninsula	08/06/2010	-8.21872	41.7156	*P. canariensis*	392	*NA*	0.792	1.000	0.000	0.105
LISU Herbarium	263363	Iberian Peninsula	09/05/2012	-7.199	41.7833	*P. canariensis*	506	*NA*	0.067	1.000	0.000	0.000
LISU Herbarium	246895	Iberian Peninsula	01/07/2011	-6.98943	41.8133	*P. canariensis*	482	*NA*	0.000	0.000	0.000	0.000
E Herbarium	E00831506	Iberian Peninsula	31/05/2016	-5.54623	36.1027	*P. canariensis*	740	*Species unknown*	0.519	0.857	0.143	0.000
E Herbarium	E00831507	Iberian Peninsula	02/06/2016	-5.54479	36.5585	*P. canariensis*	535	*NA*	0.643	0.778	0.222	0.000
VAL Herbarium	1800	Iberian Peninsula	17/09/1981	-8.06129	43.412	*P. canariensis*	60	*Species unknown*	0.000	0.000	0.000	0.000
AZU Herbarium	5946	Azores		-28.1981	38.4358	*F. teneriffae*	1000	*Species unknown*	1.000	0.133	0.867	0.000
AZU Herbarium	6106	Azores		-28.1981	38.4358	*F. teneriffae*	940	*Species unknown*	0.000	0.000	0.000	0.000
AZU Herbarium	6032	Azores		-28.2176	38.4745	*F. teneriffae*	200	*NA*	0.000	0.000	0.000	0.000
AZU Herbarium	FP-20000701_FP085/02	Azores	01/07/2000	-28.3419	38.5068	*F. teneriffae*	770	*Species unknown*	1.000	0.842	0.158	0.063
AZU Herbarium	Ter cm 19970617-Lo1-A002.a	Azores	17/06/1997	-27.2645	38.7043	*F. teneriffae*	650	*NA*	0.350	1.000	0.000	0.000
AZU Herbarium	RG-20030731_"Geo293"_03	Azores	31/07/2003	-27.2037	38.7088	*F. teneriffae*	459	*Laurus azorica*	1.000	0.000	1.000	0.000
AZU Herbarium	RG-7298	Azores	24/07/2003	-27.2307	38.7255	*F. teneriffae*	574	*Eucalyptus globulus*	1.000	1.000	0.000	0.000
AZU Herbarium	RG7265	Azores	24/07/2003	-27.2307	38.7255	*F. teneriffae*	574	*Eucalyptus globulus*	1.000	1.000	0.000	0.000
AZU Herbarium	RG7328	Azores	24/07/2003	-27.2307	38.7255	*F. teneriffae*	574	*Eucalyptus globulus*	0.000	0.000	0.000	0.000
AZU Herbarium	FP_20071011_07	Azores	11/10/2007	-27.2152	38.7276	*F. teneriffae*	510	*NA*	0.091	1.000	0.000	0.000
AZU Herbarium	RG-5469	Azores	20/06/2003	-27.2272	38.734	*F. teneriffae*	650	*NA*	0.333	1.000	0.000	0.000
AZU Herbarium	RG-5977	Azores	04/07/2003	-27.2272	38.734	*F. teneriffae*	530	*Erica azorica*	1.000	0.538	0.462	0.000
AZU Herbarium	RG-5086	Azores	02/07/1999	-27.3133	38.7343	*F. teneriffae*	900	*NA*	1.000	0.600	0.400	0.000

AZU Herbarium	SB_HL3	Azores	10/10/2008	-27.3083	38.7358	*F. teneriffae*	890	*NA*	0.000	0.000	0.000	0.000
AZU Herbarium	s.n.	Azores	24/08/1997	-27.979	39.0301	*F. teneriffae*	300	*NA*	0.000	0.000	0.000	0.000
AZU Herbarium	RG-20050626_GRA002	Azores	26/06/2005	-28.0313	39.0398	*F. teneriffae*	340	*NA*	0.000	0.000	0.000	0.000
LISU Herbarium	164107	Azores	25/05/1980	-25.7966	37.8478	*F. teneriffae*		*NA*	0.500	0.500	0.500	0.000
LISU Herbarium	164104	Azores	22/06/1982	-28.2789	38.4285	*F. teneriffae*	300	*NA*	1.000	0.900	0.100	0.000
LISU Herbarium	261562	Azores	11/09/2007	-28.4236	38.4738	*F. teneriffae*	1400	*NA*	0.900	0.167	0.833	0.000
LISU Herbarium	164106	Azores	22/06/1982	-28.2908	38.4825	*F. teneriffae*	650	*Species unknown*	0.824	0.857	0.143	0.000
LISU Herbarium	164101	Azores	21/06/1982	-28.6518	38.5495	*F. teneriffae*	200	*Species unknown*	0.500	1.000	0.000	0.000
LISU Herbarium	225610	Azores	01/08/1997	-27.9273	38.5975	*F. teneriffae*		*NA*	1.000	0.000	1.000	0.000
LISU Herbarium	165791	Azores	15/05/1937	-27.961	38.5989	*F. teneriffae*		*NA*	1.000	1.000	0.000	0.000
LISU Herbarium	165723	Azores	22/04/1965	-28.7007	38.6059	*F. teneriffae*	620	*NA*	0.000	0.000	0.000	0.000
LISU Herbarium	165793	Azores	03/05/1937	-28.7691	38.6076	*F. teneriffae*		*NA*	0.821	1.000	0.000	0.000
LISU Herbarium	264901	Azores		-27.9745	38.6414	*F. teneriffae*	150	*NA*	0.000	0.000	0.000	0.000
LISU Herbarium	165721	Azores	05/09/1971	-27.3316	38.7493	*F. teneriffae*	800	*NA*	0.500	1.000	0.000	0.000
LISU Herbarium	165718	Azores	18/10/1971	-31.0914	39.6884	*F. teneriffae*	300	*NA*	0.333	1.000	0.000	0.000
LISU Herbarium	261569	Azores	13/09/2007	-16.8609	32.7891	*F. teneriffae*	346	*Species unknown*	1.000	0.909	0.091	0.000
LISU Herbarium	261576	Azores	13/09/2007	-16.8609	32.7891	*F. teneriffae*	346	*Species unknown*	1.000	0.818	0.182	0.000
LISU Herbarium	165795	Azores	21/06/1937	-25.0987	36.9638	*F. teneriffae*		*Species unknown*	1.000	0.625	0.375	0.000
LISU Herbarium	164136	Azores	30/05/1980	-25.312	37.7706	*F. teneriffae*		*Species unknown*	0.400	0.000	1.000	0.000
LISU Herbarium	165792	Azores	05/03/1986	-25.312	37.7706	*F. teneriffae*		*Species unknown*	1.000	0.000	1.000	0.000
LISU Herbarium	261581	Azores	10/09/2007	-28.3173	38.487	*F. teneriffae*	650	*Species unknown*	0.667	0.000	1.000	0.000
LISU Herbarium	261546	Azores	10/09/2007	-28.3173	38.487	*F. teneriffae*	650	*Species unknown*	1.000	0.400	0.600	0.000
LISU Herbarium	225609	Azores	24/06/1999	-27.8713	38.5725	*F. teneriffae*	500	*NA*	0.200	1.000	0.000	0.000
LISU Herbarium	164102	Azores	21/06/1982	-28.7258	38.5878	*F. teneriffae*	800	*Juniperus brevifolia*	0.800	1.000	0.000	0.000
LISU Herbarium	164103	Azores	21/06/1982	-28.7258	38.5878	*F. teneriffae*	800	*Juniperus brevifolia*	0.516	0.375	0.625	0.000

LISU Herbarium	165794	Azores	04/05/1937	-28.7258	38.5878	*F. teneriffae*		*Species unknown*	1.000	0.111	0.889	0.000
LISU Herbarium	165798	Azores	21/06/1982	-28.7258	38.5878	*F. teneriffae*	800	*Erica azorica*	1.000	0.542	0.458	0.077
LISU Herbarium	165799	Azores	21/06/1982	-28.7258	38.5878	*F. teneriffae*		*Erica azorica*	1.000	0.310	0.690	0.000
LISU Herbarium	261547	Azores	12/09/2007	-28.6965	38.5913	*F. teneriffae*	756	*NA*	0.067	1.000	0.000	0.000
LISU Herbarium	225607	Azores	01/08/1997	-27.9532	38.6016	*F. teneriffae*		*NA*	1.000	0.500	0.500	0.000
LISU Herbarium	225596	Azores	01/08/1997	-27.9804	38.6432	*F. teneriffae*		*NA*	1.000	0.000	1.000	0.000
LISU Herbarium	225606	Azores	01/07/1997	-28.0918	38.6545	*F. teneriffae*		*NA*	0.231	1.000	0.000	0.000
LISU Herbarium	225608	Azores	01/07/1997	-28.2654	38.7248	*F. teneriffae*		*NA*	0.800	1.000	0.000	0.000
LISU Herbarium	165796	Azores	18/12/1981	-27.2693	38.7379	*F. teneriffae*		*Species unknown*	0.500	0.000	1.000	0.000
E Herbarium	E00576944	Azores	04/04/1980	-27.2093	38.7291	*F. teneriffae*	370	*Species unknown*	1.000	0.571	0.429	0.000
C Herbarium	37705	Faroe Islands	23/07/1956	-6.75	61.4167	*F. teneriffae*		*NA*	0.000	0.000	0.000	0.000
C Herbarium	37704	Faroe Islands	23/07/1956	-6.75	61.4167	*F. teneriffae*		*NA*	0.000	0.000	0.000	0.000
C Herbarium	37701	Faroe Islands	21/07/1956	-6.8	61.45	*F. teneriffae*		*NA*	0.000	0.000	0.000	0.000
C Herbarium	37702	Faroe Islands	21/07/1956	-6.8	61.45	*F. teneriffae*		*NA*	0.000	0.000	0.000	0.000
C Herbarium	37703	Faroe Islands	03/08/1959	-6.81667	61.4667	*F. teneriffae*		*NA*	0.000	0.000	0.000	0.000
C Herbarium	30960	Faroe Islands	27/06/2015	-6.65	61.9833	*F. teneriffae*		*NA*	0.000	0.000	0.000	0.000
C Herbarium	31046	Faroe Islands	15/07/2000	-6.67166	62.0021	*F. teneriffae*		*NA*	0.000	0.000	0.000	0.000
C Herbarium	2060	Faroe Islands	09/07/1950	-7.60732	62.1108	*F. teneriffae*		*NA*	0.000	0.000	0.000	0.000
C Herbarium	2069	Faroe Islands	09/07/1950	-7.60732	62.1108	*F. teneriffae*		*NA*	0.000	0.000	0.000	0.000
C Herbarium	30800	Faroe Islands	02/07/2016	-7.13333	62.1667	*F. teneriffae*	233	*NA*	0.250	1.000	0.000	0.000
C Herbarium	2117	Faroe Islands	21/07/1950	-6.95596	62.3251	*F. teneriffae*		*NA*	1.000	0.000	1.000	0.000
C Herbarium	2122	Faroe Islands	21/07/1950	-6.95596	62.3251	*F. teneriffae*		*NA*	0.474	1.000	0.000	0.000
C Herbarium	37700	Faroe Islands	18/04/1905	-6.95996	62.3274	*F. teneriffae*		*NA*	0.000	0.000	0.000	0.000
N. Hodgetts (priv.collection)	11500	British Isles	02/04/2023	-5.99617	57.1042	*F. teneriffae*	10	*NA*	1.000	0.476	0.524	0.000
N. Hodgetts (priv.collection)	11501	British Isles	02/04/2023	-5.99126	57.1046	*F. teneriffae*	10	*NA*	0.000	0.000	0.000	0.000
N. Hodgetts (priv.collection)	11499	British Isles	02/04/2023	-5.99005	57.1048	*F. teneriffae*	10	*NA*	0.000	0.000	0.000	0.000
N. Hodgetts (priv.collection)	11498	British Isles	02/04/2023	-5.98739	57.1049	*F. teneriffae*	10	*NA*	0.000	0.000	0.000	0.000

E Herbarium	J.A.Paton Herbarium 6028	British Isles	18/04/1969	-2.05083	49.2173	*F. teneriffae*		*NA*	0.100	1.000	0.000	0.000
E Herbarium	J.A.Paton Herbarium 6020	British Isles	17/04/1961	-5.23082	49.9756	*F. teneriffae*		*NA*	1.000	1.000	0.000	0.000
E Herbarium	J.A.Paton Herbarium 6021	British Isles	13/04/1970	-5.69406	50.0795	*F. teneriffae*		*Species unknown*	0.480	1.000	0.000	0.000
E Herbarium	Herbarium C.C. Townsed	British Isles	27/08/1967	-4.0944	53.0093	*F. teneriffae*		*NA*	0.000	0.000	0.000	0.000
E Herbarium	272	British Isles		-4.07411	53.0691	*F. teneriffae*		*NA*	0.000	0.000	0.000	0.000
E Herbarium	82	British Isles		-1.4785	53.9984	*F. teneriffae*		*Species unknown*	1.000	1.000	0.000	0.000
E Herbarium	J.A.Paton Herbarium 6896	British Isles	24/04/1968	-4.60666	54.0937	*F. teneriffae*		*Species unknown*	0.923	1.000	0.000	0.000
E Herbarium	E00427988	British Isles	19/09/1969	-3.21278	54.4578	*F. teneriffae*		*NA*	0.032	1.000	0.000	0.000
E Herbarium	E00721385	British Isles	25/06/1957	-3.26663	54.5424	*F. teneriffae*		*Species unknown*	0.048	1.000	0.000	0.000
E Herbarium	E00213543	British Isles	15/09/1965	-4.43777	54.8697	*F. teneriffae*		*Species unknown*	1.000	1.000	0.000	0.000
E Herbarium	6005	British Isles	26/02/2000	-5.11626	55.2531	*F. teneriffae*	10	*NA*	0.333	1.000	0.000	0.000
E Herbarium	E00418172	British Isles		-5.79519	55.3004	*F. teneriffae*		*Species unknown*	0.067	1.000	0.000	0.000
E Herbarium	45632	British Isles	30/07/2018	-5.11275	55.5513	*F. teneriffae*	5	*NA*	0.368	1.000	0.000	0.000
E Herbarium	E00729019	British Isles	16/09/1982	-5.51297	55.5951	*F. teneriffae*		*NA*	0.200	1.000	0.000	0.000
E Herbarium	668	British Isles	31/03/1969	-5.34975	55.6708	*F. teneriffae*		*NA*	0.867	0.000	1.000	0.000
E Herbarium	19628	British Isles	27/05/1991	-5.74666	55.691	*F. teneriffae*	30	*Species unknown*	0.600	1.000	0.000	0.000
E Herbarium	E00728945	British Isles	08/08/1982	-5.44759	55.7223	*F. teneriffae*		*NA*	0.615	0.750	0.250	0.000
E Herbarium	27876	British Isles	26/07/1998	-6.10969	55.8459	*F. teneriffae*	10	*NA*	0.048	1.000	0.000	0.000
E Herbarium	E00418170	British Isles		-4.93381	56.1361	*F. teneriffae*		*NA*	0.308	1.000	0.000	0.000
E Herbarium	E00213481	British Isles	03/09/2006	-2.55702	56.1868	*F. teneriffae*		*NA*	0.286	1.000	0.000	0.000
E Herbarium	M.F.V Corley Herbarium	British Isles	08/09/1965	-5.63842	56.2378	*F. teneriffae*		*NA*	0.875	0.000	1.000	0.000
E Herbarium	M.F.V Corley Herbarium	British Isles	20/06/1965	-5.47519	56.2615	*F. teneriffae*		*Species unknown*	0.273	1.000	0.000	0.000
E Herbarium	M.F.V Corley Herbarium	British Isles		-5.47519	56.2615	*F. teneriffae*		*NA*	0.050	1.000	0.000	0.000
E Herbarium	M.F.V Corley Herbarium	British Isles	25/05/1965	-5.47519	56.2615	*F. teneriffae*		*NA*	0.000	0.000	0.000	0.000
E Herbarium	46962	British Isles	16/09/2020	-6.2318	56.3013	*F. teneriffae*	10	*Species unknown*	0.259	1.000	0.000	0.000
E Herbarium	E00350362	British Isles		-6.19745	56.3022	*F. teneriffae*		*NA*	0.913	1.000	0.000	0.000

E Herbarium	E00418171	British Isles		-5.97163	56.3296	*F. teneriffae*		*Species unknown*	0.067	1.000	0.000	0.000		
E Herbarium	46970	British Isles	18/09/2020	-6.23689	56.333	*F. teneriffae*	5	*NA*	0.000	0.000	0.000	0.000		
E Herbarium	E00669506	British Isles	24/06/2012	-6.2053	56.4807	*F. teneriffae*	5	*NA*	1.000	1.000	0.000	0.000		
E Herbarium	E00005355	British Isles	31/08/1976	-5.11292	56.6799	*F. teneriffae*		*NA*	1.000	1.000	0.000	0.000		
E Herbarium	16145	British Isles	26/05/1989	-6.00038	56.7179	*F. teneriffae*	5	*NA*	1.000	0.267	0.733	0.000		
E Herbarium	V. Schiffner (1003)	British Isles	03/05/1901	-5.79277	56.7884	*F. teneriffae*		*NA*	1.000	0.714	0.286	0.050		
E Herbarium	V. Schiffner (1002)	British Isles	23/05/1901	-5.79277	56.7884	*F. teneriffae*		*NA*	0.091	0.500	0.500	0.000		
E Herbarium	1897	British Isles	14/04/1972	-5.76779	56.7908	*F. teneriffae*		*NA*	0.833	1.000	0.000	0.000		
E Herbarium	25407	British Isles	19/06/1994	-7.63947	56.8063	*F. teneriffae*	130	*Species unknown*	0.214	1.000	0.000	0.000		
E Herbarium	37829	British Isles	31/05/2008	-7.63923	56.8084	*F. teneriffae*	65	*NA*	0.033	1.000	0.000	0.000		
E Herbarium	43823	British Isles	05/07/2015	-6.13114	56.8779	*F. teneriffae*	3	*NA*	0.038	1.000	0.000	0.000		
E Herbarium	E00452794	British Isles		-6.14969	56.8996	*F. teneriffae*		*Species unknown*	0.273	1.000	0.000	0.000		
E Herbarium	1875	British Isles	11/04/1972	-5.84296	56.9074	*F. teneriffae*		*NA*	0.750	1.000	0.000	0.000		
E Herbarium	31610	British Isles	13/10/2002	-5.82947	56.9123	*F. teneriffae*		*NA*	0.769	1.000	0.000	0.000		
E Herbarium	E00669507	British Isles	22/06/2012	-7.54268	56.9277	*F. teneriffae*	100	*NA*	0.067	1.000	0.000	0.000		
E Herbarium	25407	British Isles	19/06/1994	-7.63947	56.8063	*F. teneriffae*	130	*NA*	0.107	1.000	0.000	0.000		
E Herbarium	16233	British Isles	22/06/1989	-6.29535	56.965	*F. teneriffae*	200	*Species unknown*	0.000	0.000	0.000	0.000		
E Herbarium	33272	British Isles	29/06/2004	-6.36135	56.9702	*F. teneriffae*	19	*NA*	0.000	0.000	0.000	0.000		
E Herbarium	Herbarium A.C.Crundwell	British Isles	21/05/1945	-5.82085	56.9711	*F. teneriffae*		*NA*	0.000	0.000	0.000	0.000		
E Herbarium	S402 (RBG648 Germplasm project)	British Isles	02/07/2004	-6.35665	57.0506	*F. teneriffae*	40	*NA*	0.571	1.000	0.000	0.000		
E Herbarium	E00323714	British Isles		-6.53064	57.063	*F. teneriffae*		*Species unknown*	0.036	1.000	0.000	0.000		
E Herbarium	1348	British Isles	13/04/1971	-5.65872	57.0704	*F. teneriffae*	20	*NA*	0.909	1.000	0.000	0.000		
E Herbarium	E00110373	British Isles	01/08/2005	-5.6582	57.0763	*F. teneriffae*		*NA*	1.000	1.000	0.000	0.000		
E Herbarium	1414	British Isles	16/04/1971	-5.63319	57.202	*F. teneriffae*		*NA*	1.000	1.000	0.000	0.000		
E Herbarium	Herbarium C.C. Townsed	British Isles	19/08/1968	-6.19368	57.2032	*F. teneriffae*		*NA*	0.130	0.333	0.667	0.000		
E Herbarium	20190	British Isles	29/07/1991	-5.74552	57.2738	*F. teneriffae*	10	*NA*	0.444	1.000	0.000	0.000		
E Herbarium	20197	British Isles	29/07/1991	-5.72676	57.283	*F. teneriffae*	5	*NA*	0.259	1.000	0.000	0.000		

E Herbarium	E00391089	British Isles	03/08/1968	-6.56939	57.348	*F. teneriffae*		*NA*	1.000	1.000	0.000	0.000
E Herbarium	E00391085	British Isles	09/08/1968	-6.56939	57.348	*F. teneriffae*		*NA*	0.500	0.800	0.200	0.000
E Herbarium	E00391087	British Isles	24/06/1967	-6.56939	57.348	*F. teneriffae*		*NA*	1.000	0.438	0.563	0.000
E Herbarium	E00391088	British Isles	10/08/1966	-6.2026	57.3929	*F. teneriffae*		*NA*	0.000	0.000	0.000	0.000
E Herbarium	1157	British Isles	24/06/1970	-6.58975	57.4486	*F. teneriffae*		*NA*	0.500	0.300	0.700	0.000
E Herbarium	E00391086	British Isles	15/06/1962	-6.61847	57.4752	*F. teneriffae*		*NA*	0.167	1.000	0.000	0.000
E Herbarium	E00213086	British Isles	12/07/1966	-6.18632	57.6193	*F. teneriffae*		*Species unknown*	1.000	1.000	0.000	0.000
E Herbarium	38460	British Isles	16/06/2009	-6.26405	57.6449	*F. teneriffae*	300	*NA*	0.222	1.000	0.000	0.000
E Herbarium	47152	British Isles	20/06/2021	-5.80213	57.6544	*F. teneriffae*	20	*Species unknown*	0.040	1.000	0.000	0.000
E Herbarium	13751	British Isles	02/08/1986	-5.50154	57.6738	*F. teneriffae*	50	*NA*	1.000	1.000	0.000	0.033
E Herbarium	Herbarium C.C. Townsed	British Isles	19/08/1968	-6.34333	57.6934	*F. teneriffae*		*NA*	0.241	1.000	0.000	0.000
E Herbarium	19663	British Isles	08/06/1991	-7.17505	57.7162	*F. teneriffae*	25	*NA*	0.100	1.000	0.000	0.000
E Herbarium	19698	British Isles	10/06/1991	-8.56808	57.8123	*F. teneriffae*	40	*NA*	0.000	0.000	0.000	0.000
E Herbarium	39334	British Isles	21/06/2010	-8.56809	57.8128	*F. teneriffae*	147	*NA*	0.000	0.000	0.000	0.000
E Herbarium	M.F.V Corley Herbarium	British Isles	01/10/1967	-6.9452	57.8849	*F. teneriffae*		*NA*	0.083	1.000	0.000	0.000
E Herbarium	31947	British Isles	02/06/2023	-5.14904	57.8966	*F. teneriffae*	5	*NA*	0.259	1.000	0.000	0.000
E Herbarium	9542	British Isles	04/06/1981	-6.94682	57.901	*F. teneriffae*	2	*NA*	0.000	0.000	0.000	0.000
E Herbarium	9478	British Isles	01/06/1981	-7.10252	57.9961	*F. teneriffae*	20	*NA*	0.033	1.000	0.000	0.000
E Herbarium	3346	British Isles	15/04/1974	-4.70105	58.466	*F. teneriffae*		*NA*	0.875	1.000	0.000	0.071
E Herbarium	3542	British Isles	26/06/1974	-4.70105	58.466	*F. teneriffae*		*NA*	0.714	1.000	0.000	0.200
E Herbarium	2319	British Isles	18/04/1973	-4.70105	58.466	*F. teneriffae*		*NA*	1.000	1.000	0.000	0.000
E Herbarium	24628	British Isles	30/07/1993	-1.28329	59.8697	*F. teneriffae*	70	*Species unknown*	0.000	0.000	0.000	0.000
E Herbarium	E00427993	British Isles				*F. teneriffae*		*NA*	0.000	0.000	0.000	0.000
E Herbarium	36367	British Isles	11/10/2006	-16.2796	28.5316	*F. teneriffae*	15	*NA*	1.000	1.000	0.000	0.000
E Herbarium	15456	British Isles	01/01/1989	-10.0759	51.6623	*F. teneriffae*	20	*NA*	0.067	1.000	0.000	0.000
E Herbarium	28530	British Isles	16/07/1999	-9.54344	51.7361	*F. teneriffae*	10	*Species unknown*	1.000	1.000	0.000	0.000
E Herbarium	A.C. Crundwell Herbarium	British Isles	01/09/1979	-9.55975	51.7535	*F. teneriffae*		*NA*	0.800	0.500	0.500	0.000
E Herbarium	11829	British Isles	05/08/1983	-10.54	51.7713	*F. teneriffae*	100	*NA*	0.000	0.000	0.000	0.000

E Herbarium	23176	British Isles	28/12/1992	-9.37412	51.9605	*F. teneriffae*	90	*NA*	0.952	0.000	1.000	0.000
E Herbarium	M.F.V Corley Herbarium	British Isles	25/03/1966	-8.99306	52.0025	*F. teneriffae*		*Species unknown*	0.381	0.000	1.000	0.000
E Herbarium	E00427991	British Isles	20/08/1965	-9.50501	52.0032	*F. teneriffae*		*Species unknown*	0.000	0.000	0.000	0.000
E Herbarium	E00427989	British Isles	20/08/1965	-9.50501	52.0032	*F. teneriffae*		*NA*	0.478	0.000	1.000	0.000
E Herbarium	E00427990	British Isles	20/08/1965	-9.5064	52.0166	*F. teneriffae*		*NA*	0.074	1.000	0.000	0.000
E Herbarium	11627	British Isles	21/07/1983	-9.59398	52.0421	*F. teneriffae*	45	*NA*	1.000	0.000	1.000	0.000
E Herbarium	E00323076	British Isles	03/09/1951	-9.86508	52.0448	*F. teneriffae*		*NA*	1.000	0.500	0.500	0.000
E Herbarium	E00427994	British Isles	22/08/1965	-10.2904	52.1427	*F. teneriffae*		*NA*	0.000	0.000	0.000	0.000
E Herbarium	J.A.Paton Herbarium 6027	British Isles	02/08/1966	-7.52151	52.2512	*F. teneriffae*		*NA*	0.000	0.000	0.000	0.000
E Herbarium	J.A.Paton Herbarium 6026	British Isles	01/06/1968	-7.07221	52.2748	*F. teneriffae*		*NA*	0.765	0.769	0.231	0.100
E Herbarium	M.F.V Corley Herbarium	British Isles	01/04/1966	-8.47092	52.5914	*F. teneriffae*		*NA*	0.000	0.000	0.000	0.000
E Herbarium	25454	British Isles	19/07/1994	-9.35103	53.0703	*F. teneriffae*	195	*Species unknown*	1.000	1.000	0.000	0.000
E Herbarium	25501	British Isles	22/07/1994	-9.92003	53.4071	*F. teneriffae*	5	*NA*	0.152	1.000	0.000	0.000
E Herbarium	32008	British Isles	03/07/2003	-9.78576	53.4647	*F. teneriffae*	20	*Species unknown*	0.000	0.000	0.000	0.000
E Herbarium	31496	British Isles	15/08/2002	-9.88076	53.4681	*F. teneriffae*	20	*NA*	0.882	1.000	0.000	0.133
E Herbarium	E00723460	British Isles	05/07/1963	-10.0375	53.4887	*F. teneriffae*		*Species unknown*	0.000	0.000	0.000	0.000
E Herbarium	E00723458	British Isles	12/07/1965	-9.49023	53.5079	*F. teneriffae*		*NA*	1.000	1.000	0.000	0.000
E Herbarium	25529	British Isles	23/07/1994	-9.85855	53.5227	*F. teneriffae*	500	*Species unknown*	0.000	0.000	0.000	0.000
E Herbarium	36374	British Isles	12/05/2006	-10.1541	53.5262	*F. teneriffae*	5	*Species unknown*	0.048	1.000	0.000	0.000
E Herbarium	E00888340	British Isles	22/08/2017	-10.1598	53.5336	*F. teneriffae*	10	*Species unknown*	0.000	0.000	0.000	0.000
E Herbarium	33415	British Isles	20/07/2004	-10.1698	53.5365	*F. teneriffae*	8	*NA*	0.091	1.000	0.000	0.000
E Herbarium	44006	British Isles	13/08/2015	-10.1645	53.5371	*F. teneriffae*	10	*Species unknown*	0.375	1.000	0.000	0.000
E Herbarium	E00997454	British Isles	25/07/2019	-10.165	53.549	*F. teneriffae*	3	*NA*	0.032	1.000	0.000	0.000
E Herbarium	36808	British Isles	07/07/2007	-10.2281	53.5503	*F. teneriffae*	22	*NA*	1.000	1.000	0.000	0.000
E Herbarium	27308	British Isles	26/08/1997	-9.9449	53.5507	*F. teneriffae*	50	*NA*	1.000	1.000	0.000	0.000
E Herbarium	13878	British Isles	11/08/1986	-10.1326	53.5571	*F. teneriffae*	2	*NA*	0.000	0.000	0.000	0.000

E Herbarium	28445	British Isles	07/07/1999	-10.1142	53.5576	*F. teneriffae*	15	*NA*	0.000	0.000	0.000	0.000
E Herbarium	E00723459	British Isles	24/06/1966	-9.87882	53.5615	*F. teneriffae*		*NA*	1.000	0.000	1.000	0.000
E Herbarium	36777	British Isles	04/07/2007	-9.88811	53.5623	*F. teneriffae*	123	*NA*	1.000	0.893	0.107	0.120
E Herbarium	31528	British Isles	16/08/2002	-9.63431	53.6016	*F. teneriffae*	120	*NA*	0.000	0.000	0.000	0.000
E Herbarium	E00108705	British Isles	02/07/2001	-10.2086	53.6137	*F. teneriffae*	2	*NA*	0.000	0.000	0.000	0.000
E Herbarium	14461	British Isles	13/08/1987	-9.63913	53.7655	*F. teneriffae*	270	*NA*	0.050	1.000	0.000	0.000
E Herbarium	E00669505	British Isles	09/07/2012	-9.77008	53.7752	*F. teneriffae*	5	*NA*	1.000	1.000	0.000	0.000
E Herbarium	44016	British Isles	17/08/2015	-9.94683	53.8831	*F. teneriffae*	146	*NA*	0.107	1.000	0.000	0.000
E Herbarium	E00213085	British Isles	03/08/1965	-9.78879	53.9101	*F. teneriffae*		*NA*	0.000	0.000	0.000	0.000
E Herbarium	E00213087	British Isles	06/08/1965	-9.78879	53.9101	*F. teneriffae*		*Species unknown*	0.750	1.000	0.000	0.000
E Herbarium	14438	British Isles	11/08/1987	-9.95583	53.932	*F. teneriffae*	60	*Species unknown*	1.000	0.667	0.333	0.000
E Herbarium	14291	British Isles	07/08/1987	-10.122	53.9718	*F. teneriffae*	20	*Species unknown*	0.333	1.000	0.000	0.000
E Herbarium	E00427985	British Isles	08/06/1967	-8.44865	54.2742	*F. teneriffae*		*NA*	0.464	1.000	0.000	0.000
E Herbarium	20246	British Isles	07/08/1991	-8.3585	55.0886	*F. teneriffae*	5	*NA*	0.000	0.000	0.000	0.000
E Herbarium	Herbarium C.C. Townsed 78/363	Canary Islands	04/04/1978	-16.3791	28.4471	*F. teneriffae*	1080	*Species unknown*	1.000	1.000	0.000	0.200
E Herbarium	A.C. Crundwell Herbarium 57	Canary Islands	08/04/1975	-16.2957	28.5231	*F. teneriffae*		*Species unknown*	1.000	0.800	0.200	0.000
E Herbarium	5783	Canary Islands	02/04/1977	-16.2796	28.5316	*F. teneriffae*	950	*Species unknown*	0.889	0.188	0.813	0.000
E Herbarium	5776	Canary Islands	02/04/1977	-16.2796	28.5316	*F. teneriffae*	950	*Species unknown*	1.000	0.000	1.000	0.000
E Herbarium	5760	Canary Islands	02/04/1977	-16.2796	28.5316	*F. teneriffae*	950	*Species unknown*	1.000	0.370	0.630	0.000
E Herbarium	5740	Canary Islands	02/04/1977	-16.2796	28.5316	*F. teneriffae*	950	*Species unknown*	1.000	0.625	0.375	0.050
E Herbarium	E00576943	Canary Islands	11/04/1974	-16.2796	28.5316	*F. teneriffae*		*Species unknown*	1.000	0.455	0.545	0.000
E Herbarium	Herbarium C.C. Townsed 78/159	Canary Islands	26/03/1978	-16.2796	28.5316	*F. teneriffae*	970	*Species unknown*	1.000	0.407	0.593	0.000
E Herbarium	E00576951	Canary Islands	22/03/1977	-16.2642	28.5334	*F. teneriffae*	1000	*NA*	0.688	0.136	0.864	0.000
E Herbarium	Herbarium C.C. Townsed 78/172	Canary Islands	26/03/1978	-16.2642	28.5334	*F. teneriffae*	990	*Species unknown*	1.000	0.000	1.000	0.000

E Herbarium	A.C. Crundwell Herbarium 143	Canary Islands	25/03/1976	-16.2642	28.5334	*F. teneriffae*	1000	*Species unknown*	1.000	0.400	0.600	0.000
E Herbarium	E00576947	Canary Islands	17/07/1957	-16.2291	28.543	*F. teneriffae*		*Species unknown*	0.000	0.000	0.000	0.000
E Herbarium	E00576950	Canary Islands	25/03/1976	-16.2068	28.549	*F. teneriffae*	700	*NA*	1.000	1.000	0.000	0.000
E Herbarium	A.C. Crundwell Herbarium 65	Canary Islands	08/04/1975	-16.2068	28.549	*F. teneriffae*	670	*Species unknown*	0.737	0.429	0.571	0.000
TFC Herbarium	1482	Canary Islands	02/07/1985	-16.8364	28.3227	*F. teneriffae*	873	*Erica arborea*	1.000	0.500	0.500	0.083
TFC Herbarium	1499	Canary Islands	02/07/1985	-16.8364	28.3227	*F. teneriffae*	873	*Erica arborea*	0.846	0.364	0.636	0.000
TFC Herbarium	1572	Canary Islands	02/07/1985	-16.8364	28.3227	*F. teneriffae*	873	*Erica arborea*	0.947	0.556	0.444	0.100
TFC Herbarium	1637	Canary Islands	02/11/1986	-16.8221	28.3374	*F. teneriffae*	680	*Pinus radiata*	1.000	1.000	0.000	0.000
TFC Herbarium	1552	Canary Islands	26/02/1986	-16.8236	28.338	*F. teneriffae*	760	*Myrica Faya*	1.000	0.500	0.500	0.200
TFC Herbarium	1567	Canary Islands	29/04/1985	-16.8236	28.338	*F. teneriffae*	760	*Erica arborea*	1.000	0.235	0.765	0.500
TFC Herbarium	1666	Canary Islands	15/12/1986	-16.8236	28.338	*F. teneriffae*	760	*Arbutus canariensis*	0.714	0.500	0.500	0.000
TFC Herbarium	1619	Canary Islands	27/05/1985	-16.8236	28.338	*F. teneriffae*	760	*Persea indica*	0.600	0.333	0.667	0.000
TFC Herbarium	1599	Canary Islands	27/10/1986	-16.8236	28.338	*F. teneriffae*	760	*Erica arborea*	0.000	0.000	0.000	0.000
TFC Herbarium	6308	Canary Islands		-16.7633	28.3429	*F. teneriffae*	825	*NA*	0.667	0.667	0.333	0.000
TFC Herbarium	3792	Canary Islands	16/04/1986	-16.5824	28.3458	*F. teneriffae*	1100	*Pinus radiata*	1.000	1.000	0.000	0.000
TFC Herbarium	3808	Canary Islands	17/04/1986	-16.5824	28.3458	*F. teneriffae*	1100	*Myrica faya*	0.750	0.333	0.667	0.000
TFC Herbarium	3715	Canary Islands	24/02/1986	-16.4091	28.4221	*F. teneriffae*	1250	*Species unknown*	1.000	0.000	1.000	0.000
TFC Herbarium	3001	Canary Islands	18/06/1985	-16.3921	28.4307	*F. teneriffae*	1270	*Adenocarpus foliosus*	0.750	0.778	0.222	0.000
TFC Herbarium	3004	Canary Islands	18/06/1985	-16.3921	28.4307	*F. teneriffae*	1270	*Erica arborea*	1.000	0.417	0.583	0.000
TFC Herbarium	3877	Canary Islands	05/05/1986	-16.3933	28.4439	*F. teneriffae*	1140	*Myrica faya*	0.800	0.563	0.438	0.111
TFC Herbarium	3879	Canary Islands	05/05/1986	-16.3933	28.4439	*F. teneriffae*	1140	*Species unknown*	0.500	1.000	0.000	0.000
TFC Herbarium	3883	Canary Islands	05/05/1986	-16.3933	28.4439	*F. teneriffae*	1140	*Pinus radiata*	1.000	0.000	1.000	0.000
TFC Herbarium	3885	Canary Islands	05/05/1986	-16.3933	28.4439	*F. teneriffae*	1140	*Laurus azorica*	0.667	0.500	0.500	0.000
TFC Herbarium	3897	Canary Islands	05/05/1986	-16.3933	28.4439	*F. teneriffae*	1140	*Viburnum tinus*	0.867	0.077	0.923	0.000
TFC Herbarium	2511	Canary Islands	11/06/1984	-16.4037	28.449	*F. teneriffae*	1000	*Erica arborea*	1.000	0.071	0.929	0.000
TFC Herbarium	2529	Canary Islands	11/06/1984	-16.4037	28.449	*F. teneriffae*	1000	*Erica arborea*	0.286	0.500	0.500	0.000
TFC Herbarium	2519	Canary Islands	11/06/1984	-16.4037	28.449	*F. teneriffae*	1000	*Erica arborea*	0.545	1.000	0.000	0.167

TFC Herbarium	2318	Canary Islands	21/11/1983	-16.4051	28.4554	*F. teneriffae*	920	*Laurus azorica*	0.533	0.125	0.875	0.000
TFC Herbarium	2320	Canary Islands	21/11/1985	-16.4051	28.4554	*F. teneriffae*	920	*Laurus azorica*	1.000	0.000	1.000	0.000
TFC Herbarium	2343	Canary Islands	05/12/1983	-16.4051	28.4554	*F. teneriffae*	920	*Laurus azorica*	1.000	0.923	0.077	0.000
TFC Herbarium	2822	Canary Islands	26/11/1984	-16.4051	28.4554	*F. teneriffae*	920	*Myrica faya*	0.077	0.000	1.000	0.000
TFC Herbarium	2306	Canary Islands	13/02/1984	-16.4021	28.4572	*F. teneriffae*	860	*NA*	0.391	0.556	0.444	0.000
TFC Herbarium	3956	Canary Islands	07/05/1986	-16.4021	28.4572	*F. teneriffae*	860	*Erica arborea*	0.833	1.000	0.000	0.000
TFC Herbarium	10933	Canary Islands	10/04/1999	-16.2673	28.5248	*F. teneriffae*	920	*Laurus azorica*	1.000	1.000	0.000	0.000
TFC Herbarium	1253	Canary Islands	10/04/1984	-16.2738	28.5275	*F. teneriffae*	750	*NA*	0.231	0.500	0.500	0.000
TFC Herbarium	1569	Canary Islands	18/06/1986			*F. teneriffae*		*Laurus azorica*	1.000	0.000	1.000	0.000
TFC Herbarium	3505	Canary Islands	09/12/1985			*F. teneriffae*		*Pinus canariensis*	0.957	0.864	0.136	0.053
TFC Herbarium	3034	Canary Islands	26/07/1985			*F. teneriffae*		*NA*	0.882	1.000	0.000	0.000
TFC Herbarium	3804	Canary Islands	17/04/1986			*F. teneriffae*		*NA*	1.000	0.077	0.923	0.000
TFC Herbarium	3800	Canary Islands	17/04/1986			*F. teneriffae*		*Pinus radiata*	1.000	1.000	0.000	0.067
E Herbarium	Herbarium C.C. Townsed 78/361	Canary Islands	04/04/1978	-16.3791	28.4471	*F. teneriffae*	1080	*Species unknown*	1.000	0.000	1.000	0.000
Fieldwork	13.2	Canary Islands	18/10/2022	-16.3064	28.5369	*F. teneriffae*	809	*Erica platycodon*	1.000	0.339	0.661	0.000
Fieldwork	13.3	Canary Islands	18/10/2022	-16.3064	28.5369	*F. teneriffae*	809	*Ilex perado*	1.000	0.000	1.000	0.000
Fieldwork	13.7	Canary Islands	18/10/2022	-16.3064	28.5369	*F. teneriffae*	809	*Prunus lusitanica*	1.000	0.902	0.098	0.000
Fieldwork	13.8	Canary Islands	18/10/2022	-16.3064	28.5369	*F. teneriffae*	809	*Erica platycodon*	1.000	0.540	0.460	0.000
Fieldwork	13.9	Canary Islands	18/10/2022	-16.3064	28.5369	*F. teneriffae*	809	*Erica platycodon*	0.926	0.200	0.800	0.000
Fieldwork	14.10	Canary Islands	18/10/2022	-16.2956	28.5295	*F. teneriffae*	850	*Erica platycodon*	1.000	0.478	0.522	0.000
Fieldwork	14.11	Canary Islands	18/10/2022	-16.2956	28.5295	*F. teneriffae*	850	*Erica platycodon*	1.000	0.786	0.214	0.045
Fieldwork	14.12	Canary Islands	18/10/2022	-16.2956	28.5295	*F. teneriffae*	850	*Erica platycodon*	1.000	0.563	0.438	0.185
Fieldwork	14.13	Canary Islands	18/10/2022	-16.2956	28.5295	*F. teneriffae*	850	*Erica platycodon*	1.000	0.439	0.561	0.000
Fieldwork	14.9	Canary Islands	18/10/2022	-16.2956	28.5295	*F. teneriffae*	850	*Erica platycodon*	1.000	0.706	0.294	0.042
Fieldwork	2.10	Canary Islands	17/10/2022	-16.1697	28.5594	*F. teneriffae*	783	*Erica platycodon*	1.000	0.327	0.673	0.059

Fieldwork	2.11	Canary Islands	17/10/2022	-16.1697	28.5594	*F. teneriffae*	783	*Erica platycodon*	1.000	0.310	0.690	0.056
Fieldwork	2.12	Canary Islands	17/10/2022	-16.1697	28.5594	*F. teneriffae*	783	*Erica platycodon*	0.978	0.540	0.460	0.000
Fieldwork	2.8	Canary Islands	17/10/2022	-16.1697	28.5594	*F. teneriffae*	783	*Erica arborea*	1.000	0.128	0.872	0.000
Fieldwork	2.9	Canary Islands	17/10/2022	-16.1697	28.5594	*F. teneriffae*	783	*Erica platycodon*	1.000	0.694	0.306	0.040
Fieldwork	3.11	Canary Islands	17/10/2022	-16.1778	28.5575	*F. teneriffae*	833	*Laurus novocanariensis*	1.000	0.844	0.156	0.000
Fieldwork	3.12	Canary Islands	17/10/2022	-16.1778	28.5575	*F. teneriffae*	833	*Laurus novocanariensis*	1.000	0.795	0.205	0.000
Fieldwork	3.13	Canary Islands	17/10/2022	-16.1778	28.5575	*F. teneriffae*	833	*Laurus novocanariensis*	1.000	0.294	0.706	0.000
Fieldwork	3.14	Canary Islands	17/10/2022	-16.1778	28.5575	*F. teneriffae*	833	*Laurus novocanariensis*	0.915	0.302	0.698	0.000
Fieldwork	3.5	Canary Islands	17/10/2022	-16.1778	28.5575	*F. teneriffae*	833	*Erica platycodon*	1.000	0.355	0.645	0.000
Fieldwork	4.1	Canary Islands	17/10/2022	-16.1917	28.5519	*F. teneriffae*	701	*Erica platycodon*	0.712	0.757	0.243	0.036
Fieldwork	4.2	Canary Islands	17/10/2022	-16.1917	28.5519	*F. teneriffae*	701	*Genista canariensis*	1.000	0.000	1.000	0.000
Fieldwork	4.3	Canary Islands	17/10/2022	-16.1917	28.5519	*F. teneriffae*	701	*Laurus novocanariensis*	1.000	0.277	0.723	0.000
Fieldwork	4.4	Canary Islands	17/10/2022	-16.1917	28.5519	*F. teneriffae*	701	*Persea barbujana*	0.962	0.760	0.240	0.000
Fieldwork	4.5	Canary Islands	17/10/2022	-16.1917	28.5519	*F. teneriffae*	701	*Erica platycodon*	0.968	1.000	0.000	0.033
Fieldwork	6.5	Canary Islands	19/10/2022	-16.2278	28.5425	*F. teneriffae*	860	*Laurus novocanariensis*	0.733	0.273	0.727	0.000
Fieldwork	6.6	Canary Islands	19/10/2022	-16.2278	28.5425	*F. teneriffae*	860	*Laurus novocanariensis*	1.000	0.262	0.738	0.000
Fieldwork	6.7	Canary Islands	19/10/2022	-16.2278	28.5425	*F. teneriffae*	860	*Viburnum rugosum*	1.000	0.370	0.630	0.000
Fieldwork	6.8	Canary Islands	19/10/2022	-16.2278	28.5425	*F. teneriffae*	860	*Erica platycodon*	1.000	1.000	0.000	0.000
Fieldwork	6.9	Canary Islands	19/10/2022	-16.2278	28.5425	*F. teneriffae*	860	*Prunus lusitanica*	1.000	0.345	0.655	0.000
LD Herbarium	2100911	Iberian Peninsula	06/02/1955	2.669167	42.4728	*F. teneriffae*		*NA*	0.000	0.000	0.000	0.000
LISU Herbarium	160650	Iberian Peninsula	16/10/1990	-9.00819	38.4607	*F. teneriffae*	100	*Arbutus unedo*	0.000	0.000	0.000	0.000

LISU Herbarium	204742	Iberian Peninsula	04/02/2003	-9.46214	38.7685	*F. teneriffae*	413	*Species unknown*	0.318	0.714	0.286	0.000
LISU Herbarium	264267	Iberian Peninsula	07/05/2014	-9.46214	38.7685	*F. teneriffae*	450	*NA*	0.167	0.200	0.800	0.000
LISU Herbarium	264471	Iberian Peninsula	16/04/2015	-9.46214	38.7685	*F. teneriffae*	417	*NA*	0.000	0.000	0.000	0.000
LISU Herbarium	256862	Iberian Peninsula	19/06/2013	-9.46214	38.7685	*F. teneriffae*	480	*Species unknown*	0.167	1.000	0.000	0.000
LISU Herbarium	256861	Iberian Peninsula	18/09/2013	-9.46214	38.7685	*F. teneriffae*	400	*Species unknown*	0.333	0.000	1.000	0.000
LISU Herbarium	264359	Iberian Peninsula	09/03/2015	-9.43922	38.7756	*F. teneriffae*	450	*NA*	0.000	0.000	0.000	0.000
LISU Herbarium	163221	Iberian Peninsula	08/05/1987	-9.39139	38.782	*F. teneriffae*	400	*NA*	0.133	0.500	0.500	0.000
LISU Herbarium	163225	Iberian Peninsula	12/07/1988	-9.43755	38.7844	*F. teneriffae*	300	*Species unknown*	0.000	0.000	0.000	0.000
LISU Herbarium	230879	Iberian Peninsula	15/02/1996	-9.43755	38.7844	*F. teneriffae*		*NA*	0.190	1.000	0.000	0.000
LISU Herbarium	163186	Iberian Peninsula	12/07/1988	-9.38995	38.7852	*F. teneriffae*	450	*Species unknown*	0.000	0.000	0.000	0.000
LISU Herbarium	230876	Iberian Peninsula	14/09/1994	-9.38904	38.792	*F. teneriffae*	436	*NA*	0.000	0.000	0.000	0.000
LISU Herbarium	163222	Iberian Peninsula	02/10/1989	-9.38904	38.792	*F. teneriffae*	436	*NA*	0.000	0.000	0.000	0.000
LISU Herbarium	163223	Iberian Peninsula	05/05/1987	-9.38904	38.792	*F. teneriffae*		*NA*	0.250	1.000	0.000	0.000
LISU Herbarium	163224	Iberian Peninsula	24/10/1988	-9.38904	38.792	*F. teneriffae*	438	*NA*	0.000	0.000	0.000	0.000
LISU Herbarium	230877	Iberian Peninsula	12/02/2005	-9.38904	38.792	*F. teneriffae*		*NA*	0.100	0.000	1.000	0.000
LISU Herbarium	221484	Iberian Peninsula	12/01/2000	-9.38904	38.792	*F. teneriffae*		*NA*	0.286	0.625	0.375	0.000
Fieldwork	S2.1	Iberian Peninsula	09/11/2022	-9.38954	38.7854	*F. teneriffae*	460	*Pinus pinea*	0.565	1.000	0.000	0.000
Fieldwork	S2.2	Iberian Peninsula	09/11/2022	-9.38954	38.7854	*F. teneriffae*	460	*Pinus pinea*	0.636	0.571	0.429	0.000
Fieldwork	S2.3	Iberian Peninsula	09/11/2022	-9.38954	38.7854	*F. teneriffae*	460	*Laurus nobilis*	0.091	1.000	0.000	0.000
Fieldwork	S2.4	Iberian Peninsula	09/11/2022	-9.38954	38.7854	*F. teneriffae*	460	*Acer pseudoplatanus*	0.125	1.000	0.000	0.000
Fieldwork	S2.5	Iberian Peninsula	09/11/2022	-9.38954	38.7854	*F. teneriffae*	460	*Pinus pinea*	0.333	1.000	0.000	0.000

Fieldwork	S4.1	Iberian Peninsula	09/11/2022	-9.46	38.7703	*F. teneriffae*	428	*Cupressus lusitanica*	0.000	0.000	0.000	0.000
Fieldwork	S4.3	Iberian Peninsula	09/11/2022	-9.46	38.7703	*F. teneriffae*	428	*Cupressus lusitanica*	0.000	0.000	0.000	0.000
Fieldwork	S4.4	Iberian Peninsula	09/11/2022	-9.46	38.7703	*F. teneriffae*	428	*Cupressus lusitanica*	0.143	1.000	0.000	0.000
Fieldwork	S4.5	Iberian Peninsula	09/11/2022	-9.46	38.7703	*F. teneriffae*	428	*Cupressus lusitanica*	0.000	0.000	0.000	0.000
Fieldwork	S4.7	Iberian Peninsula	09/11/2022	-9.46	38.7703	*F. teneriffae*	428	*Cupressus lusitanica*	0.211	1.000	0.000	0.000
E Herbarium	E00831424	Iberian Peninsula	31/05/2016	-5.54623	36.1027	*F. teneriffae*	720	*Species unknown*	0.643	0.556	0.444	0.000
LISU Herbarium	165823	Madeira	10/05/1990	-16.9207	32.7211	*F. teneriffae*	1550	*Species unknown*	0.643	1.000	0.000	0.000
LISU Herbarium	166336	Madeira	27/07/1951	-16.8918	32.7086	*F. teneriffae*		*NA*	0.167	1.000	0.000	0.000
LISU Herbarium	260950	Madeira	08/08/2007	-16.9098	32.7122	*F. teneriffae*	1530	*Laurus novocanariensis*	1.000	0.118	0.882	0.000
LISU Herbarium	261166	Madeira	01/08/2007	-17.1233	32.7178	*F. teneriffae*	950	*Persea indica*	0.000	0.000	0.000	0.000
LISU Herbarium	173621	Madeira	02/05/1997	-16.8994	32.7214	*F. teneriffae*		*Species unknown*	1.000	0.579	0.421	0.091
LISU Herbarium	165816	Madeira	12/10/1990	-16.8994	32.7214	*F. teneriffae*	890	*Species unknown*	1.000	1.000	0.000	0.000
LISU Herbarium	261119	Madeira	28/07/2006	-17.1342	32.7267	*F. teneriffae*	830	*Persea indica*	0.250	0.000	1.000	0.000
LISU Herbarium	260886	Madeira	17/11/2005	-16.9209	32.7301	*F. teneriffae*	960	*Clethra arborea*	1.000	0.429	0.571	0.000
LISU Herbarium	261025	Madeira	21/07/2005	-16.9102	32.7303	*F. teneriffae*	900	*Clethra arborea*	0.400	0.000	1.000	0.000
LISU Herbarium	165821	Madeira	09/10/1990	-16.8996	32.7304	*F. teneriffae*	890	*Species unknown*	1.000	0.500	0.500	0.000
LISU Herbarium	260959	Madeira	10/08/2007	-16.8889	32.7306	*F. teneriffae*	850	*Myrica faya*	1.000	0.583	0.417	0.000
LISU Herbarium	162423	Madeira	30/05/1989	-16.9283	32.7344	*F. teneriffae*		*Species unknown*	0.167	1.000	0.000	0.000
LISU Herbarium	254076	Madeira	24/07/2003	-16.9211	32.7391	*F. teneriffae*	950	*NA*	1.000	1.000	0.000	0.000
LISU Herbarium	165822	Madeira	10/05/1990	-16.8784	32.7398	*F. teneriffae*	850	*Species unknown*	1.000	0.938	0.063	0.267
LISU Herbarium	166351	Madeira	09/05/1982	-16.8784	32.7398	*F. teneriffae*		*Species unknown*	0.875	1.000	0.000	0.000
LISU Herbarium	254664	Madeira	02/08/2004	-16.9426	32.7478	*F. teneriffae*	1700	*NA*	0.167	1.000	0.000	0.200
LISU Herbarium	166353	Madeira	13/05/1979	-17.0161	32.7603	*F. teneriffae*		*Species unknown*	0.459	0.588	0.412	0.100

LISU Herbarium	166352	Madeira	11/05/1982	-17.0161	32.7603	*F. teneriffae*		*Species unknown*	0.167	1.000	0.000	0.000
LISU Herbarium	166347	Madeira	11/05/1982	-17.0161	32.7603	*F. teneriffae*		*NA*	0.545	0.167	0.833	0.000
LISU Herbarium	261227	Madeira	25/11/2005	-17.0604	32.764	*F. teneriffae*	1230	*Myrica faya*	1.000	0.750	0.250	0.000
LISU Herbarium	165818	Madeira	11/10/1990	-17.0604	32.764	*F. teneriffae*	1050	*Species unknown*	0.839	0.885	0.115	0.000
LISU Herbarium	166359	Madeira	28/06/1988	-17.0616	32.7684	*F. teneriffae*	1100	*Species unknown*	0.464	0.923	0.077	0.000
LISU Herbarium	166362	Madeira	28/06/1988	-17.0772	32.7696	*F. teneriffae*	1580	*Species unknown*	0.348	0.625	0.375	0.000
LISU Herbarium	166363	Madeira	28/06/1988	-17.0772	32.7696	*F. teneriffae*	1580	*Species unknown*	0.833	0.200	0.800	0.000
LISU Herbarium	165815	Madeira	13/10/1990	-17.2206	32.7703	*F. teneriffae*	670	*NA*	0.778	0.571	0.429	0.000
LISU Herbarium	254549	Madeira	11/06/2004	-17.0713	32.7728	*F. teneriffae*	1150	*NA*	0.167	0.000	1.000	0.000
LISU Herbarium	249936	Madeira	05/08/2002	-17.0713	32.7728	*F. teneriffae*	1000	*NA*	0.500	0.000	1.000	0.000
LISU Herbarium	261242	Madeira	24/07/2006	-16.9752	32.7744	*F. teneriffae*	635	*Persea indica*	0.714	0.800	0.200	0.000
LISU Herbarium	261149	Madeira	01/08/2006	-16.9752	32.7744	*F. teneriffae*	600	*Laurus novocanariensis*	0.667	0.500	0.500	0.000
LISU Herbarium	249934	Madeira	02/08/2002	-16.9219	32.7752	*F. teneriffae*	900	*NA*	1.000	0.615	0.385	0.000
LISU Herbarium	148962	Madeira	27/10/1981	-17.1248	32.7809	*F. teneriffae*		*Species unknown*	1.000	0.000	1.000	0.000
LISU Herbarium	261093	Madeira	05/08/2004	-17.0928	32.7815	*F. teneriffae*	1000	*Erica arborea*	1.000	0.500	0.500	0.000
LISU Herbarium	254481	Madeira	06/04/2004	-17.0928	32.7815	*F. teneriffae*	1400	*NA*	1.000	0.867	0.133	0.077
LISU Herbarium	166377	Madeira	07/05/1982	-16.9754	32.7834	*F. teneriffae*		*NA*	0.080	1.000	0.000	0.000
LISU Herbarium	260899	Madeira	23/11/2005	-16.9327	32.784	*F. teneriffae*	770	*Erica scoparia*	0.118	1.000	0.000	0.000
LISU Herbarium	260976	Madeira	19/07/2005	-17.1784	32.789	*F. teneriffae*	1250	*Clethra arborea*	0.545	0.417	0.583	0.000
LISU Herbarium	165817	Madeira	11/10/1990	-17.1357	32.7898	*F. teneriffae*	1200	*Species unknown*	0.700	0.571	0.429	0.000
LISU Herbarium	261028	Madeira	22/07/2005	-17.093	32.7905	*F. teneriffae*	1000	*Heberdenia excelsa*	0.429	0.667	0.333	0.000
LISU Herbarium	261185	Madeira	22/07/2005	-17.0823	32.7907	*F. teneriffae*	850	*Ocotea foetens*	1.000	0.250	0.750	0.000
LISU Herbarium	166360	Madeira	12/04/1988	-16.9116	32.7934	*F. teneriffae*	575	*NA*	0.219	1.000	0.000	0.000
LISU Herbarium	261062	Madeira	28/07/2004	-17.1359	32.7988	*F. teneriffae*	1200	*Clethra arborea*	0.929	0.538	0.462	0.000
LISU Herbarium	260906	Madeira	24/11/2005	-17.1252	32.799	*F. teneriffae*	1135	*Erica arborea*	0.588	0.600	0.400	0.000

LISU Herbarium	166365	Madeira	28/06/1988	-17.1252	32.799	*F. teneriffae*	1250	*Species unknown*	0.500	0.600	0.400	0.000
LISU Herbarium	166361	Madeira	28/06/1988	-17.1252	32.799	*F. teneriffae*	1250	*Species unknown*	0.464	0.231	0.769	0.000
LISU Herbarium	260912	Madeira	02/08/2007	-16.9758	32.8014	*F. teneriffae*	500	*Myrica faya*	1.000	0.250	0.750	0.000
LISU Herbarium	242195	Madeira	27/06/2005	-17.2002	32.8067	*F. teneriffae*	1040	*NA*	0.273	1.000	0.000	0.000
Fieldwork	MD1.1	Madeira	29/11/2022	-17.1405	32.8101	*F. teneriffae*	1398	*Laurus novocanariensis*	0.911	0.805	0.195	0.030
Fieldwork	MD1.2	Madeira	29/11/2022	-17.1405	32.8101	*F. teneriffae*	1398	*Ocotea foetens*	1.000	0.182	0.818	0.000
Fieldwork	MD1.3	Madeira	29/11/2022	-17.1405	32.8101	*F. teneriffae*	1398	*Ocotea foetens*	0.375	0.333	0.667	0.000
Fieldwork	MD1.4	Madeira	29/11/2022	-17.1405	32.8101	*F. teneriffae*	1398	*Ocotea foetens*	0.400	1.000	0.000	0.000
Fieldwork	MD1.5	Madeira	29/11/2022	-17.1405	32.8101	*F. teneriffae*	1398	*Laurus novocanariensis*	1.000	0.458	0.542	0.091
Fieldwork	MD15.1	Madeira	28/11/2022	-16.9206	32.7803	*F. teneriffae*	850	*Laurus novocanariensis*	0.955	0.476	0.524	0.000
Fieldwork	MD15.2	Madeira	28/11/2022	-16.9206	32.7803	*F. teneriffae*	850	*Genista canariensis*	0.857	0.389	0.611	0.095
Fieldwork	MD15.3	Madeira	28/11/2022	-16.9206	32.7803	*F. teneriffae*	850	*Erica platycodon*	0.722	0.231	0.769	0.000
Fieldwork	MD15.4	Madeira	28/11/2022	-16.9206	32.7803	*F. teneriffae*	850	*Laurus novocanariensis*	0.750	0.424	0.576	0.000
Fieldwork	MD15.5	Madeira	28/11/2022	-16.9206	32.7803	*F. teneriffae*	850	*Erica platycodon*	1.000	0.974	0.026	0.000
Fieldwork	MD17.2	Madeira	30/11/2022	-16.9769	32.77	*F. teneriffae*	1074	*Clethra arborea*	1.000	0.810	0.190	0.059
Fieldwork	MD17.3	Madeira	30/11/2022	-16.9769	32.77	*F. teneriffae*	1074	*Clethra arborea*	1.000	0.333	0.667	0.000
Fieldwork	MD17.4	Madeira	30/11/2022	-16.9769	32.77	*F. teneriffae*	1074	*Clethra arborea*	0.936	0.288	0.712	0.095
Fieldwork	MD17.5	Madeira	30/11/2022	-16.9769	32.77	*F. teneriffae*	1074	*Ocotea foetens*	0.235	0.750	0.250	0.000
Fieldwork	MD17.6	Madeira	30/11/2022	-16.9769	32.77	*F. teneriffae*	1074	*Ocotea foetens*	0.667	0.875	0.125	0.000
Fieldwork	MD18.1	Madeira	30/11/2022	-16.9797	32.7775	*F. teneriffae*	670	*Laurus novocanariensis*	0.700	1.000	0.000	0.143
Fieldwork	MD18.2	Madeira	30/11/2022	-16.9797	32.7775	*F. teneriffae*	670	*Laurus novocanariensis*	0.833	0.733	0.267	0.000
Fieldwork	MD18.3	Madeira	30/11/2022	-16.9797	32.7775	*F. teneriffae*	670	*Laurus novocanariensis*	0.735	0.640	0.360	0.000
Fieldwork	MD18.5	Madeira	30/11/2022	-16.9797	32.7775	*F. teneriffae*	670	*Laurus novocanariensis*	1.000	1.000	0.000	0.000

Fieldwork	MD18.6	Madeira	30/11/2022	-16.9797	32.7775	*F. teneriffae*	670	*Laurus novocanariensis*	1.000	0.600	0.400	0.000
Fieldwork	MD6.10	Madeira	02/12/2022	-16.9102	32.7436	*F. teneriffae*	730	*Clethra arborea*	0.647	0.864	0.136	0.053
Fieldwork	MD6.4	Madeira	02/12/2022	-16.9102	32.7436	*F. teneriffae*	730	*Laurus novocanariensis*	1.000	1.000	0.000	0.000
Fieldwork	MD6.5	Madeira	02/12/2022	-16.9102	32.7436	*F. teneriffae*	730	*Laurus novocanariensis*	0.450	0.667	0.333	0.000
Fieldwork	MD6.6	Madeira	02/12/2022	-16.9102	32.7436	*F. teneriffae*	730	*Laurus novocanariensis*	1.000	1.000	0.000	0.100
Fieldwork	MD6.8	Madeira	02/12/2022	-16.9102	32.7436	*F. teneriffae*	730	*Laurus novocanariensis*	1.000	1.000	0.000	0.000
Fieldwork	MD8.2	Madeira	01/12/2022	-17.0186	32.7536	*F. teneriffae*	1007	*Vaccinium padifolium*	0.935	0.552	0.448	0.000
Fieldwork	MD8.3	Madeira	01/12/2022	-17.0186	32.7536	*F. teneriffae*	1007	*Erica platycodon*	1.000	0.725	0.275	0.069
Fieldwork	MD8.4	Madeira	01/12/2022	-17.0186	32.7536	*F. teneriffae*	1007	*Erica platycodon*	1.000	0.757	0.243	0.071
Fieldwork	MD8.6	Madeira	01/12/2022	-17.0186	32.7536	*F. teneriffae*	1007	*Laurus novocanariensis*	1.000	0.083	0.917	0.000
Fieldwork	MD8.9	Madeira	01/12/2022	-17.0186	32.7536	*F. teneriffae*	1007	*Laurus novocanariensis*	1.000	0.114	0.886	0.000

Table S4. The mean sex expression (SE) and phenotypic sex ratio (PSR ♀: females; and ♂: males), per species and region for both herbarium and fieldwork samples. The results of the Mann-Whitney-Wilcoxon (p-value) are presented, with significance levels adjusted for multiple comparisons using Bonferroni corrections. This analysis assessed the consistency of sex expression (SE) and the female-to-male phenotypic sex ratio (PSR♀ and PSR♂) between herbarium and fieldwork samples. (N) number of samples analysed per region.

		Herbarium				Fieldwork				p-value
	Region	N	SE	PSR♀	PSR♂	N	SE	PSR♀	PSR♂	> 0.05
E. intermedia	Canary Islands	24	0.64	0.65	0.18	40	0.31	0.45	0.16	> 0.05
	Madeira	32	0.25	0.64	0.11	85	0.24	0.5	0.11	> 0.05
F. polysticta	Canary Islands	30	0.86	0.80	0.20	20	0.56	0.63	0.33	> 0.05
	Madeira	33	0.80	0.81	0.12	55	0.85	0.83	0.13	> 0.05
F. teneriffae	Canary Islands	51	0.81	0.47	0.50	30	0.97	0.49	0.52	> 0.05
	Madeira	46	0.63	0.60	0.38	30	0.83	0.63	0.36	> 0.05
	Iberian Peninsula	17	0.14	0.29	0.23	10	0.21	0.65	0.48	> 0.05
P. canariensis	Canary Islands	61	0.48	0.55	0.37	60	0.66	0.43	0.44	> 0.05
	Madeira	39	0.60	0.73	0.24	85	0.85	0.68	0.32	> 0.14
	Iberian Peninsula	33	0.30	0.57	0.19	10	0.24	0.82	0.10	> 0.05

Table S5. Results of the Shapiro-Wilk normality test to determine whether the data follow a normal distribution or not.

	Shapiro-Wilk normality test		
	SE	PSR♀	PSR♂
E. intermedia	W = 0.83043, p-value = 2.599e-14	W = 0.68414, p-value < 2.2e-16	W = 0.53582, p-value < 2.2e-16
F. polysticta	W = 0.7726, p-value = 2.369e-13	W = 0.70694, p-value = 2.971e-15	W = 0.68768, p-value = 9.411e-16
F. teneriffae	W = 0.81298, p-value < 2.2e-16	W = 0.81572, p-value < 2.2e-16	W = 0.76611, p-value < 2.2e-16
P. canariensis	W = 0.86632, p-value = 5.956e-16	W = 0.78268, p-value < 2.2e-16	W = 0.74415, p-value < 2.2e-16

Table S6. Results of the Kruskal-Wallis test used to investigate whether the type of phorophyte (tree species) affected sex expression (SE) and the phenotypic female-to-male sex ratio (PSR♀ and PSR♂) in the fieldwork samples. (N) number of samples analysed per region.

	**Region**	**SE**	**PSR♀**	**PSR♂**
E. intermedia	Canary Islands (N = 40)	Kruskal-Wallis, p = 0.761	Kruskal-Wallis, p = 0.757	Kruskal-Wallis, p = 0.725
	Madeira (N = 85)	Kruskal-Wallis, p = 0.725	Kruskal-Wallis, p = 0.725	Kruskal-Wallis, p = 0.725
F. polysticta	Canary Islands (N = 20)	Kruskal-Wallis, p = 0.258	Kruskal-Wallis, p = 0.478	Kruskal-Wallis, p = 0.478
	Madeira (N = 55)	Kruskal-Wallis, p = 0.431	Kruskal-Wallis, p = 0.287	Kruskal-Wallis, p = 0.379
F. teneriffae	Canary Islands (N = 30)	Kruskal-Wallis, p = 0.713	Kruskal-Wallis, p = 0.142	Kruskal-Wallis, p = 0.142
	Madeira (N = 30)	Kruskal-Wallis, p = 0.223	Kruskal-Wallis, p = 0.932	Kruskal-Wallis, p = 0.932
	Iberian Peninsula (N = 10)	Kruskal-Wallis, p = 0.111	Kruskal-Wallis, p = 0.449	Kruskal-Wallis, p = 0.506
P. canariensis	Canary Islands (N = 60)	Kruskal-Wallis, p = 0.841	Kruskal-Wallis, p = 0.381	Kruskal-Wallis, p = 0.464
	Madeira (N = 85)	Kruskal-Wallis, p = 0.499	Kruskal-Wallis, p = 0.725	Kruskal-Wallis, p = 0.725
	Iberian Peninsula (N = 10)	Kruskal-Wallis, p = 0.917	Kruskal-Wallis, p = 0.136	Kruskal-Wallis, p = 0.317

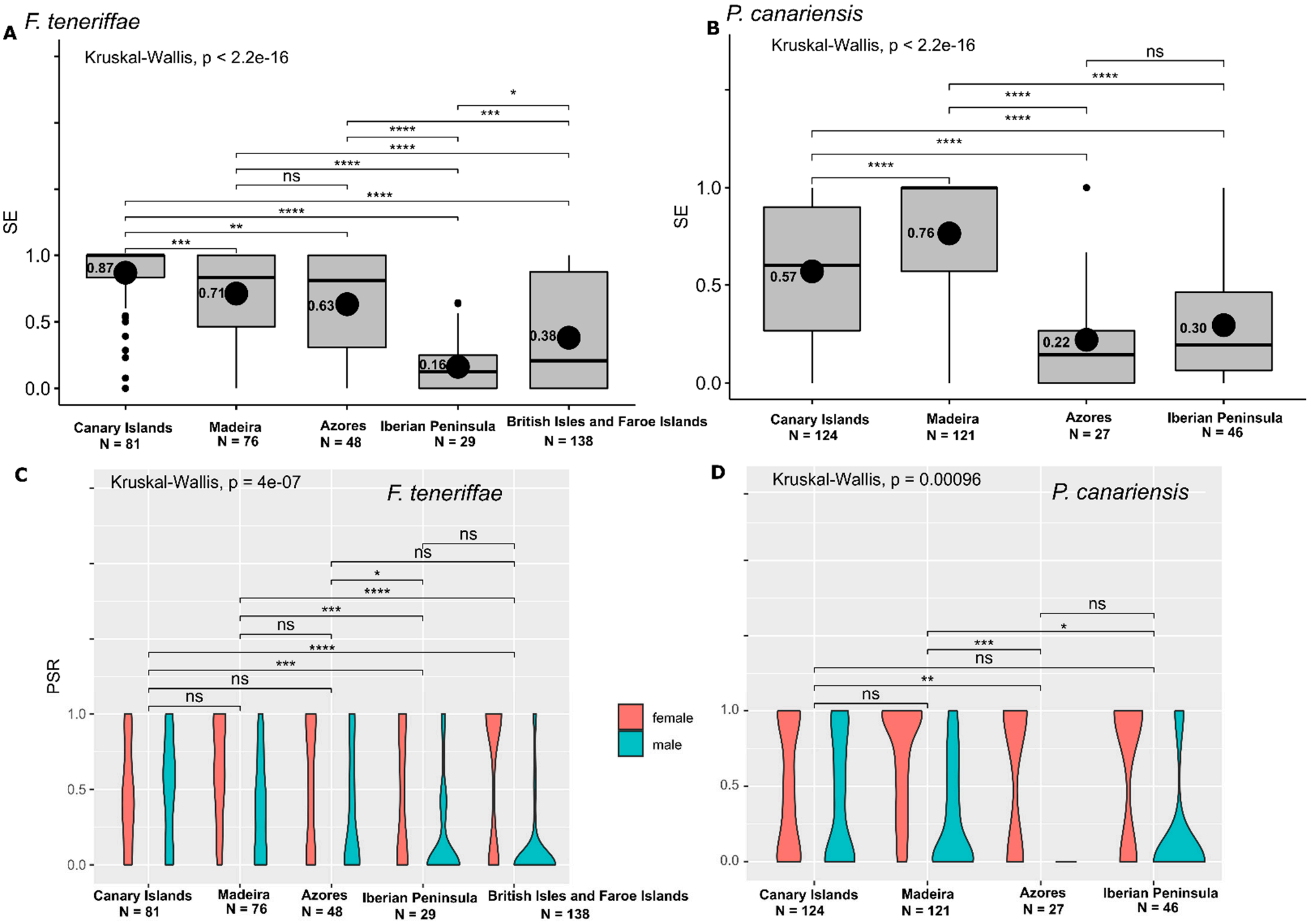

Figure S1. A–B, Box plots with Kruskal-Wallis and Wilcoxon tests, displaying sex expression (SE) results for each species and region (****p < 0.0001, *** p < 0.001, ** p < 0.01, *p < 0.05, ns indicates a non-significant difference). Horizontal black lines denote median values, and the mean value is represented by a black dot the respective number displayed on the left. (C–D) Bean plots with Kruskal-Wallis and Wilcoxon tests, displaying phenotypic sex ratio (PSR) results for each species and region (****p < 0.0001, *** p < 0.001, ** p < 0.01, *p < 0.05, ns indicates a non-significant difference). N, number of samples analyzed per locality. (**A, C**), *Frullania teneriffae*. (**B, D**), *Porella canariensis*.

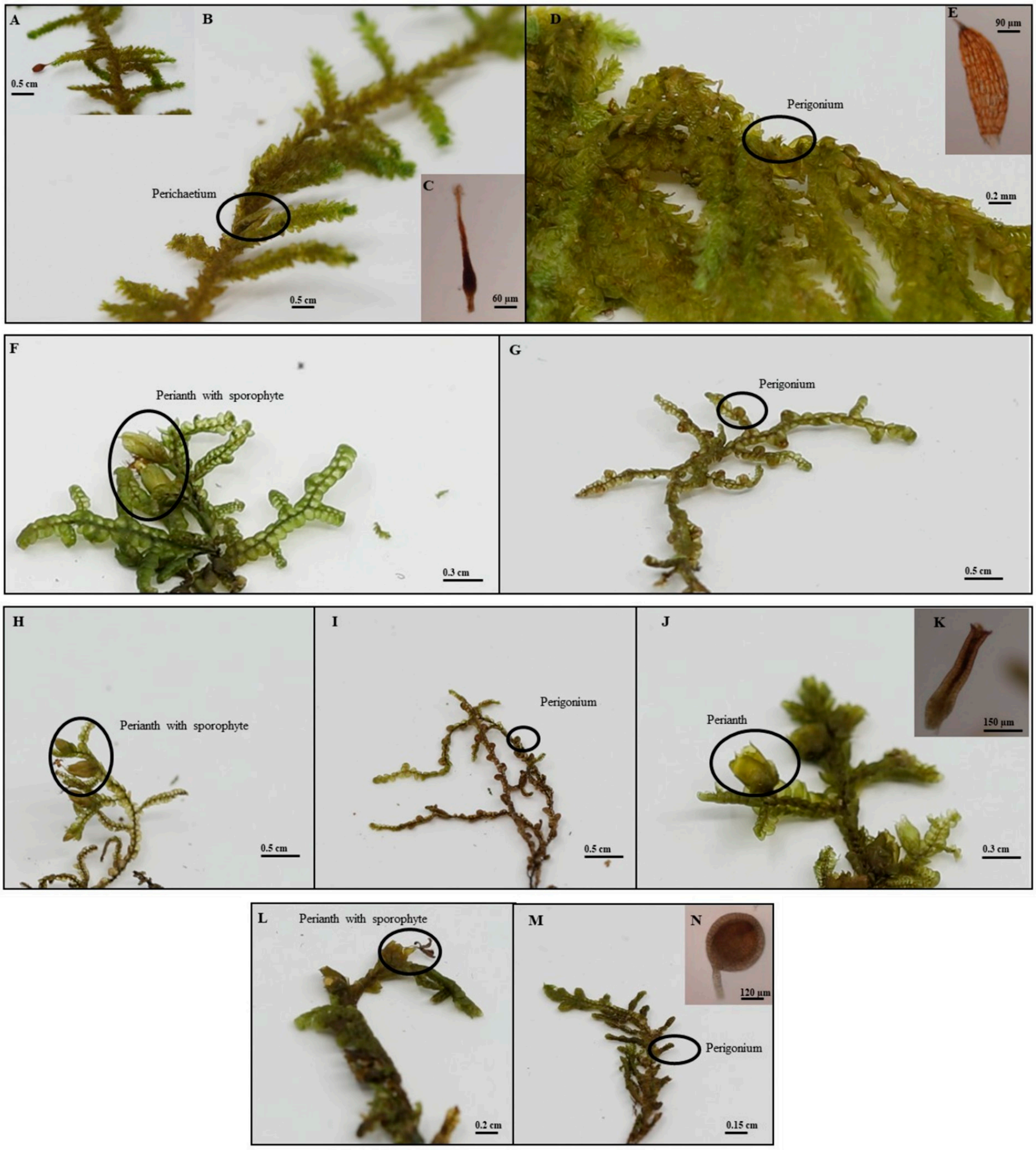

Figure S2. A–D, photos of *E. intermedia* reproductive structures. **A**, female shoot with sporophyte. **B**, female shoot with perichaetium: structure containing the female gametangia. **C**, archegonium: mosses female gametangium (flask-shaped). **D**, male shoot with perigonium: structure containing the male gametangia. **E**, antheridium: mosses male gametangium (fusiform shaped). **F–G**, photos of *F. polysticyta* reproductive structures. **F**, female shoot with perianth: tubular structure that surrounds the imature sporophyte of liverworts. **G**, male shoot with

perigonium. **H–I**, photos of *F. teneriffae* reproductive structures. **H**, female shoot with perianth and sporophyte. **I**, male shoot with perigonium. **J–N**, photos of *P. canariensis* reproductive structures. **J**, female shoot with perianth. **K**, archegonium: liverworts female gametangium (flask-shaped). **L**, female shoot with sporophyte. **M**, male shoot with perigonium. **N**, antheridium: liverworts male gametangium (spherical shaped).
